# Supplementary material for: Niche conservatism and convergence in birds of three cenocrons in the Mexican Transition Zone
Source: PeerJ. 2024 Jan 2;12:e16664. doi: 10.7717/peerj.16664 (PMC10768671; doi:10.7717/peerj.16664)

**SUPLEMENTARY MATERIAL**

**Appendix 1**

[EXTENDED METHOD 3](#_Toc137463432)

[1. Data sources and pre-process 3](#_Toc137463433)

[Taxonomical treatment 3](#_Toc137463434)

[Biogeographic information 3](#_Toc137463435)

[Phylogenetic data 8](#_Toc137463436)

[Known distribution 9](#_Toc137463437)

[Occurrence data 10](#_Toc137463438)

[Occurrence clean up 11](#_Toc137463439)

[Environmental variables 11](#_Toc137463440)

[2. Species selection 16](#_Toc137463441)

[Code 16](#_Toc137463442)

[3. Finding related clades 18](#_Toc137463443)

[Code 19](#_Toc137463444)

[4. Assigning selected species to a cenocron 20](#_Toc137463445)

[Code 21](#_Toc137463446)

[5. Occurrence data cleaning 24](#_Toc137463447)

[Code 24](#_Toc137463448)

[6. Species Distribution Model Building 29](#_Toc137463449)

[Code 30](#_Toc137463450)

[EXTENDED RESULTS 36](#_Toc137463451)

[1. Principal Components Analysis of environmental variables 36](#_Toc137463452)

[Variables used 36](#_Toc137463453)

## EXTENDED METHOD

### Data sources and pre-process

#### Taxonomical treatment

The species names were standardized to match the label found in the phylogeny used. This was done using the name backbone function in rgbif package. The phylogeny follows the Birdlife V3 world list (9,895 extant species recognized). Each label was assigned with GBIF’s unique SpeciesKey which identifies the valid species. The speciesKey allowed us to join occurrences from CONABIO’s database, the phylogeny, mountain birds elevational ranges, and Bird Life known distribution polygons.

#### Biogeographic information

We added the main biogeographical distribution of each polygon of the BirdLife distribution maps (BirdLife International and Handbook of the Birds of the World, 2020). This was done using biogeographic regionalization for the American Continent and the WWF regions for the rest of the world. The amount of oceanic distribution was also measured. It resulted in a database of 70 columns (Table S1) of taxonomical and distributional data from different sources. The selection of species and the cenocron membership is based on this database.

Table S1. Attributes included in the biogeographic information database of Mexican Transition Zone birds.

| **Field** | **Classification** | **Source** | **Description** |
| --- | --- | --- | --- |
| speciesKey | Taxonomic | GBIF | Unique species ID in GBIF taxonomical backbone for the label used in Jetz (2015) |
| OBJECTID | Distribution | Bird Life | ID of the polygon from the Brid Life range maps |
| binomial_BL | Taxonomic | Bird Life | Binomial species names accepted by Bird Life |
| binomial_jetz | Taxonomic | Jetz et al. (2015) | Binomial species names in the phylogeny |
| label | Taxonomic | Jetz et al. (2015) | Label in the phylogeny |
| binomial_gbif | Taxonomic | GBIF | Binomial species names accepted by GBIF |
| PatchClade | Taxonomic | Jetz et al. (2015) | higher-level relationships *sensu* Jetz (2015) |
| Hackett_FineClades | Taxonomic | Jetz et al. (2015) | higher-level relationships in birds (Hackett et al. 2008) |
| Hackett_CoarseClades | Taxonomic | Jetz et al. (2015) | higher-level relationships in birds (Hackett et al. 2008) |
| BLFamilyEnglish | Common Name | Jetz et al. (2015) | Common Name for the family |
| English | Common Name | Jetz et al. (2015) | Common name in English *sensu* Bird Life |
| presence | Distribution | Bird Life | 1 = Extant, 2 = Probably Extant, 3 = Possibly Extant, 4 = Possibly Extinct, 4 = Extinct (post 1500), 6 = Presence Uncertain |
| origin | Distribution | Bird Life | 1 = Native, 2 = Reintroduced, 3 = Introduced, 4 = Vagrant, 5 = Origin Uncertain, 6 = Assisted Colonisation |
| seasonal | Distribution | Bird Life | 1 = Resident, 2 = Breeding Season, 3 = Non-breeding Season, 4 = Passage, 5 = Seasonal occurrence uncertain |
| dist_comm | Distribution | Bird Life | Remarks about the distribution |
| Shape_Length | Distribution | Bird Life | Latitudinal length of distribution |
| Shape_Area | Distribution | Bird Life | Distribution area size |
| IOCOrder | Taxonomic | Jetz et al. (2015) | Order *sensu* IOC Version 2.7 (IOC27) |
| BLFamilyLatin | Taxonomic | Jetz et al. (2015) | Bird Life V3 Family names in Latin |
| genus | Taxonomic | GBIF | Genus in GBIF |
| species | Taxonomic | GBIF | Specific epiteth in GBIF |
| PassNonPass | Taxonomic | Jetz et al. (2015) | Binary: Passeriformes or not Passeriformes grouping |
| OscSubOsc | Taxonomic | Jetz et al. (2015) | Oscine or SubOscine sensu Sibley & Monroe (1990) |
| EnMexico | Distribution | CONABIO | Binary: Its distribution includes Mexico. |
| binomial_mx | Taxonomic | CONABIO | Binomial species names accepted by CONABIO |
| Nombre.Español | Common Name | CONABIO | Common name in Spanish |
| Nombre.Inglés | Common Name | CONABIO | Common name in English *sensu* CONABIO |
| Endemismo | Distribution | CONABIO | Is it Endemic, Semi-endemic, or Cuasi-endemic in Mexico? |
| Residente | Distribution | CONABIO | Binary: Resident in Mexico |
| Transitoria | Distribution | CONABIO | Binary: Vagrant in Mexico |
| Migratoria | Distribution | CONABIO | Binary: Migratory |
| Endemica | Distribution | CONABIO | Binary: It hasany endemism category |
| Mountain_ID | Distribution | Quintero & Jetz (2018) | Mountain identificator |
| Min_elevation | Distribution | Quintero & Jetz (2018) | Minimum elevation for the species |
| Max_elevation | Distribution | Quintero & Jetz (2018) | maximum elevation for the species |
| Continent | Distribution | Quintero & Jetz (2018) | Continent where the Mountain range is found |
| Mountain.Range | Distribution | Quintero & Jetz (2018) | Mountain Range Name |
| Uplift_Start | Distribution | Quintero & Jetz (2018) | Age when the Mountain Range started to lift |
| Mountains | Distribution | Quintero & Jetz (2018) | Binary: is distributed in mountains? |
| Palearctic | Biogeographic Regionalization | Morrone (2014) & WWF | Percentage of distribution in this region |
| Northern.Nearctic | Biogeographic Regionalization | Morrone (2014) & WWF | Percentage of distribution in this region |
| Nearctic.Region | Biogeographic Regionalization | Morrone (2014) & WWF | Percentage of distribution in this region |
| Mexican.Transition.Zone | Biogeographic Regionalization | Morrone (2014) & WWF | Percentage of distribution in this region |
| Mesoamerican.dominion | Biogeographic Regionalization | Morrone (2014) & WWF | Percentage of distribution in this region |
| Pacific.dominion | Biogeographic Regionalization | Morrone (2014) & WWF | Percentage of distribution in this region |
| Antillean.subregion | Biogeographic Regionalization | Morrone (2014) & WWF | Percentage of distribution in this region |
| Boreal.Brazilian.dominion | Biogeographic Regionalization | Morrone (2014) & WWF | Percentage of distribution in this region |
| South.Brazilian.dominion | Biogeographic Regionalization | Morrone (2014) & WWF | Percentage of distribution in this region |
| South.eastern.Amazonian.dominion | Biogeographic Regionalization | Morrone (2014) & WWF | Percentage of distribution in this region |
| Chacoan.dominion | Biogeographic Regionalization | Morrone (2014) & WWF | Percentage of distribution in this region |
| Parana.dominion | Biogeographic Regionalization | Morrone (2014) & WWF | Percentage of distribution in this region |
| South.American.Transition.Zone | Biogeographic Regionalization | Morrone (2014) & WWF | Percentage of distribution in this region |
| Afrotropical | Biogeographic Regionalization | Morrone (2014) & WWF | Percentage of distribution in this region |
| Madagascan | Biogeographic Regionalization | Morrone (2014) & WWF | Percentage of distribution in this region |
| Sino.Japanese | Biogeographic Regionalization | Morrone (2014) & WWF | Percentage of distribution in this region |
| Oriental | Biogeographic Regionalization | Morrone (2014) & WWF | Percentage of distribution in this region |
| Saharo.Arabian | Biogeographic Regionalization | Morrone (2014) & WWF | Percentage of distribution in this region |
| Australian | Biogeographic Regionalization | Morrone (2014) & WWF | Percentage of distribution in this region |
| Andean.region | Biogeographic Regionalization | Morrone (2014) & WWF | Percentage of distribution in this region |
| Australasia | Biogeographic Regionalization | Morrone (2014) & WWF | Percentage of distribution in this region |
| Ocean | Biogeographic Regionalization | Morrone (2014) & WWF | Percentage of distribution in this the Ocean |
| Nearctic_TOTAL | Biogeographic Regionalization | Morrone (2014) & WWF | Total percentage of distribution in this Realm |
| Neotropics_TOTAL | Biogeographic Regionalization | Morrone (2014) & WWF | Total percentage of distribution in this Realm |
| Palearctic_REALM | Biogeographic Regionalization | Morrone (2014) & WWF | Total percentage of distribution in this Realm |
| Paleotropical_REALM | Biogeographic Regionalization | Morrone (2014) & WWF | Total percentage of distribution in this Realm |
| Austral | Biogeographic Regionalization | Morrone (2014) & WWF | Total percentage of distribution in this Realm |
| Mexican_TOTAL | Biogeographic Regionalization | Morrone (2014) & WWF | Total percentage of distribution in MTZ |
| Subregion | Biogeographic Regionalization | Morrone (2014) & WWF | Sub region where most of the distribution is found |
| Realm | Biogeographic Regionalization | Morrone (2014) & WWF | Realm where most of the distribution is found |
| ZTM_end | Biogeographic Regionalization | Morrone (2014) & WWF | Level of endemism in the MTZ (1 to 7) |

#### Phylogenetic data

We downloaded from BirdTree.org the last set of the stage two trees with the Hackett backbone available here. The original phylogeny includes all the 9.993 species described by the time of its publication. We used a prunned phylogeny (Fig. S1) with the selected species listed in Appendix 2. We ran all the analysis through the 1000 trees set to include phylogenetic uncertainty. The resulting nexus file is available upon request.


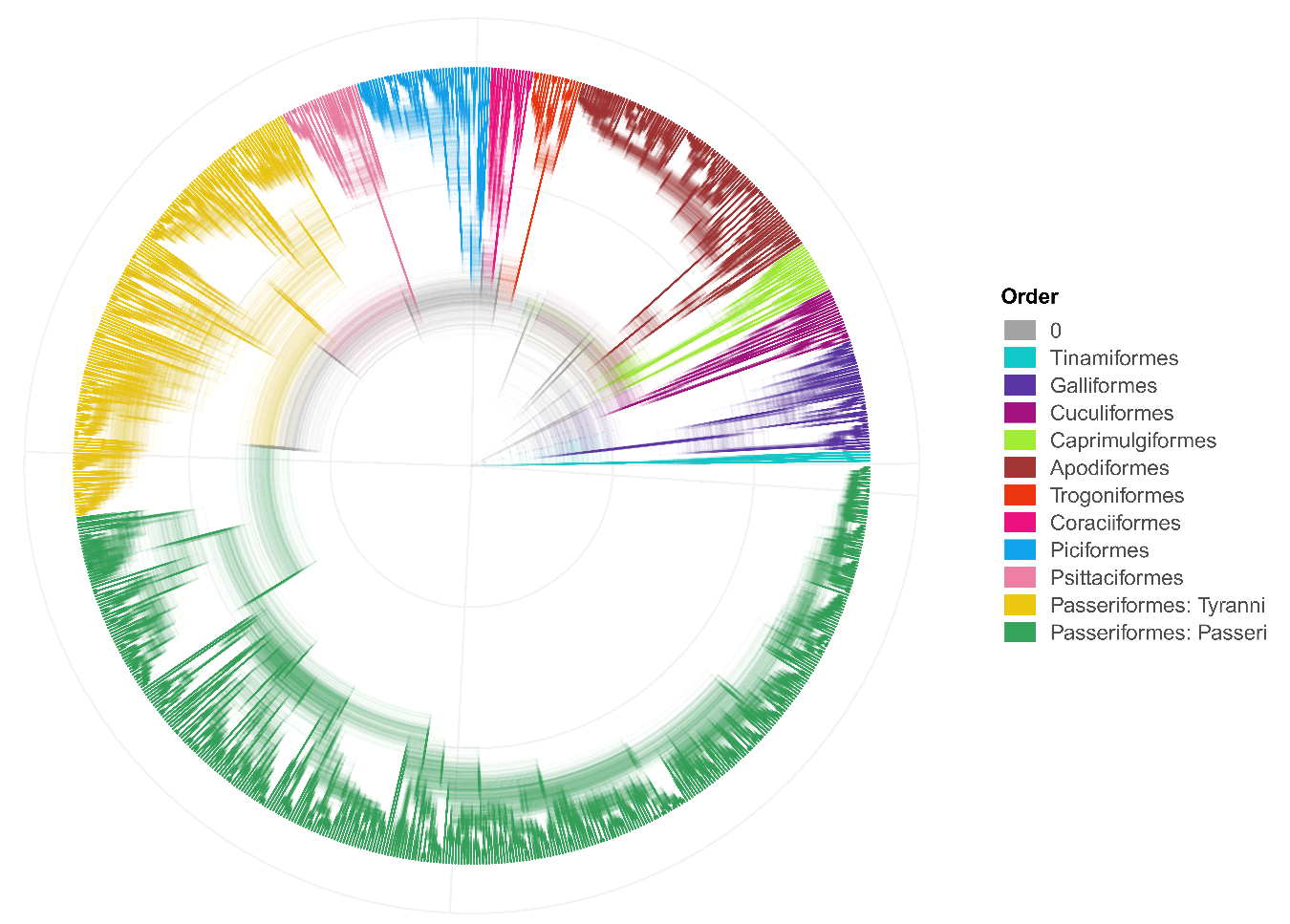


Fig. S1. Phylogenetic dataset used, modified from Jetz et.al (2012). It includes 1000 trees with the selected species.

#### Known distribution

Using the known distribution was obtained from BirdLife International, which includes information from the Handbook of the Birds of the World (BirdLife International and Handbook of the Birds of the World, 2020). This is available at BirdLife Data Zone. These database contains information on the type of distribution being presented by each polygon in 3 separate fields:

- - Presence: The level of certainty we have as to the species existence in an area.
  - Origin: Whether that species occurs in an area naturally.
  - Seasonality: The period in the species life cycle in which it occurs in an area.

We used native breeding season and resident distribution, with certain presence. Resulting polygons were dissolved into one area per species to perform the next steps.

#### Occurrence data

##### CONABIO’s database

We downloaded data from CONABIO’s geoportal. That bird database includes records of Mexican species from museum, open data sources, and from projects funded by CONABIO. Is restricted to occurrence inside the political borders of Mexico and records in Mexican institutions. We filtered the database to include only the selected species and processed to clean up the occurrence. After the clean-up, species with less than 100 unique occurrences in this database were obtained from GBIF’s database.

##### GBIF’s database

We use the *rgbif* package to search for occurrences in GBIF. This package allows us to search for a large number of species names, while the downloads through the web interface are limited to around 200 names (taxonkeys), and allows complex downloads with filters. But, the most important thing of all, it gives a citable doi for each download that indicates the search parameter. The occurrence query was performed in two steps. First, we looked for species in CONABIO’s database with less than 100 occurrences. Then all the selected species, but only occurrences outside of Mexico. Both queries had the next parameters:

- Matched bird species from “taxonKey”
- With these basis of record: Preserved specimen, Human Observation, and Observation
- Has coordinates
- Has no gbif geospatial issues
- Is greater than or equal to year 1950
- Occurrence status is not absent

Since the species list is long and the occurrences are abundant, we split the download into 8 downloads:

1. https://doi.org/10.15468/dl.475rze

2. https://doi.org/10.15468/dl.gs3vn3

3. https://doi.org/10.15468/dl.jzvhyw

4. https://doi.org/10.15468/dl.k9wkv2

5. https://doi.org/10.15468/dl.ngb8bp

6. https://doi.org/10.15468/dl.k2ynn8

7. https://doi.org/10.15468/dl.4tqz76

8. https://doi.org/10.15468/dl.z4wkd9

#### Occurrence clean up

We use the CoordinateCleaner package to clean the data. First, we removed records with known problems such as coordinates within a buffer of 2 km from country or capital centroids, near museums, herbaria, zoos, or institutes. Coordinates at sea were also deleted. We then cleaned the occurrences by removing high coordinate imprecision and Uncertainty. These are identified with values of 301, 3036, 999, or 9999 in coordinateUncertaintyInMeters.

#### Environmental variables

As ecological factors, we used the climate layers from WorldClim (Fick & Hijmans, 2017) and ENVIREM (Title & Bemmels, 2018) with a resolution of 2.5 arc min. We assessed multicollinearity using Variance Inflation Factor (VIF) using the usdm package. Variance inflation factor quantifies the severity of multicollinearity in an ordinary least squares regression analysis. We kept variables with lower VIF and high biological importance (Fig. S2). Multicollinearity was evaluated in these provinces: Arizona Mountain Forests, Sierra Madre Occidental, Sierra Madre Oriental, Transmexican Volcanic Belt, Sierra Madre del Sur, and Chiapas Highlands (Fig. S3).

Although VIF values are still high, the correlation coefficient between variables is less than 0.5 in most of the variables (Fig. S4). Besides, when applying a MDS scaling, the variables are mostly scattered (Fig. S5). The selected variables fall into three categories:

- Topographic: elevation, slope, and Topographic Roughness index. The elevation data from WorldClim was used to create the slope and Terrain Roughness Index.
- Moisture: aridity, climatic moisture, and annual precipitation. 3) Temperature: maximum temperature of the coldest month, minimum temperature of the coldest month, maximum temperature of the warmest month, minimum temperature of the warmest month.


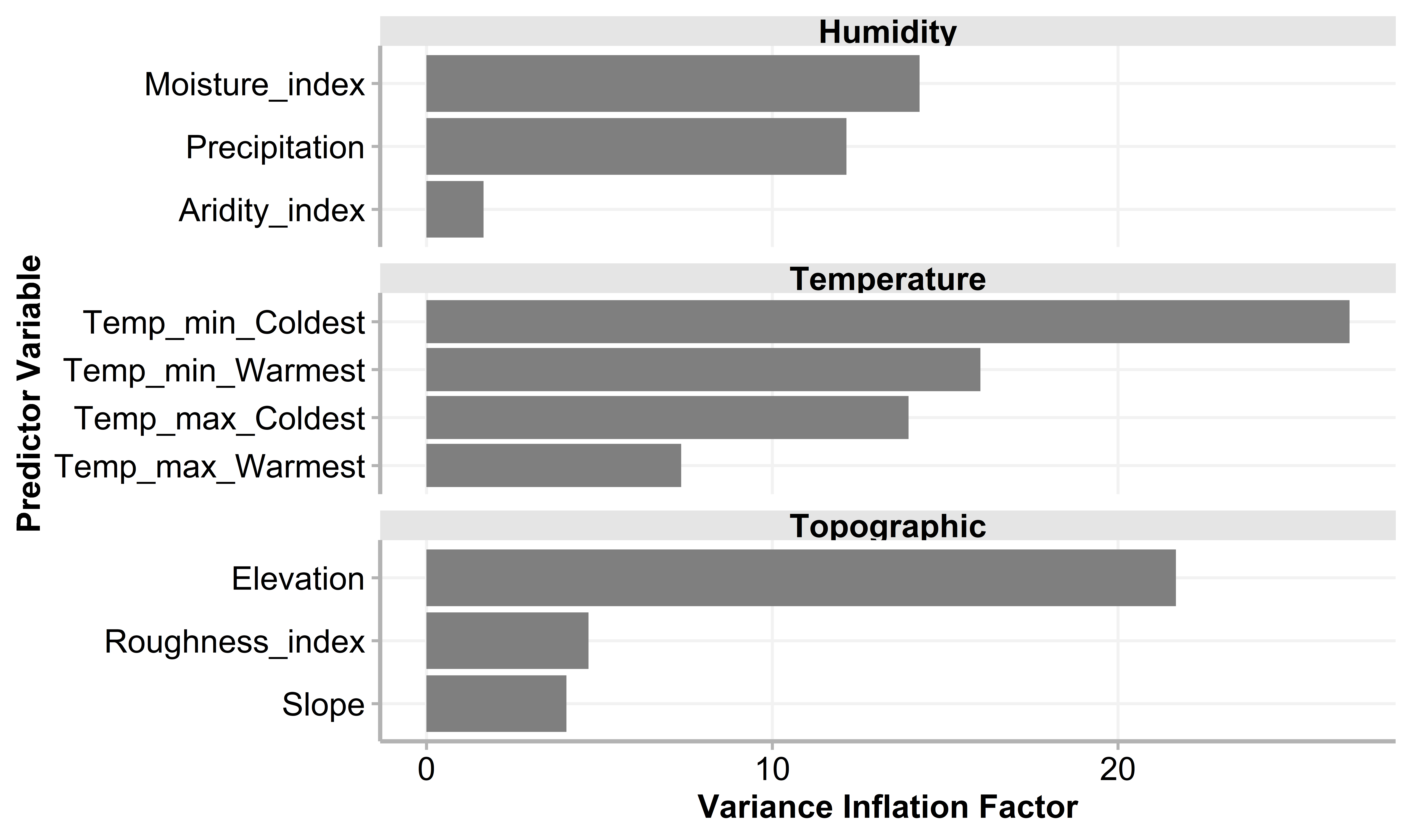


Fig. S2. Variance Inflation Factor of variables used for modelling the species distribution of Birds in the Mexican Transition Zone.


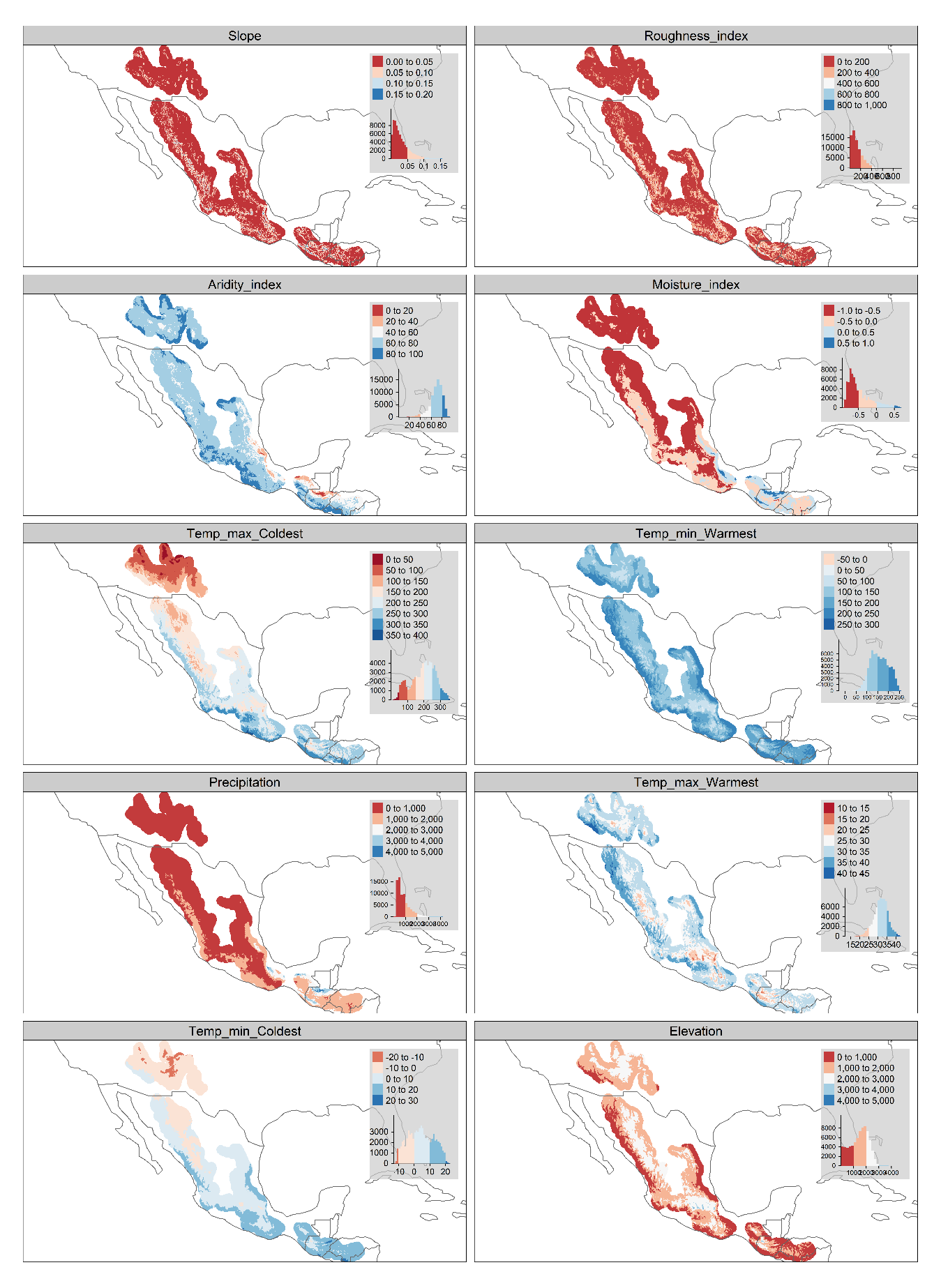


Fig. S3. Values of environmental variables used to model. The collinearity was evaluated only in the represented area.


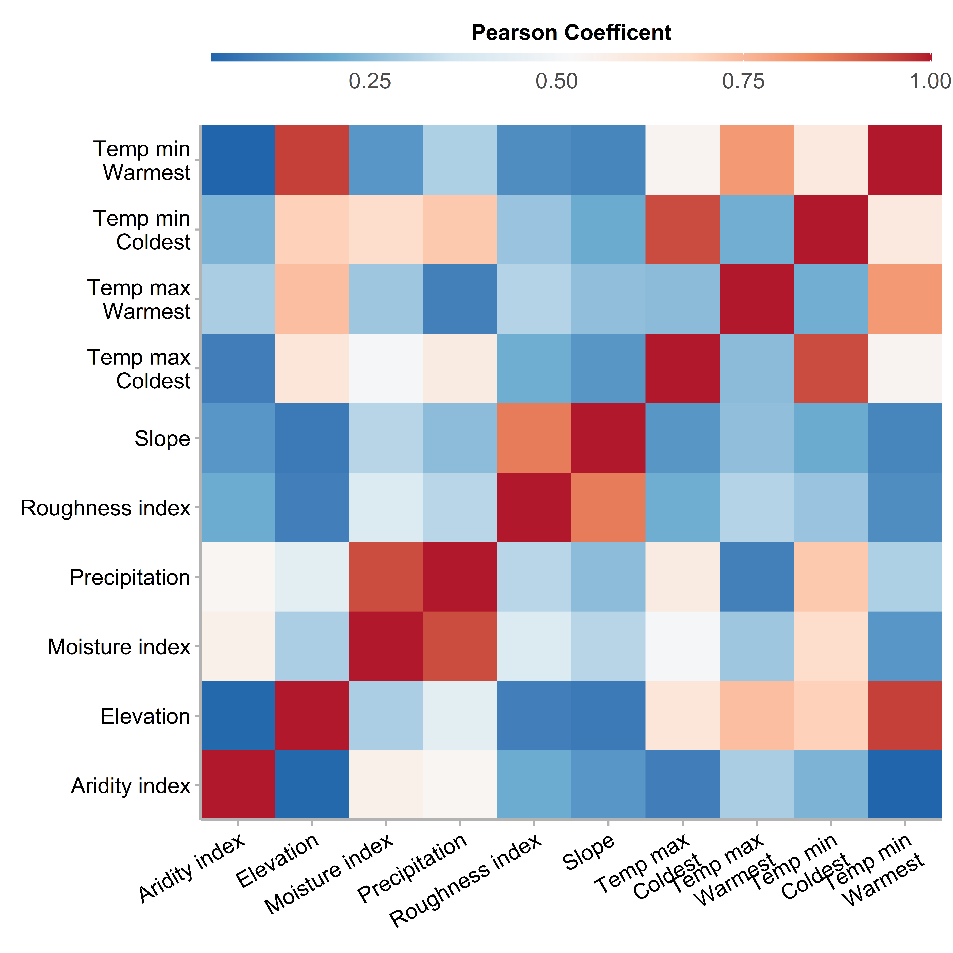


Fig. S4. Correlation between variables used for modelling the species distribution of Birds in the Mexican Transition Zone.


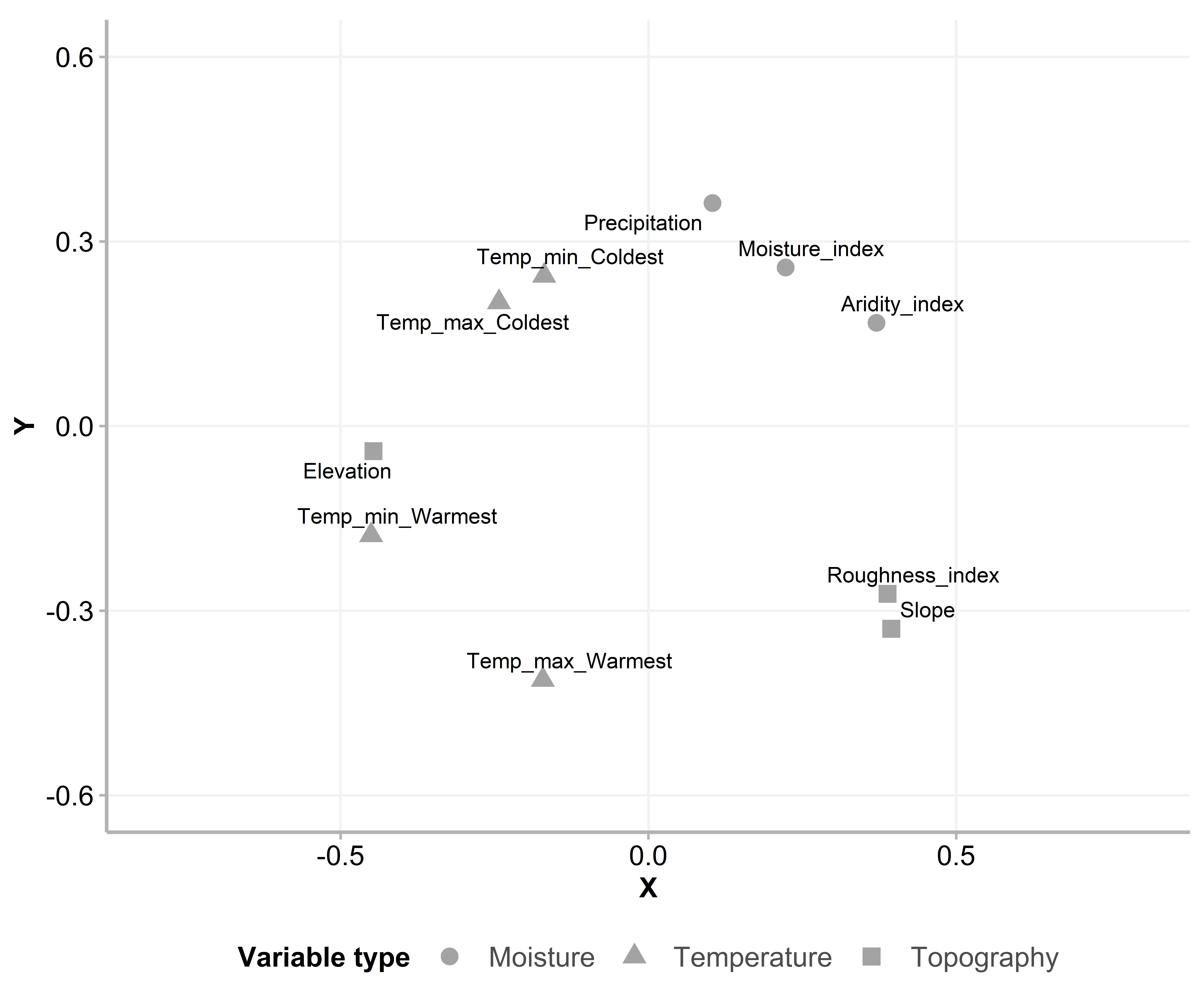


Fig. S5. Non-metric multidimensional scaling (NMDS) of variables used for modelling the species distribution of Birds in the Mexican Transition Zone.

### Species selection

We removed some groups a priori based on their biology and known distribution. This was done to focus on terrestrial birds and avoid the information noise that migratory and widespread species cause on analysis. The removed groups are: Acuatic Orders (Suliformes, Phaethontiformes, Procellariiformes, Charadriiformes, Anseriformes, Podicipediformes, Gaviiformes, Ciconiiformes, Pelecaniformes, Gruiformes, Eurypygiformes), Migratory families (Parulidae, Hirundinidae, Motacillidae), widespread families (Accipitridae, Cathartidae, Columbidae, Falconidae, Tytonidae, Strigidae), and families with less than two species in the Americas (Alaudidae, Certhiidae, Otididae, Prunellidae).

Then, we used a database of bird elevational ranges based published sources to select species distributed in Mexico and Sierra Madre de Chiapas Mountain Ranges. This includes the Mountain systems that form the Mexican Transition Zone. Additionally, we include species whose distribution mainly found in the MTZ.

#### Code

# read database
birds_db <- read.csv('data/spp_biogeo_db.csv') %>%
 distinct() %>%
 #filter by origin, seasonaliy and presence
 filter(origin == 1 | is.na(origin)) %>%
 filter(presence <6 | is.na(presence)) %>%
 filter(seasonal <= 2 | is.na(seasonal))

#Remove Orders
birds_db %>%
 # Acuatic orders
 filter(!IOCOrder %in% c(
 #Seabirds
 'Suliformes', 'Phaethontiformes', 'Procellariiformes',
 #Shorebirds
 'Charadriiformes',
 # Waterfowls, Grebes, loons
 'Anseriformes', 'Podicipediformes', 'Gaviiformes',
 # Storks Pelicans, Flamingos, Cranes
 'Ciconiiformes','Pelecaniformes',
 'Gruiformes', 'Eurypygiformes')) -> birds_db_filtered

birds_db_filtered %>%
 filter(!BLFamilyLatin %in%
 # Migratory
 c('Parulidae','Hirundinidae', 'Motacillidae',
 # Widespread
 'Accipitridae', 'Cathartidae',
 'Columbidae', 'Falconidae',
 'Tytonidae', 'Strigidae',
 #Not in America
 'Alaudidae', 'Certhiidae', 'Otididae',
 'Prunellidae') ) -> birds_db_filtered


db_chosen<- birds_db_filtered %>%
 filter(
 # Found in Mexican Mountains
 Mountain.Range %in% c('Mexico', 'Sierra Madre de Chiapas') |
 # or with its main distribution in the MTZ
 Subregion == 'Mexican.Transition.Zone')


# Species List
list_sp <- db_chosen %>%
 pull(label) %>% unique()

cat( paste( 'The number of selected species is:', length(list_sp)))

## The number of selected species is: 642

### Finding related clades

To find species or clades related to the selected species, we ran a looped search through the 1000 trees. This assures that monophyletic groups are being analysed. It first finds the parent node, then it checks if it is a single terminal or a whole clade. After applying it through the whole set of trees. It lists the number of trees in which each species is related to the species in the original list. Only those species that appear in all the trees are kept (Fig. S6). The resulting tree is showed in (Fig. S1). The consensus tree is shown for visualization only, all the analysis are run in the 1000 tree dataset.


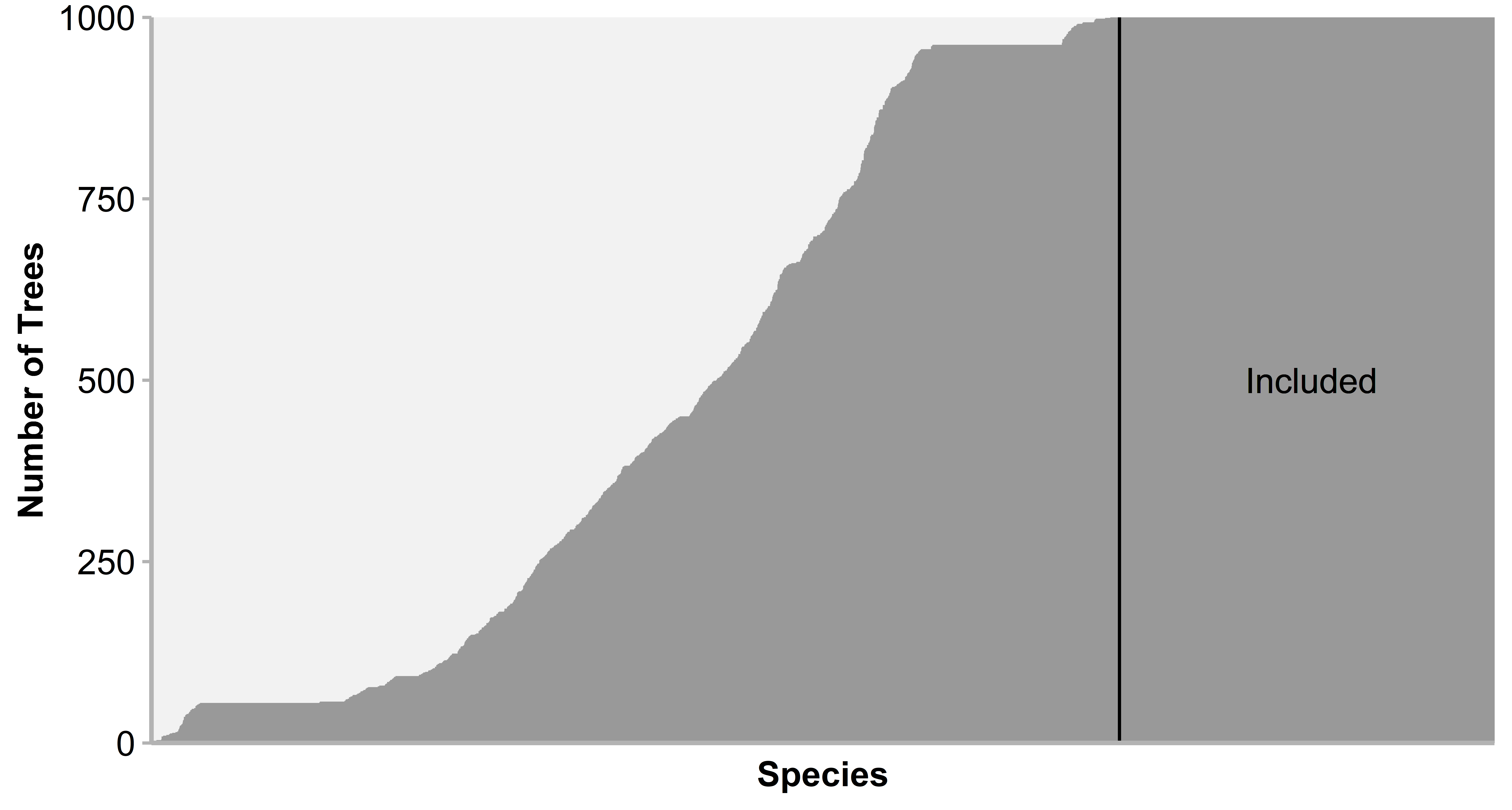


Fig. S6. Phylogenetic uncertainty in species relationship in the phylogeny of Jetz et al. (2012). We selected species that were related in all the 1000 trees.

#### Code

##### Function to find sister groups

get_sisters <- function(tr_tibble, tip_labels){
 # tr_tibble = tree tibble
 # tip_labels = vector with labels
 child_offspring <- function(x){
 # x = label
 # find Parent
 dad <- parent (tr_tibble, x) %>%
 pull(node) %>% unique()
 # Is it a single species?
 clade <- child(tr_tibble, dad)
 # or a whole clade?
 if(anyNA(clade$label) ==TRUE){
 clade <- offspring(tr_tibble, dad,
 tiponly = TRUE)}
 # get labels
 pull(clade, label)}
 # apply the function throug a list of species
 lapply(tip_labels, child_offspring) %>% unlist() %>% unique() -> sis_lis
 invisible(gc())
 return(sis_lis)
}

lapply(tree_tibble, get_sisters, tip_labels = list_sp) -> monophyletic_mx

## listar especies encontradas
unlist (monophyletic_mx) %>% tibble(label = .) %>%
 group_by(label) %>% tally() -> summary_sister_species

##### Function to select species related in all trees

library(phytools)
## load file

summary_sister_species <- read.csv( 'output/summary_sister_species.csv')
## Keep sister species found all trees
summary_sister_species %>%
 filter(n >= 1000) %>%
 pull(label) %>% c(list_sp) %>% unique()-> to_keep

length(to_keep)

# Select species to drop
to_drop <- jetz_tree[[1]]$tip.label[!jetz_tree[[1]]$tip.label %in% to_keep]
## drop tip
prunned_tree_sisters <- jetz_tree %>% drop.tip.multiPhylo(to_drop)

# save
#saveRDS(prunned_tree_sisters, file = 'output/ZTM Birds.RDS')
#writeNexus(prunned_tree_sisters, file = 'output/ZTM Birds.nex')
write.csv(summary_sister_species, 'output/summary_sister_species.csv',
 row.names = F)
birds_db_filtered %>% filter(label %in% to_keep) %>%
 write.csv('output/db_Species_to_model.csv',
 row.names = F)

### Assigning selected species to a cenocron

We selected a total of 10 orders, 47 families, 326 genus, and 808 species (Table S2). Most of them are mainly distributed in the Mexican Transition Zone and Mesoamerica (286), or have more than 80% of its distribution in the Mexican Transition Zone (246). To assign families to a certain cenocron, we used its current distribution in the biogeographic realms (Palearctic, Nearctic, Neotropical, Old World Tropics) and in the Mesoamerican region. The distribution where the species are distributed was recoded as follows:

- Northern hemisphere
  - *Nearctic*: Nearctic region, Northern Nearctic.
  - *Palearctic:* Palearctic region, Saharo-Arabian.
- Southern hemisphere
  - *Old World Tropics*: Sino-Japanese, Oriental, Ethiopian.
  - *Neotropical*: Boreal Brazilian dominion, South Brazilian dominion, Parana dominion, Pacific dominion, Chacoan dominion, Antillean subregion, Andean region, South American Transition Zone.
- Mesoamerica: Mexican Transition Zone and Mesoamerican dominion.

We counted how many species of each family were found in these three main areas. Then we assigned a cenocron to each family according to where most of its species are distributed. These are the rules we applied:

- Temperate when the family has>80% of its species are distributed in the Northern hemisphere or has more species in the Northern hemisphere than in the southern or Mesoamerica.
- Mesoamerican when the family has more species in the Mesoamerica than in the southern and northern hemisphere.
- Neotropical when any of the previous conditions did not apply.

#### Code

library(forcats)
library(scales)
# Load data
db_model <-read.csv(paste0(getwd(),
 '/output/db_Species_to_model.csv')) %>%
 group_by(IOCOrder, BLFamilyLatin, label,Mexican_TOTAL, Subregion) %>%
 tally() %>% group_by(label)%>%
 slice_max(!is.na(Mexican_TOTAL)|!is.na(Subregion),
 with_ties = F)%>%
 group_by(IOCOrder, BLFamilyLatin, label) %>%
 summarise('% Distribution in MTZ' = min(Mexican_TOTAL),
 'Mainly distributed in' = toString(unique(
 str_replace_all(Subregion, '\\.', ' ')))) %>%
 mutate(label = str_replace(label, '_', ' ')) %>%
 rename('Species' = label)

# Recoding dominions into bigger regions
db_cenocron <- db_model %>% mutate(
 dist_pattern = recode(
 `Mainly distributed in`,
 'Boreal Brazilian dominion' = 'Neotropical',
 'Mexican Transition Zone' = 'Mesoamerican',
 'Nearctic Region' = 'Nearctic',
 'South Brazilian dominion' = 'Neotropical',
 'Parana dominion' = 'Neotropical',
 'Mesoamerican dominion' = 'Mesoamerican',
 'Pacific dominion' = 'Neotropical',
 'Chacoan dominion' = 'Neotropical',
 'Antillean subregion' = 'Neotropical',
 'Northern Nearctic' = 'Nearctic',
 'Andean region' = 'Neotropical',
 'South eastern Amazonian dominion' = 'Neotropical',
 'Palearctic' = 'Palearctic',
 'Sino Japanese' = 'Old World Tropics',
 'Afrotropical' = 'Old World Tropics',
 'Oriental' = 'Old World Tropics',
 'Saharo Arabian' = 'Palearctic',
 'South American Transition Zone' = 'Neotropical')) %>%
 group_by(IOCOrder, BLFamilyLatin, dist_pattern) %>%
 tally() %>%
 filter(!dist_pattern== 'NA') %>%
 # as factors
 mutate(BLFamilyLatin = as_factor(BLFamilyLatin),
 # add order and wrap text for plotting
 dist_pattern = factor(str_wrap(dist_pattern, 10),
 levels = c('Palearctic','Nearctic',
 'Mesoamerican',
 'Neotropical',
 'Old World\nTropics')),
 # percent of species
 perc = n/sum(n))%>%
 # Recoding dominions into even bigger regions
 # Holarctic
 mutate( Holarctic = sum(perc[which(
 dist_pattern == 'Palearctic' | dist_pattern== 'Nearctic')]),
 # Holotropical
 Holotropical = sum(perc[which(
 dist_pattern == 'Old World Tropics' | dist_pattern== 'Neotropical')]),
 # Mesoamerican
 Mesoamerican = sum(perc[which(dist_pattern== 'Mesoamerican')])) %>%
 #Rules to assign to cenocron
 mutate(cenocron = ifelse(Holarctic >= .80 |
 Holarctic > (Mesoamerican + Holotropical) |
 Holarctic > Holotropical,
 'Nearctic', 'Neotropical')) %>%
 mutate(cenocron = ifelse(Mesoamerican > Holotropical &
 Mesoamerican >= Holarctic,
 'Mesoamerican', cenocron)) %>%
 # Order factors
 mutate(cenocron = factor(cenocron,
 levels = c('Nearctic', 'Mesoamerican', 'Neotropical')),
 order = recode(cenocron, Nearctic = 1,
 Mesoamerican = 2, Neotropical = 3))

dist_pattern_fam <- db_cenocron %>%
 group_by(IOCOrder, BLFamilyLatin, cenocron) %>%
 tally() %>% dplyr::select(-n)

write.csv(dist_pattern_fam, file = 'output/cenocron_families.csv', row.names = F)

### Occurrence data cleaning

Before starting the niche model algorithm, a function to prepare the records was applied to the occurrence data. It uses occurrence data per species and the Bird Life Polygons linked by the speciesKey from GBIF. This are the steps applied:

1. Identify species with few records. Species with less than 10 records were not processed.
2. Check taxonomy. Identifies species with more than one label per speciesKey and species not recognized by BirdLife (BirdLife International and Handbook of the Birds of the World, 2020).
3. Down-sample occurrences. Species with more than 10,000 records are down sampled for easier computational handling. Since the records from GBIF outnumber the ones from CONABIO’s, the records were randomly sampled to keep at least 5,000 from each source.
4. Filter out records outside the Bird Life distribution polygons.
5. Thin records, so the occurrences have more distance between them.
6. Plot the Result. It make a map with the selected and omitted records, and BirdLife Polygons (BirdLife International and Handbook of the Birds of the World, 2020).
7. Save the result as a RDS file per species, named with the species key and the label from the phylogeny.

#### Code

library(raster)
library(sf)
library(spThin)

library(dplyr)
library(stringr)
library(tmap)
library(beepr)

sf_use_s2(F)

bird_thin <- function(occ, thining, thin.par, BL){
 #benchmarking
 start_time <- Sys.time()

 # sp names and number occ
 spp_summary <- occ %>%
 group_by(speciesKey, label, valid_key) %>%
 tally(name = 'num_occ')

 message(paste('SPECIES:', spp_summary$speciesKey, spp_summary$label), '\n')

 # filename for the output
 file_name <- paste(spp_summary$valid_key, spp_summary$label, sep = '_')

 # select polygon from BL_maps and add a 5km buffer
 poly <- BL_maps %>%
 filter(speciesKey == spp_summary$speciesKey)

 # ¿Does it have enough records?
 if(spp_summary$num_occ <=10){
 cat(paste0(spp_summary$speciesKey, '\t', spp_summary$label, '\n'),
 file = paste0(wd$gis_output, 'occ_birds_mtz/not_found .txt'),
 append = TRUE)
 cat('\nERROR: ')
 message(paste(spp_summary$label, 'has not enough records\n'))
 beep(9)} else{message('\tRecords.......................OK\n')

 # ¿Is the taxonomy ok?
 if(nrow(spp_summary)>1){
 cat(paste0(spp_summary$speciesKey, '\t', spp_summary$label, '\n'),
 file = paste0(wd$gis_output,
 'occ_birds_mtz/multiple_names.txt'),
 append = TRUE)
 cat('\nERROR: ')
 message(paste(spp_summary$label, 'has taxonomical problems\n'))
 beep(9)} else {message('\tTaxonomy......................OK\n')

 # ¿is it accepted by BL?
 if(nrow(poly) == 0){
 cat(paste0(spp_summary$speciesKey, '\t', spp_summary$label, '\n'),
 file = paste0(wd$gis_output,
 'occ_birds_mtz/BL_not accepted.txt'),
 append = TRUE)
 cat('\nERROR: ')
 message(paste(spp_summary$label, 'is not accepted by Bird Life\n'))
 beep(9)} else {
 poly <- poly %>%
 st_buffer(50000) %>% tally()
 message('\tBird Life Maps................OK\n')

 # Start
 message(paste(spp_summary$label, 'started. It has',
 spp_summary$num_occ, 'records.'))

 #Remove points outside BL polygon
 st_as_sf(occ, coords =
 c('decimallongitude', 'decimallatitude')) %>%
 st_set_crs(st_crs(4326)) %>%
 mutate(lat = sf::st_coordinates(.)[,1],
 lon = sf::st_coordinates(.)[,2]) %>%
 st_transform(st_crs(poly))-> sf_occ

 message('Bird Life Filtering')

 if(BirdLife == TRUE){
 st_join(sf_occ, poly) %>%
 na.omit() %>%
 dplyr::select(-n)-> sf_occ_filtered}else{
 sf_occ-> sf_occ_filtered}

 #Downsample species with more than 5k occurrences
 if(nrow(sf_occ_filtered) > 10000){
 sf_occ_filtered %>% group_by(download_key) %>%
 sample_n(5000, replace = TRUE) %>%
 distinct() -> sf_occ_filtered

 cat(paste0(spp_summary$valid_key, '\t', spp_summary$label, '\n'),
 file = paste0(wd$gis_output, 'occ_birds_mtz/subsampled.txt'),
 append = TRUE)
 message(paste('\n', spp_summary$label,
 'was subsampled to \n',
 nrow(sf_occ_filtered)))} else{sf_occ_filtered}
 invisible(gc())
 # Message for species with less tha 10 points and abort the process
 # ¿Does it have enpugh records?
 if(nrow(sf_occ_filtered) <=10){
 cat(paste0(spp_summary$speciesKey, '\t', spp_summary$label, '\n'),
 file = paste0(wd$gis_output, 'occ_birds_mtz/not_found .txt'),
 append = TRUE)
 cat('________________________________________________\n')
 message(paste('\t', spp_summary$label, 'has not enough records\n'))
 cat('ERROR: \n')
 beep(9)} else {message('\tRecords filtered..........OK\n')
 message('\tThinning started')}

 if(thining == TRUE){
 #thining
 occ_thin <- thin(loc.data = st_drop_geometry(sf_occ_filtered),
 lat.col = "lat",
 long.col = "lon",
 spec.col = "label",
 thin.par = thin.par, reps = 1,
 max.files = 1,
 locs.thinned.list.return = TRUE,
 write.files = FALSE,
 write.log.file = FALSE,
 log.file = "spatial_thin_log.txt",
 verbose = FALSE)[[1]] %>%
 bind_cols(spp_summary) %>% dplyr::select(-num_occ)

 message('\tThinning done\n\t\tSaving RDS.')}else{

 occ_thin <- st_drop_geometry(sf_occ_filtered) %>%
 ungroup() %>%
 dplyr::select(lon, lat, speciesKey, label, valid_key) %>%
 rename('Longitude' = lon,
 'Latitude' = lat ) %>%
 as.data.frame()
 }

 #Saving output
 saveRDS(occ_thin, file = paste0(wd$gis_output, 'occ_birds_mtz/',
 file_name, '.RDS'))
 cat('\t\t\t Saved \n')
 invisible(gc())

 sf_thin <- st_as_sf(occ_thin,
 coords = c('Latitude', 'Longitude')) %>%
 st_set_crs(st_crs(4326))

 tmap_options(check.and.fix = TRUE)
 # Plot
 if(nrow(sf_occ) > 2000){
 sf_occ <- sample_n(sf_occ, 2000)} else{
 sf_occ}

 message('\t\tPlotting.')
 tm_shape(World, bb = poly) + tm_fill('grey90') +
 tm_shape(sf_occ) +
 tm_dots(col = 'grey20', alpha = 0.5,
 size = 0.05, shape = 4) +
 tm_shape(sf_thin) +
 tm_dots(col = 'tomato3', alpha = 0.5,
 size = 0.15, shape = 20) +
 tm_shape(poly) + tm_borders('grey40', lwd = 2) +
 tm_add_legend(type = 'symbol', col = c('grey30', 'tomato3'),
 size = c(0.5, 0.7), shape = c(3, 19),
 labels = c('Removed', 'Kept'),
 title = 'Occurrences') +
 tm_add_legend(type = 'line', col = 'grey40',lwd = 3,
 title = 'Range',
 labels = 'Bird Life') +
 tm_layout(legend.outside = T,
 main.title = str_replace(spp_summary$label, '_', ' '),
 title = 'Occurrence pre-process') -> map_thin
 invisible(gc())

 message('\tWriting plot. \n')

 tmap_save(map_thin,
 filename = paste0(wd$gis_output, 'occ_birds_mtz/maps/',
 file_name, '.png'),
 width = 11, height = 8, units = 'in',
 dpi = 150)

 #benchmarking and final message
 cat('\n****************** Done! ****************** \n')
 print(Sys.time() - start_time)
 cat('------------------------------------------------- \n\n')
 beep(1)
 }
 }
 }
 }

### Species Distribution Model Building

This is the function to build each species distribution model. The function is split in four steps:

1. **Choose the training area (M):** Through a spatial query to select biogeographic regions with that overlap with the Bird Life polygons. If the species is not recognized by Bird Life, then it uses occurrences to select the Biogeographic regions. The resulting polygon is dissolved, then a buffer of 10 km is applied, and the environmental layers are cut.
2. **Model building:**
   - Selection of background data,
   - Creation of SWD object for modelling through the SDMTunepackage.
   - Saves the predicted distribution raster layer.
   - Applies a threshold (*Maximum test sensitivity plus specificity*) and saves binary raster layer.
   - Saves the MaxEnt object as RData.
3. **Evaluation:** Saves a csv with three indexes included in the SDMTunepackage:
   - Area Under the Curve (AUC) using the Man-Whitney U Test formula.
   - True Skill Statistics (TSS).
   - Akaike Information Criterion (AICC) corrected for small samples size (Warren and Seifert, 2011).
4. **Plotting:** plots a map with the predicted and thresholded presence layers, occurrence data, and biogeographic regions included in the analysis. The function is then applied through a list of files containing the pre-processed occurrences and using the selected environmental variables.

#### Code

library(rJava)
library(SDMtune)
library(beepr)

model_bird <- function(key){
 start_time <- Sys.time()
 # turn off warnings
 occ_file <-list.files('D:/R_spatial/output/occ_birds_mtz',
 pattern = toString(key),
 full.names = TRUE) %>% readRDS()

 # sp names and number occ
 spp_summary <- occ_file %>%
 group_by(speciesKey, label, valid_key) %>%
 tally(name = 'num_occ')

 spp_summary <-spp_summary %>%
 mutate(file_name = paste(spp_summary$valid_key,
 spp_summary$label, sep = '_'))

 cat('\n~~~~~~~~~~~~~~~~~~~~~ Start ~~~~~~~~~~~~~~~~~~~~~~\n')
 message(paste0('\tSpecies:......................', spp_summary$label, '\n',
 '\tKey:..........................', spp_summary$speciesKey, '\n'))

 # select polygon from BL_maps and add a 5km buffer
 poly <- BL_maps %>%
 filter(speciesKey == spp_summary$speciesKey)

 if(nrow(poly)==0){
 # make M with points
 occ_file %>%
 st_as_sf(coords= c('Latitude', 'Longitude')) %>%
 st_set_crs(st_crs(biogeo)) ->points

 m <- st_join(biogeo, tally(points)) %>% filter(!is.na(n)) %>%
 group_by(ECO_NAME) %>%
 tally() %>%
 st_buffer(., 1000 , nQuadSegs = 1)

 obs <- points %>%
 st_transform(st_crs(4326)) %>%
 mutate(Latitude = sf::st_coordinates(.)[,1],
 Longitude = sf::st_coordinates(.)[,2]) %>%
 st_drop_geometry() %>%
 dplyr::select(Latitude, Longitude)

 }else{

 poly <- poly %>% tally()

 # make M with BL
 m <- st_join(biogeo, poly) %>%
 filter(!is.na(n)) %>%
 group_by(ECO_NAME) %>%
 tally() %>%
 st_buffer(., 1000 , nQuadSegs = 1)

 obs <- occ_file %>%
 dplyr::select(Latitude, Longitude)}


 if(nrow(obs) >= 10){

 m_proj <- st_transform(m, st_crs(env_raster))

 m_env <- env_raster %>%
 raster::crop(extent(m_proj)) %>%
 raster::mask(., m_proj)

 message('\tM.............................Done!\n')


 #Obtain background data
 if(ncell(m_env) > 25000){
 bg <- sampleRandom(m_env[[1]], 25000,
 xy =TRUE )[,1:2] %>%
 as.data.frame()}else{
 bg <- sampleRandom(m_env[[1]], ncell(m_env),
 xy =TRUE )[,1:2]}

 message(paste0('\tBackground....................', nrow(bg), ' cells\n'))
 #Make the model#

 # Create SWD object


 data <- prepareSWD(species = spp_summary$label,
 p = obs, a = bg,
 env = m_env )
 data <- addSamplesToBg(data)

 datasets <- trainValTest(data, test = 0.25, only_presence = TRUE)
 occ_train <- datasets[[1]]
 occ_test <- datasets[[2]]

 sf_train <- tibble(occ_train@coords) %>%
 mutate(pa = occ_train@pa) %>%
 filter(pa == 1) %>%
 st_as_sf(coords = c('X', 'Y')) %>%
 st_set_crs(st_crs(4326)) %>%
 mutate(Occurrences = 'training')

 sf_test <- occ_test@coords %>%
 mutate(pa = occ_test@pa) %>%
 filter(pa == 1) %>%
 st_as_sf(coords = c('X', 'Y')) %>%
 st_set_crs(st_crs(4326)) %>%
 mutate(Occurrences = 'testing')


 bind_rows(sf_test, sf_train) %>%
 st_transform(st_crs(BL_maps)) -> sf_occ


 maxent_model <- train(method = 'Maxent', data = occ_train, verbose = F)
 invisible(gc())
 message('\tMaxent........................Done!\n')
 save(maxent_model, file = paste0(output$model, spp_summary$file_name, '.RData'))

 message('\tSaving........................Done!\n')

 #Prediction
 predicted <- predict(maxent_model, data = m_env, type = "cloglog")

 raster::writeRaster(predicted,
 filename = paste0(output$predicted, spp_summary$file_name, '.tif'),
 overwrite=TRUE)
 message('\tPrediction....................Done!\n')

 # Threshold
 ths <- thresholds(maxent_model, test = occ_test) %>%
 filter(Threshold == 'Maximum test sensitivity plus specificity')

 rclmat <- c(0, ths$` value`, 0, ths$` value`, 1, 1) %>%
 matrix(., ncol=3, byrow=TRUE)

 thresholded <- reclassify(predicted, rclmat, include.lowest=T )

 raster::writeRaster(thresholded,
 filename = paste0(output$thresholded, spp_summary$file_name, '.tif'),
 overwrite=TRUE)
 message('\tThreshold.....................Done!\n')
 invisible(gc())
 # evaluate
 eval <- tibble(speciesKey = rep(spp_summary$speciesKey, 3),
 label = rep(spp_summary$label, 3),
 index = c('auc', 'tss', 'aicc'),
 value = c(auc(maxent_model),
 tss(maxent_model),
 aicc(maxent_model, env = m_env))) %>%
 mutate(is.valid = value>0.5 )

 write.csv(eval, file = paste0(output$eval, spp_summary$file_name, '.csv'))

 message('\tEvaluation....................Done!\n')

 # Maping

 message('\tPlotting Map..................Starting\n')

 tmap_options(show.messages= F,
 show.warnings = F,
 check.and.fix = T)

 thr_plot<- reclassify(thresholded, cbind(-Inf, 1, NA),
 right=FALSE)

 map_model <- tm_shape(World, bbox = m) +
 tm_fill('grey80') +
 tm_shape(m) +
 tm_polygons('ECO_NAME', palette = "Greys",
 lwd = 0.5, title = 'Training Area') +
 tm_shape(predicted) +
 tm_raster(style= "quantile", n= 7,
 palette= get_brewer_pal("Greys", n = 7, plot = F),
 title = 'Predicted probability') +
 tm_shape(thr_plot) +
 tm_raster(style= "quantile", n= 2,
 palette = c( 'firebrick3', '#FFFFFF'),
 alpha = 0.7,
 title = 'Distribution',
 labels = 'Predicted Presence') +
 tm_shape(World) +
 tm_borders('grey95', alpha = 0.5, lwd = 0.5) +
 tm_shape(sf_occ) + tm_dots('Occurrences',
 size = 0.05,
 palette = c('black', 'deepskyblue4')) +
 tm_scale_bar(position=c("left", "bottom")) +
 tm_layout(legend.outside = TRUE,
 main.title = str_replace(spp_summary$label, '_', ' '),
 main.title.fontface = 3,
 asp = 1,
 bg.color = 'grey95',
 frame = 'grey85')

 tmap_save(map_model,
 filename = paste0(output$maps, 'pdf/', spp_summary$file_name, '.pdf'),
 width = 11, height = 8, units = 'in',
 dpi = 300 )
 tmap_save(map_model,
 filename = paste0(output$maps, 'png/', spp_summary$file_name, '.png'),
 width = 11, height = 8, units = 'in',
 dpi = 300 )

 cat('\n**************** * Map saved * **************** \n')
 print(Sys.time() - start_time)
 cat('------------------------------------------------- \n\n')
 beep(1, expr = gc())}else{

 cat(paste0(spp_summary$speciesKey, '\t', spp_summary$label, '\n'),
 file = paste0(wd$gis_output, 'birds_sdm/not enough records.txt'),
 append = TRUE)
 cat('\nERROR: ')
 message(paste(spp_summary$label, 'has not enough records\n'))
 beep(9)}


}

## EXTENDED RESULTS

### Principal Components Analysis of environmental variables

#### Variables used

**Precipitation (pp):** Annual, Coldest Quarter, Wettest Quarter, Driest Month, Wettest Month, Seasonality, Moisture Index.

**Temperature (temp):** Mean Annual, Maximum Of The Warmest Month, Maximum Of The Coldest Month, Mean Of The Warmest Quarter, Mean Of The Coldest Quarter, Isothermallity, Seasonality, Range Annual, And Range Diurnal.

Table S2. Importance of Components of Principal Components Analysis of Mexican Transition Zone environmental variables.

|  | PC1 | PC2 | PC3 | PC4 | PC5 | PC6 | PC7 | PC8 |
| --- | --- | --- | --- | --- | --- | --- | --- | --- |
| Standard deviation | 3.016 | 1.926 | 1.249 | 0.850 | 0.739 | 0.462 | 0.260 | 0.201 |
| Proportion of Variance | 56.9% | 23.2% | 9.7% | 4.5% | 3.4% | 1.3% | 0.4% | 0.3% |
| Cumulative Proportion | 56.9% | 80.0% | 89.8% | 94.3% | 97.7% | 99.1% | 99.5% | 99.7% |


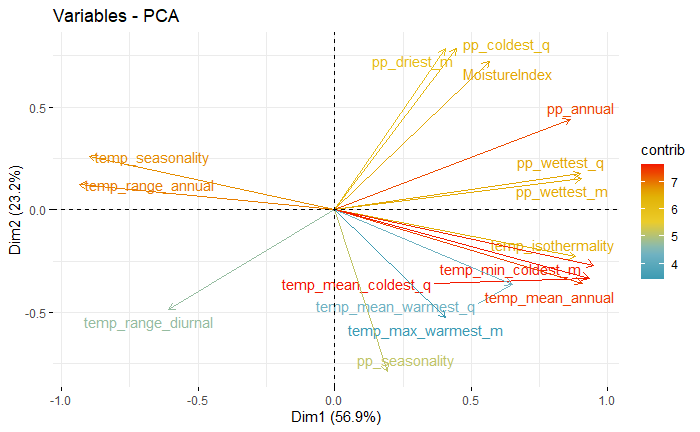


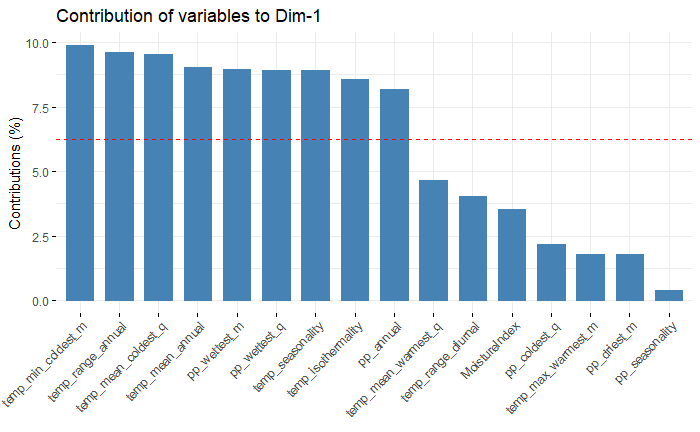


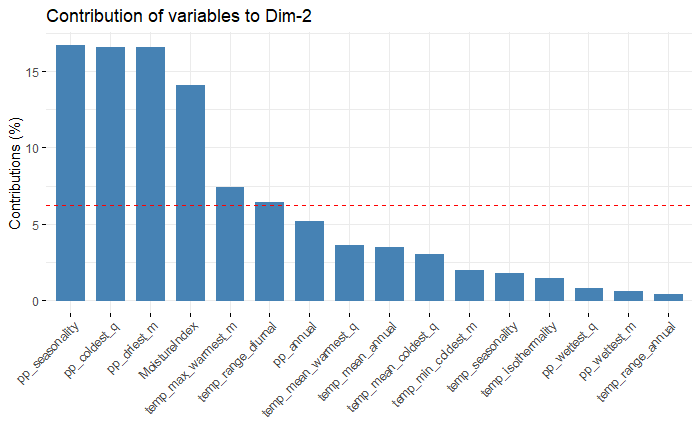

Supplement: Supplemental Information 1 [file peerj-12-16664-s001.docx]
