# Supplementary material for: Niche conservatism and convergence in birds of three cenocrons in the Mexican Transition Zone
Source: PeerJ. 2024 Jan 2;12:e16664. doi: 10.7717/peerj.16664 (PMC10768671; doi:10.7717/peerj.16664)
Supplement: Supplemental Information 3 — Species in alphabetical order, grey indicates that one index is ¡ 0.5. [file peerj-12-16664-s003.docx]

S3: MTZ's birds SDM Evaluation

Species in alphabetical order, grey indicates that the mean pAUC ratio is <1.1

| **Species** | **Num. points** | **fc** | **reg** | **iter** | **AICc** | **Δ AICc** | **Mean AUC** | **Mean pAUC ratio at 5%** | **P value** | **Ommission rate at 5%** | **Used variables** | **Threshold** |
| --- | --- | --- | --- | --- | --- | --- | --- | --- | --- | --- | --- | --- |
| *Abeillia abeillei* | 76 | lq | 1.0 | 500 | 1140.160 | 0.913 | 0.877 | 1.461 | 0.000 | 0.000 | Aridity_index, Temp_max_Coldest, Temp_min_Warmest | 0.552 |
| *Aeronautes saxatalis* | 1872 | lqp | 0.9 | 500 | 33320.970 | 13.254 | 0.801 | 1.301 | 0.000 | 0.074 | Roughness_index, Aridity_index, Temp_max_Coldest, Elevation | 0.420 |
| *Agelaioides badius* | 1628 | lqp | 1.1 | 500 | 29658.984 | 14.805 | 0.846 | 1.308 | 0.000 | 0.063 | Moisture_index, Temp_max_Coldest, Elevation | 0.395 |
| *Agelaius assimilis* | 14 | lq | 1.0 | 500 | 150.413 | 0.436 | 0.713 | 1.770 | 0.000 | 0.250 | pp_coldest_q, temp_mean_coldest_q, temp_range_diurnal, temp_seasonality | 0.532 |
| *Agelaius humeralis* | 100 | lp | 1.0 | 500 | 977.637 | 0.257 | 0.673 | 1.170 | 0.000 | 0.167 | Aridity_index, Temp_max_Coldest, Temp_min_Warmest, Precipitation | 0.433 |
| *Agelaius phoeniceus* | 2955 | lqp | 0.9 | 500 | 56528.442 | 49.112 | 0.808 | 1.365 | 0.000 | 0.046 | Precipitation, Temp_max_Warmest, Elevation | 0.482 |
| *Agelaius tricolor* | 588 | lqp | 0.8 | 500 | 8395.626 | 0.000 | 0.956 | 1.684 | 0.000 | 0.051 | Roughness_index, Temp_max_Coldest, Precipitation, Elevation | 0.291 |
| *Agelaius xanthomus* | 13 | lqph | 1.0 | 500 | NA | NA | 0.896 | 1.808 | 0.000 | 1.000 | Slope, Roughness_index, Aridity_index, Moisture_index, Temp_max_Coldest, Temp_min_Warmest, Precipitation, Temp_max_Warmest, Temp_min_Coldest, Elevation | 0.382 |
| *Agelasticus cyanopus* | 873 | lqp | 0.8 | 500 | 15292.485 | 0.413 | 0.884 | 1.431 | 0.000 | 0.052 | Aridity_index, Temp_max_Warmest, Temp_min_Coldest, Elevation | 0.230 |
| *Agelasticus thilius* | 1167 | lqp | 0.8 | 500 | 19500.920 | 0.000 | 0.850 | 1.385 | 0.000 | 0.048 | Moisture_index, Temp_max_Coldest, Elevation | 0.297 |
| *Agelasticus xanthophthalmus* | 33 | lq | 0.8 | 500 | 537.998 | 0.000 | 0.745 | 1.357 | 0.000 | 0.125 | pp_coldest_q, temp_mean_coldest_q, temp_range_diurnal, temp_seasonality | 0.471 |
| *Aimophila aestivalis* | 402 | lqp | 0.8 | 500 | 6399.043 | 0.000 | 0.803 | 1.316 | 0.000 | 0.031 | Moisture_index, Temp_max_Coldest | 0.428 |
| *Aimophila botterii* | 357 | qp | 0.8 | 500 | 6105.838 | 0.000 | 0.691 | 1.147 | 0.000 | 0.047 | Slope, Temp_min_Warmest, Precipitation | 0.582 |
| *Aimophila carpalis* | 237 | lqp | 0.8 | 500 | 3313.873 | 0.000 | 0.889 | 1.592 | 0.000 | 0.036 | Roughness_index, Precipitation, Elevation | 0.377 |
| *Aimophila cassinii* | 1164 | lq | 0.8 | 500 | 20418.908 | 0.641 | 0.898 | 1.603 | 0.000 | 0.038 | Roughness_index, Moisture_index, Temp_max_Coldest | 0.329 |
| *Aimophila humeralis* | 91 | lq | 0.8 | 500 | 1351.209 | 0.000 | 0.647 | 1.166 | 0.000 | 0.091 | Roughness_index, Aridity_index, Temp_min_Coldest | 0.748 |
| *Aimophila mystacalis* | 79 | lq | 1.1 | 500 | 1011.453 | 0.544 | 0.920 | 1.783 | 0.000 | 0.050 | Aridity_index, Temp_max_Coldest, Precipitation, Elevation | 0.310 |
| *Aimophila notosticta* | 24 | lq | 0.8 | 500 | 338.209 | 2.516 | 0.849 | 1.602 | 0.000 | 0.167 | pp_coldest_q, temp_mean_coldest_q, temp_range_diurnal, temp_seasonality | 0.493 |
| *Aimophila quinquestriata* | 81 | lqp | 0.8 | 500 | 1209.670 | 0.000 | 0.880 | 1.554 | 0.000 | 0.100 | Slope, Moisture_index, Temp_max_Coldest, Elevation | 0.391 |
| *Aimophila rufescens* | 356 | lqp | 1.0 | 500 | 5800.461 | 1.574 | 0.810 | 1.270 | 0.000 | 0.057 | Roughness_index, Moisture_index, Temp_min_Warmest | 0.381 |
| *Aimophila ruficauda* | 326 | lq | 0.8 | 500 | 4132.564 | 0.000 | 0.846 | 1.460 | 0.000 | 0.015 | Roughness_index, Aridity_index, Temp_min_Warmest, Precipitation | 0.393 |
| *Aimophila ruficeps* | 1210 | lqp | 0.8 | 500 | 20995.316 | 0.000 | 0.902 | 1.539 | 0.000 | 0.065 | Roughness_index, Moisture_index, Temp_max_Coldest | 0.337 |
| *Aimophila sumichrasti* | 39 | qp | 0.8 | 500 | 468.975 | 4.405 | 0.685 | 1.336 | 0.000 | 0.375 | pp_coldest_q, temp_mean_coldest_q, temp_range_diurnal, temp_seasonality | 0.435 |
| *Amaurospiza concolor* | 12 | lqp | 1.1 | 500 | 186.134 | 0.637 | 0.459 | 1.209 | 0.000 | 0.667 | pp_coldest_q, temp_mean_coldest_q, temp_range_diurnal, temp_seasonality | 0.657 |
| *Amaurospiza moesta* | 153 | lqp | 0.8 | 500 | 2605.561 | 0.000 | 0.967 | 1.772 | 0.000 | 0.000 | Roughness_index, Aridity_index, Temp_min_Coldest | 0.282 |
| *Amazilia amabilis* | 375 | lqp | 1.0 | 500 | 5184.613 | 1.409 | 0.800 | 1.351 | 0.000 | 0.080 | Temp_max_Warmest, Temp_min_Coldest | 0.392 |
| *Amazilia beryllina* | 820 | lqp | 1.1 | 500 | 12868.863 | 0.669 | 0.748 | 1.163 | 0.000 | 0.058 | Slope, Aridity_index, Moisture_index, Temp_min_Warmest | 0.461 |
| *Amazilia candida* | 396 | lq | 1.0 | 500 | 5841.475 | 1.755 | 0.762 | 1.252 | 0.000 | 0.032 | Roughness_index, Aridity_index, Temp_min_Coldest | 0.472 |
| *Amazilia cyanocephala* | 368 | lqp | 1.2 | 500 | 5621.537 | 3.139 | 0.802 | 1.281 | 0.000 | 0.033 | Moisture_index, Temp_min_Warmest | 0.514 |
| *Amazilia cyanura* | 83 | lqph | 1.0 | 500 | NA | NA | 0.910 | 1.436 | 0.000 | 0.031 | Slope, Roughness_index, Aridity_index, Moisture_index, Temp_max_Coldest, Temp_min_Warmest, Precipitation, Temp_max_Warmest, Temp_min_Coldest, Elevation | 0.286 |
| *Amazilia luciae* | 23 | lq | 1.2 | 500 | 289.093 | 0.970 | 0.888 | 1.694 | 0.000 | 0.000 | pp_coldest_q, temp_mean_coldest_q, temp_range_diurnal, temp_seasonality | 0.431 |
| *Amazilia rutila* | 385 | lqp | 0.8 | 500 | 4631.723 | 0.000 | 0.772 | 1.281 | 0.000 | 0.042 | Roughness_index, Aridity_index, Moisture_index, Temp_max_Coldest, Temp_max_Warmest | 0.431 |
| *Amazilia saucerrottei* | 102 | qp | 0.9 | 500 | 1531.805 | 2.605 | 0.710 | 1.116 | 0.000 | 0.333 | Aridity_index, Precipitation, Elevation | 0.514 |
| *Amazilia violiceps* | 468 | lqp | 1.2 | 500 | 7067.565 | 3.297 | 0.792 | 1.209 | 0.000 | 0.093 | Slope, Moisture_index, Temp_min_Warmest, Temp_min_Coldest | 0.417 |
| *Amazilia viridifrons* | 78 | lqp | 0.8 | 500 | 1138.504 | 0.000 | 0.808 | 1.360 | 0.000 | 0.179 | Moisture_index, Temp_max_Coldest, Temp_min_Warmest | 0.448 |
| *Amazilia yucatanensis* | 266 | lqp | 1.2 | 500 | 3838.822 | 0.727 | 0.740 | 1.290 | 0.000 | 0.067 | Moisture_index, Temp_min_Warmest | 0.455 |
| *Amazona albifrons* | 310 | lqp | 1.0 | 500 | 4178.735 | 0.670 | 0.740 | 1.291 | 0.000 | 0.062 | Moisture_index, Elevation | 0.381 |
| *Amazona autumnalis* | 396 | lqp | 0.9 | 500 | 5948.054 | 5.305 | 0.767 | 1.136 | 0.000 | 0.096 | Moisture_index, Temp_max_Coldest, Temp_max_Warmest | 0.483 |
| *Amazona farinosa* | 824 | lq | 0.8 | 500 | 15321.324 | 0.000 | 0.721 | 1.097 | 0.000 | 0.086 | Roughness_index, Moisture_index, Temp_max_Coldest | 0.521 |
| *Amazona finschi* | 115 | lqp | 1.0 | 500 | 1491.902 | 0.841 | 0.862 | 1.465 | 0.000 | 0.000 | Slope, Aridity_index, Moisture_index, Temp_min_Warmest | 0.561 |
| *Amazona ochrocephala* | 1183 | lp | 0.8 | 500 | 21713.561 | 2.876 | 0.721 | 1.168 | 0.000 | 0.060 | Slope, Moisture_index, Temp_max_Warmest, Temp_min_Coldest | 0.655 |
| *Amazona oratrix* | 106 | lp | 0.9 | 500 | 1552.594 | 0.849 | 0.616 | 1.074 | 0.000 | 0.167 | Aridity_index, Temp_max_Coldest, Temp_min_Warmest, Precipitation | 0.537 |
| *Amazona viridigenalis* | 74 | lq | 0.8 | 500 | 999.674 | 0.000 | 0.850 | 1.383 | 0.000 | 0.111 | Temp_min_Warmest, Precipitation | 0.537 |
| *Amblycercus holosericeus* | 513 | lqp | 0.8 | 500 | 8319.505 | 0.000 | 0.767 | 1.121 | 0.000 | 0.103 | Roughness_index, Moisture_index, Temp_max_Coldest | 0.418 |
| *Amblyramphus holosericeus* | 666 | lqp | 1.0 | 500 | 10891.537 | 9.042 | 0.925 | 1.584 | 0.000 | 0.025 | Aridity_index, Temp_max_Warmest, Temp_min_Coldest, Elevation | 0.318 |
| *Ammodramus bairdii* | 22 | lqp | 0.9 | 500 | 382.408 | 0.000 | 0.817 | 1.511 | 0.000 | 0.000 | Aridity_index, Temp_min_Coldest, Elevation | 0.610 |
| *Ammodramus savannarum* | 2030 | lqp | 0.8 | 500 | 39111.669 | 0.000 | 0.765 | 1.280 | 0.000 | 0.040 | Moisture_index, Temp_max_Warmest, Elevation | 0.537 |
| *Amphispiza bilineata* | 1734 | lqp | 1.0 | 500 | 30333.407 | 20.064 | 0.843 | 1.417 | 0.000 | 0.071 | Roughness_index, Moisture_index, Temp_max_Coldest | 0.357 |
| *Anabacerthia variegaticeps* | 152 | lqp | 0.8 | 500 | 2326.182 | 0.000 | 0.847 | 1.193 | 0.000 | 0.053 | Roughness_index, Moisture_index, Temp_max_Coldest | 0.366 |
| *Anthracothorax prevostii* | 315 | lqp | 1.1 | 500 | 4399.261 | 0.453 | 0.770 | 1.215 | 0.000 | 0.000 | Slope, Aridity_index, Moisture_index | 0.602 |
| *Anthracothorax veraguensis* | 37 | lp | 0.8 | 500 | 319.460 | 0.166 | 0.509 | 1.190 | 0.000 | 0.500 | pp_coldest_q, temp_mean_coldest_q, temp_range_diurnal, temp_seasonality | 0.472 |
| *Aphelocoma californica* | 923 | lqp | 0.8 | 500 | 14160.373 | 0.000 | 0.918 | 1.539 | 0.000 | 0.062 | Roughness_index, Aridity_index, Temp_min_Coldest, Elevation | 0.296 |
| *Aphelocoma ultramarina* | 83 | qp | 1.0 | 500 | 1180.727 | 0.322 | 0.933 | 1.588 | 0.000 | 0.190 | Roughness_index, Aridity_index, Moisture_index, Temp_min_Warmest | 0.315 |
| *Aphelocoma unicolor* | 59 | lq | 0.8 | 500 | 853.006 | 0.000 | 0.906 | 1.527 | 0.000 | 0.000 | Slope, Moisture_index, Temp_max_Coldest, Temp_max_Warmest | 0.415 |
| *Ara macao* | 908 | lp | 0.9 | 500 | 17244.944 | 0.000 | 0.712 | 1.226 | 0.000 | 0.054 | Roughness_index, Aridity_index, Temp_max_Warmest, Temp_min_Coldest | 0.479 |
| *Ara militaris* | 251 | lqp | 0.9 | 500 | 4355.383 | 0.000 | 0.864 | 1.311 | 0.000 | 0.050 | Roughness_index, Moisture_index, Temp_max_Warmest | 0.426 |
| *Aratinga canicularis* | 336 | lqp | 0.8 | 500 | 4110.419 | 2.990 | 0.821 | 1.417 | 0.000 | 0.076 | Roughness_index, Aridity_index, Temp_max_Coldest | 0.308 |
| *Aratinga finschi* | 205 | lqp | 0.8 | 500 | 2418.873 | 0.489 | 0.821 | 1.309 | 0.000 | 0.163 | Temp_max_Coldest, Temp_min_Warmest, Precipitation | 0.508 |
| *Aratinga nana* | 273 | lq | 0.8 | 500 | 3542.374 | 0.000 | 0.744 | 1.261 | 0.000 | 0.089 | Aridity_index, Temp_max_Warmest, Elevation | 0.399 |
| *Archilochus alexandri* | 1410 | lq | 0.8 | 500 | 25984.246 | 41.297 | 0.743 | 1.251 | 0.000 | 0.061 | Roughness_index, Temp_max_Coldest, Precipitation | 0.412 |
| *Archilochus colubris* | 1912 | lqp | 1.0 | 500 | 34407.184 | 32.542 | 0.819 | 1.404 | 0.000 | 0.040 | Aridity_index, Temp_max_Warmest, Elevation | 0.468 |
| *Arremon aurantiirostris* | 406 | lqp | 1.0 | 500 | 6349.577 | 3.322 | 0.676 | 1.120 | 0.000 | 0.086 | Aridity_index, Temp_min_Warmest | 0.632 |
| *Arremon brunneinucha* | 547 | lqp | 0.9 | 500 | 9093.080 | 0.543 | 0.927 | 1.647 | 0.000 | 0.015 | Moisture_index, Elevation | 0.306 |
| *Arremon virenticeps* | 115 | qp | 0.8 | 500 | 1510.144 | 2.596 | 0.920 | 1.453 | 0.000 | 0.071 | Roughness_index, Moisture_index, Temp_max_Warmest, Temp_min_Coldest | 0.178 |
| *Arremonops chloronotus* | 174 | lqp | 0.8 | 500 | 2308.462 | 0.000 | 0.766 | 1.215 | 0.000 | 0.079 | Roughness_index, Aridity_index, Moisture_index, Temp_min_Coldest | 0.536 |
| *Arremonops conirostris* | 527 | lqp | 1.2 | 500 | 8747.950 | 13.122 | 0.731 | 1.099 | 0.000 | 0.090 | Aridity_index, Temp_max_Warmest | 0.638 |
| *Arremonops rufivirgatus* | 442 | lqp | 1.1 | 500 | 6658.631 | 2.301 | 0.820 | 1.351 | 0.000 | 0.101 | Moisture_index, Temp_max_Coldest, Elevation | 0.335 |
| *Aspatha gularis* | 92 | lq | 0.8 | 500 | 1213.529 | 0.000 | 0.911 | 1.432 | 0.000 | 0.087 | Moisture_index, Temp_max_Coldest, Elevation | 0.275 |
| *Atlapetes albinucha* | 279 | lqp | 1.0 | 500 | 4263.706 | 0.440 | 0.872 | 1.366 | 0.000 | 0.043 | Moisture_index, Elevation | 0.293 |
| *Atlapetes pileatus* | 210 | lqp | 0.8 | 500 | 3141.670 | 0.000 | 0.861 | 1.331 | 0.000 | 0.115 | Roughness_index, Temp_max_Warmest, Temp_min_Coldest | 0.443 |
| *Atthis ellioti* | 50 | qp | 1.0 | 500 | 606.081 | 0.136 | 0.961 | 1.769 | 0.000 | 0.000 | Roughness_index, Moisture_index, Temp_max_Coldest, Temp_max_Warmest | 0.246 |
| *Atthis heloisa* | 128 | qp | 1.0 | 500 | 1687.066 | 2.396 | 0.896 | 1.471 | 0.000 | 0.064 | Roughness_index, Aridity_index, Temp_max_Warmest, Temp_min_Coldest | 0.269 |
| *Attila spadiceus* | 942 | lqp | 1.2 | 500 | 17064.810 | 6.285 | 0.715 | 1.069 | 0.001 | 0.051 | Roughness_index, Moisture_index, Temp_max_Coldest | 0.550 |
| *Aulacorhynchus prasinus* | 235 | lqp | 1.0 | 500 | 3860.209 | 1.667 | 0.895 | 1.380 | 0.000 | 0.051 | Moisture_index, Temp_max_Coldest | 0.308 |
| *Auriparus flaviceps* | 1250 | lq | 0.8 | 500 | 20949.177 | 1.764 | 0.893 | 1.600 | 0.000 | 0.050 | Moisture_index, Temp_max_Coldest, Elevation | 0.295 |
| *Automolus ochrolaemus* | 712 | lq | 0.8 | 500 | 13462.635 | 7.628 | 0.758 | 1.175 | 0.000 | 0.028 | Roughness_index, Moisture_index, Temp_max_Coldest | 0.553 |
| *Automolus rubiginosus* | 248 | lq | 0.8 | 500 | 4426.706 | 0.000 | 0.892 | 1.421 | 0.000 | 0.016 | Roughness_index, Moisture_index, Temp_max_Warmest | 0.390 |
| *Baeolophus atricristatus* | 470 | lqp | 0.9 | 500 | 7474.227 | 3.334 | 0.898 | 1.531 | 0.000 | 0.070 | Roughness_index, Moisture_index, Temp_max_Coldest, Temp_min_Warmest | 0.275 |
| *Baeolophus bicolor* | 1500 | lqp | 0.8 | 500 | 26572.772 | 0.000 | 0.788 | 1.312 | 0.000 | 0.044 | Roughness_index, Moisture_index, Temp_max_Coldest, Elevation | 0.382 |
| *Baeolophus inornatus* | 551 | lqp | 1.1 | 500 | 7817.047 | 14.600 | 0.962 | 1.808 | 0.000 | 0.053 | Roughness_index, Temp_max_Coldest, Precipitation, Elevation | 0.255 |
| *Baeolophus ridgwayi* | 820 | lqp | 0.8 | 500 | 14059.629 | 0.000 | 0.888 | 1.528 | 0.000 | 0.034 | Roughness_index, Temp_max_Coldest, Precipitation, Elevation | 0.349 |
| *Baeolophus wollweberi* | 440 | lqp | 1.1 | 500 | 7237.403 | 4.024 | 0.805 | 1.237 | 0.000 | 0.127 | Roughness_index, Moisture_index, Temp_max_Coldest, Temp_min_Warmest | 0.462 |
| *Baryphthengus martii* | 444 | lqp | 0.8 | 500 | 7780.252 | 0.000 | 0.832 | 1.289 | 0.000 | 0.112 | Roughness_index, Aridity_index, Temp_max_Warmest, Temp_min_Coldest | 0.436 |
| *Bolborhynchus lineola* | 223 | lqp | 0.9 | 500 | 3641.626 | 0.180 | 0.905 | 1.573 | 0.000 | 0.036 | Moisture_index, Temp_min_Warmest | 0.257 |
| *Brotogeris jugularis* | 450 | lqp | 0.8 | 500 | 6531.247 | 0.000 | 0.655 | 1.119 | 0.000 | 0.072 | Moisture_index, Temp_max_Coldest | 0.520 |
| *Brotogeris pyrrhoptera* | 59 | lq | 1.2 | 500 | 677.788 | 2.256 | 0.826 | 1.463 | 0.000 | 0.231 | Precipitation, Temp_min_Coldest | 0.423 |
| *Cacicus melanicterus* | 293 | lqp | 0.8 | 500 | 3507.164 | 0.000 | 0.857 | 1.457 | 0.000 | 0.140 | Slope, Aridity_index, Temp_max_Coldest | 0.347 |
| *Cacicus microrhynchus* | 202 | lq | 0.9 | 500 | 3133.860 | 0.449 | 0.854 | 1.456 | 0.000 | 0.106 | Moisture_index, Temp_max_Coldest | 0.266 |
| *Calamospiza melanocorys* | 1258 | lqp | 0.8 | 500 | 22430.489 | 0.000 | 0.869 | 1.469 | 0.000 | 0.045 | Roughness_index, Aridity_index, Temp_max_Warmest | 0.434 |
| *Calcarius mccownii* | 424 | qp | 0.8 | 500 | 7236.213 | 17.308 | 0.908 | 1.582 | 0.000 | 0.038 | Moisture_index, Temp_min_Warmest, Temp_min_Coldest | 0.297 |
| *Callipepla californica* | 1408 | lq | 0.8 | 500 | 22432.018 | 30.442 | 0.861 | 1.426 | 0.000 | 0.055 | Roughness_index, Aridity_index, Elevation | 0.338 |
| *Callipepla douglasii* | 159 | lp | 1.0 | 500 | 2064.248 | 1.084 | 0.931 | 1.692 | 0.000 | 0.056 | Slope, Moisture_index, Temp_min_Warmest, Temp_min_Coldest | 0.229 |
| *Callipepla gambelii* | 710 | lqp | 0.8 | 500 | 11839.456 | 0.000 | 0.895 | 1.536 | 0.000 | 0.029 | Roughness_index, Precipitation, Temp_max_Warmest | 0.310 |
| *Callipepla squamata* | 998 | lqp | 0.8 | 500 | 17511.993 | 0.000 | 0.862 | 1.477 | 0.000 | 0.040 | Roughness_index, Moisture_index, Temp_max_Coldest, Elevation | 0.429 |
| *Calocitta colliei* | 221 | lqp | 0.8 | 500 | 2993.667 | 0.000 | 0.900 | 1.569 | 0.000 | 0.100 | Roughness_index, Moisture_index, Temp_max_Coldest, Temp_min_Warmest | 0.321 |
| *Calocitta formosa* | 287 | qp | 0.9 | 500 | 3494.214 | 0.296 | 0.781 | 1.276 | 0.000 | 0.107 | Roughness_index, Moisture_index, Temp_max_Coldest, Temp_max_Warmest | 0.459 |
| *Calothorax lucifer* | 211 | lqp | 0.8 | 500 | 3399.173 | 0.000 | 0.763 | 1.290 | 0.000 | 0.118 | Slope, Precipitation, Temp_min_Coldest | 0.435 |
| *Calothorax pulcher* | 31 | qp | 0.8 | 500 | 437.532 | 1.497 | 0.802 | 1.439 | 0.000 | 0.125 | pp_coldest_q, temp_mean_coldest_q, temp_range_diurnal, temp_seasonality | 0.381 |
| *Calypte anna* | 881 | lqp | 0.8 | 500 | 13240.000 | 0.000 | 0.932 | 1.645 | 0.000 | 0.045 | Roughness_index, Temp_max_Coldest, Precipitation, Elevation | 0.285 |
| *Calypte costae* | 782 | lqp | 0.8 | 500 | 11636.480 | 0.000 | 0.923 | 1.670 | 0.000 | 0.017 | Roughness_index, Temp_max_Coldest, Precipitation, Elevation | 0.314 |
| *Campephilus guatemalensis* | 407 | lqp | 1.1 | 500 | 5467.990 | 0.512 | 0.794 | 1.407 | 0.000 | 0.061 | Precipitation, Elevation | 0.306 |
| *Campephilus imperialis* | 53 | qp | 0.8 | 500 | 872.517 | 2.043 | 0.827 | 1.474 | 0.000 | 0.000 | pp_coldest_q, temp_mean_coldest_q, temp_range_diurnal, temp_seasonality | 0.615 |
| *Camptostoma imberbe* | 776 | lq | 1.1 | 500 | 12344.513 | 0.076 | 0.670 | 1.149 | 0.005 | 0.040 | Moisture_index, Temp_min_Warmest | 0.499 |
| *Camptostoma obsoletum* | 1890 | lqp | 0.8 | 500 | 35430.784 | 0.000 | 0.707 | 1.070 | 0.000 | 0.088 | Moisture_index, Temp_max_Coldest, Elevation | 0.616 |
| *Campylopterus curvipennis* | 157 | lqp | 1.1 | 500 | 2434.745 | 0.793 | 0.770 | 1.306 | 0.000 | 0.125 | Moisture_index, Temp_min_Warmest | 0.419 |
| *Campylopterus hemileucurus* | 218 | lq | 0.8 | 500 | 3131.477 | 0.000 | 0.851 | 1.315 | 0.000 | 0.058 | Moisture_index, Temp_min_Warmest | 0.373 |
| *Campylopterus rufus* | 49 | lq | 1.0 | 500 | 177.988 | 1.037 | 0.913 | 1.491 | 0.000 | 0.150 | pp_coldest_q, temp_mean_coldest_q, temp_range_diurnal, temp_seasonality | 0.541 |
| *Campylorhynchus albobrunneus* | 60 | lq | 1.2 | 500 | 808.075 | 0.489 | 0.646 | 1.282 | 0.000 | 0.071 | Moisture_index, Temp_min_Warmest | 0.510 |
| *Campylorhynchus brunneicapillus* | 1351 | lqp | 1.0 | 500 | 22987.129 | 4.250 | 0.882 | 1.601 | 0.000 | 0.040 | Roughness_index, Moisture_index, Temp_min_Coldest | 0.320 |
| *Campylorhynchus chiapensis* | 32 | lq | 0.8 | 500 | 277.189 | 0.000 | 0.951 | 1.840 | 0.000 | 0.000 | pp_coldest_q, temp_mean_coldest_q, temp_range_diurnal, temp_seasonality | 0.510 |
| *Campylorhynchus griseus* | 623 | lqp | 0.8 | 500 | 10125.529 | 0.000 | 0.787 | 1.261 | 0.000 | 0.055 | Roughness_index, Aridity_index, Temp_max_Coldest | 0.438 |
| *Campylorhynchus gularis* | 216 | qp | 1.1 | 500 | 3277.300 | 3.117 | 0.906 | 1.614 | 0.000 | 0.075 | Roughness_index, Moisture_index, Temp_min_Warmest, Temp_min_Coldest | 0.203 |
| *Campylorhynchus jocosus* | 132 | qp | 0.9 | 500 | 1785.877 | 0.406 | 0.921 | 1.595 | 0.000 | 0.094 | Aridity_index, Moisture_index, Temp_max_Coldest, Elevation | 0.317 |
| *Campylorhynchus megalopterus* | 66 | lq | 1.1 | 500 | 980.519 | 2.311 | 0.858 | 1.342 | 0.000 | 0.125 | Slope, Aridity_index, Moisture_index, Temp_min_Warmest | 0.341 |
| *Campylorhynchus rufinucha* | 31 | lqp | 0.9 | 500 | 365.859 | 1.156 | 0.858 | 1.604 | 0.000 | 0.000 | pp_coldest_q, temp_mean_coldest_q, temp_range_diurnal, temp_seasonality | 0.439 |
| *Campylorhynchus zonatus* | 351 | lq | 1.0 | 500 | 5527.228 | 1.121 | 0.676 | 1.152 | 0.000 | 0.049 | Moisture_index, Temp_max_Coldest | 0.612 |
| *Caprimulgus carolinensis* | 939 | lqp | 1.0 | 500 | 15397.343 | 2.167 | 0.811 | 1.417 | 0.000 | 0.041 | Roughness_index, Moisture_index, Temp_max_Coldest | 0.336 |
| *Caprimulgus maculicaudus* | 204 | lqp | 0.9 | 500 | 3824.196 | 0.000 | 0.770 | 1.203 | 0.000 | 0.061 | Moisture_index, Temp_max_Coldest, Temp_min_Warmest, Temp_max_Warmest | 0.623 |
| *Caprimulgus ridgwayi* | 216 | qp | 1.0 | 500 | 3227.214 | 0.324 | 0.845 | 1.351 | 0.000 | 0.082 | Roughness_index, Moisture_index, Temp_min_Warmest | 0.450 |
| *Caprimulgus salvini* | 35 | lq | 0.8 | 500 | 529.356 | 0.000 | 0.676 | 1.367 | 0.000 | 0.000 | pp_coldest_q, temp_mean_coldest_q, temp_range_diurnal, temp_seasonality | 0.292 |
| *Caprimulgus vociferus* | 1350 | lqp | 1.1 | 500 | 24314.149 | 57.580 | 0.852 | 1.425 | 0.000 | 0.034 | Roughness_index, Temp_max_Warmest, Elevation | 0.420 |
| *Cardinalis cardinalis* | 2299 | lqp | 1.0 | 500 | 41391.063 | 2.073 | 0.739 | 1.247 | 0.000 | 0.056 | Temp_max_Coldest, Elevation | 0.449 |
| *Cardinalis phoeniceus* | 89 | lq | 1.1 | 500 | 857.772 | 1.380 | 0.805 | 1.356 | 0.000 | 0.125 | Slope, Precipitation | 0.369 |
| *Cardinalis sinuatus* | 1096 | qp | 0.8 | 500 | 18524.599 | 0.000 | 0.857 | 1.534 | 0.000 | 0.042 | Roughness_index, Temp_max_Coldest, Precipitation, Temp_max_Warmest | 0.412 |
| *Carduelis atriceps* | 36 | qp | 1.0 | 500 | 427.821 | 0.459 | 0.980 | 1.909 | 0.000 | 0.111 | Aridity_index, Temp_max_Coldest, Temp_min_Warmest, Precipitation | 0.158 |
| *Carduelis notata* | 325 | lq | 0.8 | 500 | 5204.436 | 1.739 | 0.829 | 1.222 | 0.000 | 0.013 | Aridity_index, Temp_min_Warmest, Precipitation | 0.491 |
| *Carduelis pinus* | 2644 | lqp | 0.8 | 500 | 50497.345 | 0.000 | 0.818 | 1.217 | 0.000 | 0.049 | Roughness_index, Precipitation, Temp_max_Warmest | 0.514 |
| *Carduelis psaltria* | 1975 | lqp | 0.8 | 500 | 36020.812 | 0.000 | 0.788 | 1.286 | 0.000 | 0.040 | Moisture_index, Temp_min_Coldest, Elevation | 0.497 |
| *Carduelis tristis* | 2104 | lqp | 0.8 | 500 | 38765.005 | 0.000 | 0.782 | 1.153 | 0.000 | 0.079 | Temp_max_Warmest, Elevation | 0.553 |
| *Carpodacus mexicanus* | 2795 | lqp | 0.9 | 500 | 52299.775 | 6.795 | 0.749 | 1.183 | 0.000 | 0.051 | Aridity_index, Temp_min_Coldest, Elevation | 0.525 |
| *Carpodectes nitidus* | 86 | qp | 0.9 | 500 | 1005.077 | 1.722 | 0.881 | 1.492 | 0.000 | 0.158 | Roughness_index, Temp_max_Coldest, Precipitation | 0.317 |
| *Caryothraustes canadensis* | 247 | lq | 0.8 | 500 | 4396.551 | 8.899 | 0.724 | 1.141 | 0.000 | 0.085 | Roughness_index, Moisture_index, Temp_max_Coldest | 0.680 |
| *Caryothraustes poliogaster* | 262 | lqp | 1.2 | 500 | 3823.323 | 3.556 | 0.816 | 1.347 | 0.000 | 0.063 | Roughness_index, Aridity_index, Moisture_index, Temp_max_Coldest | 0.439 |
| *Catharus aurantiirostris* | 545 | lqp | 1.2 | 500 | 8925.161 | 2.555 | 0.825 | 1.282 | 0.000 | 0.061 | Moisture_index, Elevation | 0.314 |
| *Catharus bicknelli* | 137 | lqp | 0.8 | 500 | 1921.660 | 0.000 | 0.960 | 1.620 | 0.000 | 0.176 | Aridity_index, Temp_max_Coldest, Elevation | 0.100 |
| *Catharus dryas* | 178 | lq | 0.8 | 500 | 2949.432 | 0.000 | 0.908 | 1.552 | 0.000 | 0.045 | Moisture_index, Temp_min_Warmest | 0.205 |
| *Catharus frantzii* | 168 | lq | 0.8 | 500 | 2432.810 | 0.406 | 0.900 | 1.535 | 0.000 | 0.095 | Slope, Moisture_index, Elevation | 0.123 |
| *Catharus fuscescens* | 1561 | lqp | 0.8 | 500 | 28278.034 | 0.000 | 0.883 | 1.372 | 0.000 | 0.071 | Slope, Precipitation, Temp_max_Warmest | 0.490 |
| *Catharus guttatus* | 2442 | lqp | 1.0 | 500 | 46327.920 | 74.929 | 0.834 | 1.222 | 0.000 | 0.040 | Roughness_index, Precipitation, Temp_max_Warmest | 0.578 |
| *Catharus mexicanus* | 123 | lq | 0.8 | 500 | 1876.454 | 0.000 | 0.778 | 1.145 | 0.000 | 0.129 | Moisture_index, Temp_min_Warmest | 0.396 |
| *Catharus minimus* | 1270 | lqp | 0.8 | 500 | 23180.211 | 0.000 | 0.822 | 1.274 | 0.000 | 0.062 | Moisture_index, Temp_max_Warmest, Temp_min_Coldest, Elevation | 0.448 |
| *Catharus occidentalis* | 225 | lqp | 1.1 | 500 | 3389.842 | 0.516 | 0.867 | 1.327 | 0.000 | 0.109 | Roughness_index, Moisture_index, Temp_max_Warmest | 0.224 |
| *Catherpes mexicanus* | 1966 | lqp | 0.8 | 500 | 36100.082 | 0.000 | 0.812 | 1.295 | 0.000 | 0.041 | Roughness_index, Moisture_index, Temp_max_Coldest, Elevation | 0.410 |
| *Celeus castaneus* | 129 | lq | 0.9 | 500 | 1778.472 | 0.045 | 0.764 | 1.307 | 0.000 | 0.034 | Aridity_index, Temp_max_Coldest, Temp_min_Warmest | 0.548 |
| *Cercomacra tyrannina* | 634 | lqp | 0.8 | 500 | 11158.142 | 0.000 | 0.755 | 1.171 | 0.000 | 0.027 | Moisture_index, Temp_max_Coldest, Temp_min_Warmest | 0.546 |
| *Chaetura cinereiventris* | 1248 | qp | 0.8 | 500 | 22534.132 | 0.000 | 0.805 | 1.248 | 0.000 | 0.054 | Roughness_index, Aridity_index, Temp_max_Warmest, Temp_min_Coldest | 0.409 |
| *Chaetura pelagica* | 1711 | lqp | 0.9 | 500 | 31076.026 | 11.400 | 0.837 | 1.442 | 0.000 | 0.056 | Roughness_index, Temp_min_Warmest, Precipitation | 0.396 |
| *Chaetura vauxi* | 1510 | lqp | 1.1 | 500 | 26223.743 | 11.907 | 0.864 | 1.414 | 0.000 | 0.036 | Moisture_index, Temp_min_Coldest, Elevation | 0.325 |
| *Chalybura buffonii* | 345 | lqp | 1.0 | 500 | 5397.089 | 0.667 | 0.815 | 1.362 | 0.000 | 0.060 | Roughness_index, Aridity_index | 0.282 |
| *Chalybura urochrysia* | 112 | lq | 0.8 | 500 | 1549.876 | 0.000 | 0.784 | 1.346 | 0.000 | 0.038 | Aridity_index, Temp_max_Coldest, Precipitation | 0.459 |
| *Chiroxiphia linearis* | 95 | qp | 0.8 | 500 | 1276.698 | 0.118 | 0.840 | 1.444 | 0.000 | 0.091 | Aridity_index, Temp_min_Warmest, Temp_min_Coldest | 0.473 |
| *Chloroceryle aenea* | 949 | lp | 0.8 | 500 | 16399.517 | 6.104 | 0.744 | 1.203 | 0.000 | 0.038 | Aridity_index, Temp_max_Coldest, Precipitation, Temp_max_Warmest, Elevation | 0.549 |
| *Chloroceryle amazona* | 1782 | lqp | 1.1 | 500 | 33162.597 | 24.309 | 0.664 | 1.101 | 0.000 | 0.036 | Roughness_index, Moisture_index, Temp_max_Coldest | 0.621 |
| *Chloroceryle americana* | 2052 | lqp | 0.9 | 500 | 37846.679 | 9.003 | 0.659 | 1.110 | 0.000 | 0.061 | Moisture_index, Temp_max_Coldest, Temp_max_Warmest, Elevation | 0.620 |
| *Chloroceryle inda* | 619 | lqp | 0.8 | 500 | 11393.556 | 0.361 | 0.761 | 1.174 | 0.000 | 0.062 | Aridity_index, Temp_max_Coldest, Precipitation, Elevation | 0.692 |
| *Chlorophanes spiza* | 739 | lqp | 0.8 | 500 | 13380.001 | 0.000 | 0.855 | 1.239 | 0.000 | 0.045 | Roughness_index, Moisture_index, Temp_max_Coldest | 0.367 |
| *Chlorophonia occipitalis* | 90 | qp | 0.9 | 500 | 1314.800 | 0.445 | 0.887 | 1.416 | 0.000 | 0.091 | Roughness_index, Moisture_index, Temp_max_Coldest, Temp_max_Warmest | 0.254 |
| *Chlorospingus ophthalmicus* | 475 | lqp | 0.9 | 500 | 8117.640 | 0.656 | 0.900 | 1.401 | 0.000 | 0.068 | Moisture_index, Temp_min_Warmest | 0.193 |
| *Chlorostilbon auriceps* | 152 | qp | 1.1 | 500 | 1795.070 | 0.616 | 0.730 | 1.178 | 0.000 | 0.060 | Roughness_index, Moisture_index, Temp_min_Warmest | 0.541 |
| *Chlorostilbon canivetii* | 280 | lqp | 0.8 | 500 | 3996.453 | 0.435 | 0.706 | 1.170 | 0.000 | 0.091 | Aridity_index, Moisture_index, Elevation | 0.477 |
| *Chlorostilbon russatus* | 26 | qp | 0.8 | 500 | 324.238 | 3.396 | 0.869 | 1.654 | 0.000 | 0.000 | Roughness_index, Precipitation | 0.425 |
| *Chlorostilbon swainsonii* | 99 | lq | 1.2 | 500 | 858.542 | 0.082 | 0.584 | 1.045 | 0.000 | 0.130 | Temp_min_Warmest, Precipitation | 0.685 |
| *Chlorothraupis carmioli* | 89 | lq | 0.8 | 500 | 1099.680 | 0.000 | 0.793 | 1.242 | 0.000 | 0.238 | Precipitation, Temp_max_Warmest, Elevation | 0.502 |
| *Chondestes grammacus* | 2468 | lq | 0.8 | 500 | 47047.617 | 0.323 | 0.795 | 1.237 | 0.000 | 0.049 | Moisture_index, Temp_max_Coldest, Elevation | 0.498 |
| *Chordeiles acutipennis* | 1655 | lqp | 0.8 | 500 | 28194.906 | 0.000 | 0.870 | 1.276 | 0.000 | 0.044 | Roughness_index, Aridity_index, Temp_max_Coldest, Temp_max_Warmest | 0.356 |
| *Chordeiles minor* | 2773 | lqp | 0.8 | 500 | 54378.139 | 0.000 | 0.773 | 1.282 | 0.000 | 0.051 | Moisture_index, Temp_max_Coldest, Elevation | 0.437 |
| *Chordeiles rupestris* | 227 | lq | 1.0 | 500 | 4155.141 | 0.001 | 0.659 | 1.099 | 0.000 | 0.140 | Aridity_index, Temp_max_Coldest, Temp_min_Warmest | 0.477 |
| *Chrysomus icterocephalus* | 525 | lp | 1.0 | 500 | 8853.117 | 3.911 | 0.830 | 1.309 | 0.000 | 0.008 | Roughness_index, Moisture_index, Temp_max_Coldest, Temp_min_Coldest | 0.563 |
| *Chrysomus ruficapillus* | 2272 | lqp | 0.9 | 500 | 41062.925 | 12.877 | 0.826 | 1.333 | 0.000 | 0.036 | Aridity_index, Moisture_index, Temp_max_Coldest, Elevation | 0.483 |
| *Cinclus mexicanus* | 1726 | lqp | 1.1 | 500 | 31284.003 | 11.394 | 0.898 | 1.454 | 0.000 | 0.055 | Roughness_index, Moisture_index, Temp_max_Coldest | 0.355 |
| *Cistothorus palustris* | 1813 | lqp | 1.0 | 500 | 32823.994 | 7.393 | 0.763 | 1.179 | 0.000 | 0.060 | Aridity_index, Temp_max_Coldest, Elevation | 0.517 |
| *Cistothorus platensis* | 1185 | lqp | 0.8 | 500 | 22527.277 | 0.000 | 0.863 | 1.325 | 0.000 | 0.049 | Moisture_index, Temp_min_Coldest, Elevation | 0.447 |
| *Coccothraustes abeillei* | 117 | lq | 0.8 | 500 | 1727.565 | 0.000 | 0.877 | 1.317 | 0.000 | 0.036 | Aridity_index, Moisture_index, Temp_max_Coldest, Temp_min_Warmest | 0.446 |
| *Coccothraustes vespertinus* | 2143 | lqp | 0.9 | 500 | 40911.297 | 53.778 | 0.851 | 1.296 | 0.000 | 0.061 | Roughness_index, Precipitation, Temp_max_Warmest | 0.552 |
| *Coccyzus americanus* | 1967 | lq | 0.8 | 500 | 36306.258 | 89.636 | 0.789 | 1.370 | 0.000 | 0.047 | Roughness_index, Temp_min_Warmest, Precipitation | 0.408 |
| *Coccyzus erythropthalmus* | 1530 | lqp | 0.8 | 500 | 27873.337 | 0.777 | 0.876 | 1.383 | 0.000 | 0.048 | Temp_max_Warmest, Elevation | 0.490 |
| *Coccyzus euleri* | 190 | qp | 0.8 | 500 | 3626.359 | 0.434 | 0.672 | 1.077 | 0.143 | 0.089 | Slope, Aridity_index, Precipitation, Temp_max_Warmest | 0.636 |
| *Coccyzus minor* | 494 | lqp | 0.8 | 500 | 5432.977 | 0.000 | 0.746 | 1.183 | 0.000 | 0.067 | Roughness_index, Aridity_index, Temp_max_Warmest, Temp_min_Coldest | 0.485 |
| *Coereba flaveola* | 1437 | lqp | 1.0 | 500 | 24378.830 | 13.500 | 0.752 | 1.142 | 0.000 | 0.080 | Slope, Aridity_index, Temp_max_Warmest, Temp_min_Coldest | 0.468 |
| *Colaptes atricollis* | 125 | qp | 1.1 | 500 | 1928.141 | 1.161 | 0.789 | 1.284 | 0.000 | 0.067 | Slope, Moisture_index, Temp_min_Warmest | 0.400 |
| *Colaptes auratus* | 3160 | lqp | 1.2 | 500 | 61796.657 | 209.174 | 0.798 | 1.328 | 0.000 | 0.072 | Precipitation, Temp_max_Warmest, Elevation | 0.490 |
| *Colaptes auricularis* | 91 | lq | 1.0 | 500 | 1272.644 | 1.755 | 0.722 | 1.252 | 0.000 | 0.143 | Slope, Aridity_index, Moisture_index, Temp_min_Warmest | 0.474 |
| *Colaptes chrysoides* | 467 | lqp | 0.9 | 500 | 6762.571 | 6.681 | 0.883 | 1.532 | 0.000 | 0.065 | Roughness_index, Temp_min_Warmest, Precipitation | 0.317 |
| *Colaptes rubiginosus* | 606 | lqp | 0.8 | 500 | 10604.507 | 0.000 | 0.859 | 1.331 | 0.000 | 0.027 | Roughness_index, Moisture_index, Temp_max_Coldest | 0.306 |
| *Colibri delphinae* | 275 | lqp | 0.9 | 500 | 4654.032 | 1.482 | 0.875 | 1.252 | 0.000 | 0.088 | Moisture_index, Temp_max_Warmest | 0.237 |
| *Colibri thalassinus* | 285 | lqp | 1.2 | 500 | 4826.236 | 1.315 | 0.919 | 1.550 | 0.000 | 0.014 | Moisture_index, Temp_min_Warmest | 0.269 |
| *Colinus cristatus* | 730 | qp | 1.2 | 500 | 12464.563 | 0.096 | 0.738 | 1.118 | 0.008 | 0.023 | Moisture_index, Temp_min_Warmest | 0.588 |
| *Colinus leucopogon* | 15 | lqph | 1.0 | 500 | NA | NA | 0.633 | 1.386 | 0.000 | 0.500 | Slope, Roughness_index, Aridity_index, Moisture_index, Temp_max_Coldest, Temp_min_Warmest, Precipitation, Temp_max_Warmest, Temp_min_Coldest, Elevation | 0.643 |
| *Colinus nigrogularis* | 113 | lq | 0.9 | 500 | 1420.930 | 1.772 | 0.749 | 1.195 | 0.000 | 0.240 | Aridity_index, Moisture_index, Temp_max_Coldest, Elevation | 0.490 |
| *Colinus virginianus* | 1834 | lqp | 0.9 | 500 | 33970.559 | 1.567 | 0.803 | 1.350 | 0.000 | 0.050 | Temp_max_Coldest, Elevation | 0.366 |
| *Colonia colonus* | 1234 | lqp | 0.8 | 500 | 22814.433 | 3.321 | 0.816 | 1.160 | 0.000 | 0.040 | Roughness_index, Moisture_index, Temp_max_Coldest | 0.516 |
| *Conopias albovittatus* | 494 | lp | 0.8 | 500 | 8829.651 | 2.141 | 0.786 | 1.253 | 0.000 | 0.017 | Roughness_index, Precipitation, Temp_max_Warmest, Temp_min_Coldest | 0.477 |
| *Contopus cinereus* | 620 | lqp | 0.8 | 500 | 10858.964 | 2.211 | 0.861 | 1.273 | 0.000 | 0.014 | Roughness_index, Aridity_index, Moisture_index, Temp_min_Warmest | 0.477 |
| *Contopus cooperi* | 2559 | lqp | 0.8 | 500 | 49116.129 | 0.000 | 0.800 | 1.118 | 0.000 | 0.072 | Roughness_index, Precipitation, Temp_max_Warmest | 0.540 |
| *Contopus pertinax* | 557 | lqp | 1.0 | 500 | 9174.906 | 0.623 | 0.858 | 1.470 | 0.000 | 0.022 | Roughness_index, Moisture_index, Temp_max_Coldest, Temp_max_Warmest | 0.285 |
| *Contopus sordidulus* | 2687 | lqp | 0.8 | 500 | 51142.339 | 0.000 | 0.817 | 1.316 | 0.000 | 0.053 | Roughness_index, Moisture_index, Temp_max_Coldest | 0.456 |
| *Corvus brachyrhynchos* | 2356 | lqp | 1.0 | 500 | 44050.752 | 94.478 | 0.855 | 1.403 | 0.000 | 0.030 | Precipitation, Temp_max_Warmest, Elevation | 0.469 |
| *Corvus corax* | 4269 | lqp | 1.2 | 500 | 88080.364 | 54.538 | 0.838 | 1.434 | 0.000 | 0.039 | Roughness_index, Moisture_index, Temp_max_Coldest | 0.432 |
| *Corvus cryptoleucus* | 1142 | lqp | 0.8 | 500 | 19880.545 | 0.591 | 0.875 | 1.520 | 0.000 | 0.063 | Slope, Moisture_index, Temp_max_Coldest, Elevation | 0.329 |
| *Corvus imparatus* | 155 | lqp | 0.8 | 500 | 1993.581 | 0.000 | 0.937 | 1.711 | 0.000 | 0.028 | Temp_max_Coldest, Temp_min_Warmest | 0.297 |
| *Corvus sinaloae* | 125 | lqp | 0.8 | 500 | 1505.410 | 0.000 | 0.942 | 1.674 | 0.000 | 0.000 | Slope, Temp_max_Coldest, Temp_min_Warmest | 0.563 |
| *Cotinga amabilis* | 77 | lq | 0.8 | 500 | 1085.518 | 0.000 | 0.802 | 1.307 | 0.000 | 0.111 | Roughness_index, Aridity_index, Moisture_index, Temp_max_Coldest | 0.398 |
| *Crax rubra* | 225 | lqp | 0.9 | 500 | 2990.913 | 0.000 | 0.791 | 1.315 | 0.000 | 0.043 | Moisture_index, Elevation | 0.569 |
| *Crotophaga sulcirostris* | 1012 | lqp | 0.8 | 500 | 16528.037 | 0.000 | 0.684 | 1.122 | 0.000 | 0.079 | Aridity_index, Elevation | 0.435 |
| *Crypturellus boucardi* | 175 | lqp | 0.8 | 500 | 2673.903 | 0.000 | 0.841 | 1.425 | 0.000 | 0.000 | Roughness_index, Aridity_index, Moisture_index, Temp_max_Coldest | 0.426 |
| *Crypturellus cinnamomeus* | 260 | qp | 1.1 | 500 | 3878.875 | 0.000 | 0.791 | 1.415 | 0.000 | 0.052 | Roughness_index, Temp_max_Warmest, Temp_min_Coldest | 0.392 |
| *Crypturellus soui* | 985 | lqp | 0.9 | 500 | 18160.123 | 0.000 | 0.786 | 1.191 | 0.000 | 0.072 | Roughness_index, Aridity_index, Temp_max_Warmest, Temp_min_Coldest | 0.399 |
| *Curaeus curaeus* | 801 | lq | 0.9 | 500 | 11126.389 | 3.437 | 0.818 | 1.317 | 0.000 | 0.091 | Roughness_index, Moisture_index, Temp_max_Coldest | 0.308 |
| *Curaeus forbesi* | 21 | lp | 1.2 | 500 | 329.078 | 0.000 | 0.743 | 1.430 | 0.000 | 0.200 | pp_coldest_q, temp_mean_coldest_q, temp_range_diurnal, temp_seasonality | 0.528 |
| *Cyanerpes caeruleus* | 821 | lqp | 1.1 | 500 | 15107.336 | 3.708 | 0.788 | 1.176 | 0.000 | 0.065 | Roughness_index, Moisture_index, Temp_max_Coldest | 0.459 |
| *Cyanerpes cyaneus* | 594 | lq | 0.9 | 500 | 10355.232 | 3.304 | 0.735 | 1.111 | 0.000 | 0.075 | Roughness_index, Moisture_index, Temp_max_Coldest, Temp_max_Warmest | 0.479 |
| *Cyanerpes lucidus* | 126 | lq | 1.1 | 500 | 1651.554 | 0.000 | 0.858 | 1.461 | 0.000 | 0.036 | Roughness_index, Moisture_index, Temp_max_Coldest | 0.486 |
| *Cyanerpes nitidus* | 211 | lqp | 0.9 | 500 | 3952.016 | 1.875 | 0.701 | 1.162 | 0.000 | 0.057 | Aridity_index, Temp_max_Coldest, Temp_min_Warmest | 0.401 |
| *Cyanocitta cristata* | 2033 | lqp | 1.1 | 500 | 37372.736 | 2.393 | 0.813 | 1.334 | 0.000 | 0.035 | Temp_max_Coldest, Elevation | 0.390 |
| *Cyanocitta stelleri* | 1881 | lqp | 0.8 | 500 | 32791.720 | 0.000 | 0.887 | 1.378 | 0.000 | 0.060 | Roughness_index, Moisture_index, Temp_max_Coldest, Elevation | 0.349 |
| *Cyanocompsa cyanoides* | 418 | lqp | 0.8 | 500 | 6332.525 | 0.000 | 0.722 | 1.160 | 0.000 | 0.075 | Moisture_index, Elevation | 0.447 |
| *Cyanocompsa parellina* | 348 | lqp | 0.9 | 500 | 4790.512 | 0.705 | 0.770 | 1.245 | 0.000 | 0.097 | Moisture_index, Temp_min_Warmest | 0.423 |
| *Cyanocorax affinis* | 419 | lqp | 0.9 | 500 | 6002.409 | 0.355 | 0.687 | 1.119 | 0.000 | 0.053 | Moisture_index, Temp_min_Coldest | 0.572 |
| *Cyanocorax beecheii* | 65 | lq | 0.8 | 500 | 882.407 | 0.000 | 0.932 | 1.711 | 0.000 | 0.000 | Roughness_index, Temp_max_Coldest | 0.797 |
| *Cyanocorax cayanus* | 130 | lq | 1.1 | 500 | 2232.969 | 1.415 | 0.795 | 1.237 | 0.000 | 0.156 | Slope, Temp_max_Coldest, Temp_min_Warmest, Precipitation | 0.334 |
| *Cyanocorax chrysops* | 1669 | lqp | 0.9 | 500 | 30531.630 | 19.580 | 0.846 | 1.411 | 0.000 | 0.041 | Roughness_index, Aridity_index, Moisture_index, Temp_max_Coldest, Temp_min_Warmest | 0.462 |
| *Cyanocorax cyanopogon* | 649 | lqp | 0.8 | 500 | 11373.569 | 0.654 | 0.774 | 1.171 | 0.008 | 0.025 | Roughness_index, Moisture_index, Temp_min_Coldest | 0.459 |
| *Cyanocorax dickeyi* | 22 | lq | 1.0 | 500 | 315.533 | 1.435 | 0.862 | 1.638 | 0.000 | 0.000 | pp_coldest_q, temp_mean_coldest_q, temp_range_diurnal, temp_seasonality | 0.619 |
| *Cyanocorax heilprini* | 16 | lq | 0.8 | 500 | 269.336 | 0.000 | 0.709 | 1.475 | 0.000 | 0.000 | pp_coldest_q, temp_mean_coldest_q, temp_range_diurnal, temp_seasonality | 0.610 |
| *Cyanocorax melanocyaneus* | 147 | lqp | 1.2 | 500 | 2042.736 | 5.702 | 0.871 | 1.301 | 0.000 | 0.108 | Moisture_index, Temp_max_Coldest, Temp_min_Warmest | 0.298 |
| *Cyanocorax morio* | 336 | lqp | 0.9 | 500 | 4845.076 | 0.000 | 0.729 | 1.211 | 0.000 | 0.093 | Aridity_index, Moisture_index, Temp_max_Coldest, Elevation | 0.468 |
| *Cyanocorax sanblasianus* | 152 | qp | 1.2 | 500 | 1429.734 | 4.974 | 0.820 | 1.483 | 0.000 | 0.000 | Roughness_index, Moisture_index, Temp_max_Coldest | 0.498 |
| *Cyanocorax yucatanicus* | 151 | lqp | 1.2 | 500 | 1758.761 | 0.376 | 0.835 | 1.434 | 0.000 | 0.031 | Aridity_index, Moisture_index, Temp_max_Coldest, Temp_min_Warmest, Temp_min_Coldest | 0.453 |
| *Cyanoloxia glaucocaerulea* | 246 | lqp | 0.8 | 500 | 3808.032 | 0.000 | 0.890 | 1.568 | 0.000 | 0.121 | Temp_max_Coldest, Precipitation, Elevation | 0.402 |
| *Cyanolyca argentigula* | 22 | lp | 1.0 | 500 | 196.443 | 0.263 | 0.956 | 1.841 | 0.000 | 0.167 | Aridity_index, Elevation | 0.120 |
| *Cyanolyca cucullata* | 58 | lq | 0.8 | 500 | 872.056 | 0.000 | 0.951 | 1.808 | 0.000 | 0.000 | Roughness_index, Moisture_index, Temp_max_Coldest | 0.626 |
| *Cyanolyca mirabilis* | 15 | qp | 1.1 | 500 | 175.367 | 0.803 | 0.761 | 1.523 | 0.000 | 0.667 | pp_coldest_q, temp_mean_coldest_q, temp_range_diurnal, temp_seasonality | 0.565 |
| *Cyanolyca nana* | 17 | lq | 0.9 | 500 | 211.583 | 0.036 | 0.655 | 1.382 | 0.000 | 0.750 | Aridity_index, Temp_max_Warmest | 0.207 |
| *Cyanolyca pulchra* | 32 | lqp | 0.8 | 500 | 452.728 | 0.000 | 0.605 | 1.254 | 0.000 | 0.375 | pp_coldest_q, temp_mean_coldest_q, temp_range_diurnal, temp_seasonality | 0.546 |
| *Cyanolyca pumilo* | 42 | qp | 1.1 | 500 | 579.195 | 0.230 | 0.955 | 1.858 | 0.000 | 0.000 | pp_coldest_q, temp_mean_coldest_q, temp_range_diurnal, temp_seasonality | 0.315 |
| *Cyclarhis gujanensis* | 1968 | lqp | 1.2 | 500 | 37267.834 | 19.602 | 0.730 | 1.071 | 0.000 | 0.053 | Moisture_index, Temp_max_Coldest, Elevation | 0.539 |
| *Cyclarhis nigrirostris* | 163 | lqp | 1.1 | 500 | 2351.206 | 0.371 | 0.807 | 1.299 | 0.000 | 0.220 | Moisture_index, Elevation | 0.404 |
| *Cymbilaimus lineatus* | 849 | lqp | 0.9 | 500 | 16136.357 | 1.883 | 0.718 | 1.195 | 0.000 | 0.033 | Roughness_index, Moisture_index, Temp_max_Coldest | 0.484 |
| *Cymbilaimus sanctaemariae* | 68 | lp | 0.9 | 500 | 1180.280 | 0.140 | 0.713 | 1.182 | 0.000 | 0.118 | pp_coldest_q, temp_mean_coldest_q, temp_range_diurnal, temp_seasonality | 0.889 |
| *Cynanthus latirostris* | 856 | lqp | 0.8 | 500 | 13453.691 | 0.000 | 0.728 | 1.210 | 0.000 | 0.066 | Roughness_index, Moisture_index, Temp_min_Warmest | 0.415 |
| *Cynanthus sordidus* | 86 | lq | 1.1 | 500 | 1161.895 | 0.883 | 0.903 | 1.521 | 0.000 | 0.079 | Aridity_index, Moisture_index, Temp_max_Coldest, Elevation | 0.467 |
| *Cyphorhinus phaeocephalus* | 127 | lqp | 0.8 | 500 | 1826.159 | 0.361 | 0.795 | 1.223 | 0.000 | 0.067 | Precipitation, Temp_max_Warmest | 0.542 |
| *Cypseloides cryptus* | 77 | lq | 0.8 | 500 | 1293.333 | 0.000 | 0.842 | 1.332 | 0.000 | 0.111 | Roughness_index, Moisture_index, Temp_max_Coldest, Temp_max_Warmest | 0.383 |
| *Cypseloides niger* | 1140 | lqp | 1.2 | 500 | 18951.792 | 59.969 | 0.842 | 1.258 | 0.000 | 0.042 | Roughness_index, Precipitation, Temp_max_Warmest, Elevation | 0.405 |
| *Cypseloides storeri* | 10 | lq | 1.2 | 500 | 172.556 | 1.520 | 0.979 | 1.959 | 0.000 | 0.000 | pp_coldest_q, temp_mean_coldest_q, temp_range_diurnal, temp_seasonality | 0.520 |
| *Cyrtonyx montezumae* | 380 | lqp | 0.8 | 500 | 6427.686 | 0.000 | 0.890 | 1.442 | 0.000 | 0.074 | Roughness_index, Temp_max_Coldest, Precipitation, Elevation | 0.372 |
| *Cyrtonyx ocellatus* | 46 | qp | 0.8 | 500 | 639.110 | 0.000 | 0.823 | 1.376 | 0.000 | 0.167 | Moisture_index, Temp_max_Coldest, Temp_min_Warmest | 0.291 |
| *Dacnis cayana* | 1425 | lqp | 1.0 | 500 | 25981.223 | 15.100 | 0.793 | 1.164 | 0.000 | 0.054 | Roughness_index, Moisture_index, Temp_max_Coldest | 0.490 |
| *Dactylortyx thoracicus* | 126 | lqp | 0.8 | 500 | 1941.793 | 0.000 | 0.876 | 1.503 | 0.000 | 0.000 | Moisture_index, Temp_max_Warmest | 0.369 |
| *Deconychura longicauda* | 62 | lqp | 0.9 | 500 | 1069.416 | 0.809 | 0.880 | 1.618 | 0.000 | 0.000 | pp_coldest_q, temp_mean_coldest_q, temp_range_diurnal, temp_seasonality | 0.505 |
| *Deconychura stictolaema* | 152 | qp | 0.8 | 500 | 2868.582 | 0.145 | 0.617 | 1.072 | 0.000 | 0.105 | Roughness_index, Aridity_index, Temp_max_Coldest | 0.663 |
| *Deltarhynchus flammulatus* | 81 | qp | 0.9 | 500 | 807.330 | 1.536 | 0.688 | 1.244 | 0.000 | 0.214 | Roughness_index, Aridity_index, Moisture_index, Temp_max_Warmest | 0.516 |
| *Dendrocincla anabatina* | 139 | lq | 1.2 | 500 | 1932.618 | 0.642 | 0.758 | 1.188 | 0.000 | 0.129 | Roughness_index, Aridity_index, Temp_max_Coldest | 0.564 |
| *Dendrocincla homochroa* | 180 | lqp | 1.0 | 500 | 2741.204 | 1.527 | 0.767 | 1.257 | 0.000 | 0.048 | Aridity_index, Precipitation, Temp_max_Warmest, Temp_min_Coldest | 0.468 |
| *Dendrocolaptes certhia* | 497 | lq | 0.8 | 500 | 9630.420 | 6.425 | 0.701 | 1.247 | 0.000 | 0.016 | Roughness_index, Temp_max_Coldest, Precipitation | 0.416 |
| *Dendrocolaptes picumnus* | 632 | lqp | 0.8 | 500 | 12238.868 | 0.000 | 0.778 | 1.189 | 0.000 | 0.025 | Roughness_index, Aridity_index, Temp_max_Coldest, Temp_max_Warmest | 0.580 |
| *Dendrocolaptes platyrostris* | 895 | lqp | 0.8 | 500 | 15489.849 | 0.000 | 0.857 | 1.272 | 0.000 | 0.106 | Roughness_index, Aridity_index, Moisture_index, Temp_max_Warmest, Temp_min_Coldest | 0.351 |
| *Dendrocolaptes sanctithomae* | 246 | lqp | 0.8 | 500 | 3470.749 | 0.000 | 0.764 | 1.262 | 0.000 | 0.019 | Moisture_index, Elevation | 0.450 |
| *Dendrortyx barbatus* | 44 | lq | 1.2 | 500 | 573.896 | 0.843 | 0.913 | 1.672 | 0.000 | 0.091 | pp_coldest_q, temp_mean_coldest_q, temp_range_diurnal, temp_seasonality | 0.281 |
| *Dendrortyx leucophrys* | 59 | lq | 0.9 | 500 | 795.927 | 0.737 | 0.921 | 1.626 | 0.000 | 0.133 | Moisture_index, Temp_max_Coldest, Temp_min_Warmest | 0.167 |
| *Dendrortyx macroura* | 79 | lp | 1.0 | 500 | 1136.071 | 1.832 | 0.890 | 1.519 | 0.000 | 0.000 | Slope, Aridity_index, Moisture_index, Temp_max_Coldest, Temp_max_Warmest | 0.293 |
| *Diglossa baritula* | 174 | lq | 1.1 | 500 | 2531.539 | 0.347 | 0.923 | 1.593 | 0.000 | 0.023 | Moisture_index, Temp_min_Warmest | 0.217 |
| *Diglossa plumbea* | 53 | qp | 1.0 | 500 | 585.668 | 0.118 | 0.936 | 1.775 | 0.000 | 0.000 | Precipitation, Temp_max_Warmest | 0.341 |
| *Dives atroviolaceus* | 131 | lqp | 0.8 | 500 | 1186.819 | 0.000 | 0.473 | 1.032 | 0.000 | 0.130 | Aridity_index, Temp_min_Warmest, Precipitation | 0.596 |
| *Dives dives* | 291 | lqp | 0.8 | 500 | 4248.065 | 0.000 | 0.628 | 1.080 | 0.000 | 0.031 | Aridity_index, Temp_max_Warmest | 0.532 |
| *Dolichonyx oryzivorus* | 1674 | lqp | 0.8 | 500 | 31167.453 | 0.000 | 0.812 | 1.317 | 0.000 | 0.067 | Moisture_index, Temp_max_Coldest | 0.437 |
| *Doricha eliza* | 24 | lq | 1.0 | 500 | 233.514 | 0.305 | 0.504 | 1.247 | 0.000 | 0.500 | pp_coldest_q, temp_mean_coldest_q, temp_range_diurnal, temp_seasonality | 0.731 |
| *Doricha enicura* | 32 | lq | 0.8 | 500 | 454.894 | 2.625 | 0.934 | 1.808 | 0.000 | 0.000 | pp_coldest_q, temp_mean_coldest_q, temp_range_diurnal, temp_seasonality | 0.249 |
| *Dromococcyx pavoninus* | 305 | lq | 0.9 | 500 | 5555.209 | 1.950 | 0.872 | 1.247 | 0.000 | 0.053 | Moisture_index, Temp_max_Coldest, Elevation | 0.299 |
| *Dromococcyx phasianellus* | 391 | lqp | 1.0 | 500 | 7571.028 | 8.005 | 0.656 | 1.138 | 0.000 | 0.128 | Roughness_index, Moisture_index, Temp_max_Coldest | 0.547 |
| *Dryocopus lineatus* | 1618 | lqp | 1.1 | 500 | 29386.971 | 4.934 | 0.700 | 1.142 | 0.000 | 0.046 | Roughness_index, Aridity_index, Temp_max_Warmest, Temp_min_Coldest | 0.560 |
| *Dumetella carolinensis* | 1808 | lqp | 0.8 | 500 | 33322.821 | 0.000 | 0.808 | 1.199 | 0.000 | 0.076 | Temp_max_Warmest, Elevation | 0.486 |
| *Dysithamnus mentalis* | 1443 | lqp | 0.8 | 500 | 25943.972 | 0.000 | 0.873 | 1.334 | 0.000 | 0.040 | Aridity_index, Temp_max_Warmest, Temp_min_Coldest | 0.353 |
| *Dysithamnus striaticeps* | 28 | lq | 0.8 | 500 | 368.783 | 0.000 | 0.832 | 1.566 | 0.000 | 0.143 | pp_coldest_q, temp_mean_coldest_q, temp_range_diurnal, temp_seasonality | 0.378 |
| *Elaenia flavogaster* | 1352 | lqp | 0.9 | 500 | 24055.416 | 7.883 | 0.745 | 1.093 | 0.000 | 0.059 | Moisture_index, Temp_max_Coldest | 0.541 |
| *Elaenia frantzii* | 352 | lq | 1.0 | 500 | 5458.740 | 1.715 | 0.866 | 1.496 | 0.000 | 0.103 | Aridity_index, Temp_min_Warmest, Precipitation | 0.249 |
| *Electron carinatum* | 52 | lq | 0.8 | 500 | 808.338 | 1.540 | 0.559 | 1.165 | 0.000 | 0.308 | Slope, Temp_max_Coldest, Temp_max_Warmest | 0.485 |
| *Electron platyrhynchum* | 348 | lqp | 0.8 | 500 | 6233.382 | 0.000 | 0.793 | 1.189 | 0.000 | 0.094 | Roughness_index, Aridity_index, Temp_max_Warmest, Temp_min_Coldest | 0.525 |
| *Elvira chionura* | 25 | lqp | 0.9 | 500 | 267.532 | 2.195 | 0.909 | 1.596 | 0.000 | 0.053 | Moisture_index, Temp_min_Coldest | 0.286 |
| *Elvira cupreiceps* | 21 | lq | 1.2 | 500 | 236.359 | 4.076 | 0.882 | 1.601 | 0.000 | 0.250 | Precipitation, Elevation | 0.434 |
| *Empidonax affinis* | 240 | lq | 1.1 | 500 | 3965.588 | 0.238 | 0.812 | 1.351 | 0.000 | 0.017 | Slope, Moisture_index | 0.463 |
| *Empidonax albigularis* | 193 | lqp | 1.2 | 500 | 3021.233 | 0.791 | 0.809 | 1.323 | 0.000 | 0.170 | Slope, Moisture_index, Temp_min_Warmest | 0.241 |
| *Empidonax alnorum* | 2005 | lqp | 0.8 | 500 | 37012.449 | 0.000 | 0.852 | 1.268 | 0.000 | 0.053 | Precipitation, Temp_max_Warmest, Elevation | 0.559 |
| *Empidonax atriceps* | 34 | lq | 1.2 | 500 | 348.832 | 0.082 | 0.965 | 1.887 | 0.000 | 0.000 | Aridity_index, Temp_max_Warmest | 0.214 |
| *Empidonax difficilis* | 1241 | lqp | 0.8 | 500 | 18746.479 | 0.268 | 0.929 | 1.557 | 0.000 | 0.082 | Roughness_index, Moisture_index, Temp_max_Coldest, Elevation | 0.238 |
| *Empidonax flavescens* | 120 | lqp | 0.8 | 500 | 1701.548 | 0.000 | 0.894 | 1.414 | 0.000 | 0.033 | Moisture_index, Temp_max_Coldest, Temp_min_Warmest | 0.309 |
| *Empidonax fulvifrons* | 349 | lqp | 1.1 | 500 | 5668.602 | 1.715 | 0.883 | 1.484 | 0.000 | 0.138 | Roughness_index, Moisture_index, Temp_min_Coldest | 0.242 |
| *Empidonax oberholseri* | 1782 | lqp | 0.9 | 500 | 32797.266 | 6.229 | 0.849 | 1.332 | 0.000 | 0.054 | Moisture_index, Temp_min_Coldest, Elevation | 0.511 |
| *Empidonax occidentalis* | 1239 | lqp | 1.0 | 500 | 22372.694 | 4.390 | 0.837 | 1.255 | 0.000 | 0.062 | Moisture_index, Temp_max_Coldest, Elevation | 0.317 |
| *Empidonax traillii* | 2106 | lqp | 0.9 | 500 | 39836.806 | 3.774 | 0.713 | 1.160 | 0.000 | 0.067 | Aridity_index, Temp_max_Coldest | 0.572 |
| *Empidonax wrightii* | 1773 | lqp | 0.9 | 500 | 31704.093 | 10.369 | 0.840 | 1.382 | 0.000 | 0.032 | Roughness_index, Temp_max_Coldest, Precipitation | 0.457 |
| *Epinecrophylla fulviventris* | 153 | lqp | 0.8 | 500 | 2126.053 | 0.001 | 0.798 | 1.349 | 0.000 | 0.057 | Temp_max_Warmest, Temp_min_Coldest | 0.453 |
| *Eucometis penicillata* | 735 | lp | 0.8 | 500 | 13761.374 | 0.000 | 0.654 | 1.099 | 0.000 | 0.074 | Aridity_index, Temp_min_Warmest, Temp_max_Warmest | 0.628 |
| *Eugenes fulgens* | 464 | qp | 0.8 | 500 | 7688.124 | 1.976 | 0.833 | 1.197 | 0.000 | 0.070 | Roughness_index, Moisture_index, Temp_min_Warmest | 0.398 |
| *Eumomota superciliosa* | 143 | lqp | 0.8 | 500 | 1688.170 | 0.000 | 0.749 | 1.234 | 0.000 | 0.111 | Aridity_index, Moisture_index, Temp_min_Warmest | 0.543 |
| *Euphagus carolinus* | 1672 | lqp | 1.2 | 500 | 31560.208 | 19.518 | 0.773 | 1.189 | 0.000 | 0.053 | Moisture_index, Temp_max_Coldest, Elevation | 0.431 |
| *Euphagus cyanocephalus* | 2326 | lqp | 0.8 | 500 | 42896.633 | 0.000 | 0.863 | 1.353 | 0.000 | 0.039 | Moisture_index, Temp_min_Warmest, Temp_min_Coldest, Elevation | 0.389 |
| *Eupherusa cyanophrys* | 15 | lq | 0.9 | 500 | 192.091 | 0.000 | 0.943 | 1.850 | 0.000 | 0.000 | pp_coldest_q, temp_mean_coldest_q, temp_range_diurnal, temp_seasonality | 0.808 |
| *Eupherusa eximia* | 120 | lq | 0.8 | 500 | 1765.589 | 0.000 | 0.827 | 1.186 | 0.000 | 0.100 | Moisture_index, Temp_max_Coldest, Temp_min_Warmest | 0.259 |
| *Eupherusa poliocerca* | 19 | lp | 0.8 | 500 | 240.889 | 0.653 | 0.857 | 1.720 | 0.000 | 0.250 | pp_coldest_q, temp_mean_coldest_q, temp_range_diurnal, temp_seasonality | 0.521 |
| *Euphonia affinis* | 402 | lqp | 1.0 | 500 | 5642.012 | 0.556 | 0.793 | 1.340 | 0.000 | 0.012 | Precipitation, Elevation | 0.551 |
| *Euphonia elegantissima* | 378 | lqp | 0.9 | 500 | 6076.199 | 0.279 | 0.841 | 1.310 | 0.000 | 0.086 | Roughness_index, Moisture_index, Temp_min_Warmest | 0.328 |
| *Euphonia gouldi* | 163 | qp | 1.0 | 500 | 2261.912 | 1.041 | 0.845 | 1.312 | 0.000 | 0.081 | Aridity_index, Moisture_index, Temp_max_Coldest, Elevation | 0.417 |
| *Euphonia hirundinacea* | 280 | lqp | 0.9 | 500 | 4181.673 | 0.000 | 0.739 | 1.267 | 0.000 | 0.031 | Aridity_index, Moisture_index, Temp_max_Coldest, Temp_min_Warmest | 0.379 |
| *Euphonia luteicapilla* | 147 | qp | 0.9 | 500 | 1747.433 | 1.265 | 0.813 | 1.392 | 0.000 | 0.100 | Moisture_index, Temp_max_Coldest, Elevation | 0.347 |
| *Euphonia minuta* | 498 | lqp | 1.1 | 500 | 9247.517 | 3.404 | 0.761 | 1.171 | 0.000 | 0.074 | Roughness_index, Aridity_index, Temp_max_Coldest | 0.447 |
| *Euptilotis neoxenus* | 91 | qp | 1.2 | 500 | 1399.623 | 0.081 | 0.796 | 1.336 | 0.000 | 0.087 | pp_coldest_q, temp_mean_coldest_q, temp_range_diurnal, temp_seasonality | 0.376 |
| *Florisuga fusca* | 607 | lqp | 0.9 | 500 | 8854.371 | 7.162 | 0.939 | 1.654 | 0.000 | 0.045 | Moisture_index, Temp_max_Coldest, Temp_max_Warmest, Elevation | 0.229 |
| *Florisuga mellivora* | 672 | lqp | 0.8 | 500 | 11894.930 | 5.056 | 0.797 | 1.274 | 0.000 | 0.032 | Roughness_index, Temp_max_Warmest, Temp_min_Coldest | 0.520 |
| *Formicarius analis* | 649 | lqp | 1.0 | 500 | 11902.590 | 8.437 | 0.808 | 1.234 | 0.000 | 0.082 | Roughness_index, Aridity_index, Temp_max_Warmest, Temp_min_Coldest | 0.463 |
| *Formicarius moniliger* | 49 | qp | 0.8 | 500 | 746.397 | 0.504 | 0.699 | 1.391 | 0.000 | 0.000 | pp_coldest_q, temp_mean_coldest_q, temp_range_diurnal, temp_seasonality | 0.610 |
| *Forpus cyanopygius* | 63 | lq | 0.8 | 500 | 883.359 | 0.000 | 0.725 | 1.340 | 0.000 | 0.133 | Roughness_index, Moisture_index, Temp_max_Warmest | 0.342 |
| *Galbula ruficauda* | 1123 | lqp | 1.0 | 500 | 20996.251 | 14.555 | 0.702 | 1.103 | 0.000 | 0.049 | Roughness_index, Aridity_index, Moisture_index, Temp_max_Coldest | 0.583 |
| *Geococcyx californianus* | 1991 | lqp | 1.0 | 500 | 35355.902 | 7.386 | 0.849 | 1.508 | 0.000 | 0.031 | Roughness_index, Moisture_index, Temp_max_Coldest | 0.303 |
| *Geococcyx velox* | 325 | lqp | 1.0 | 500 | 5172.427 | 0.736 | 0.802 | 1.376 | 0.000 | 0.013 | Moisture_index, Elevation | 0.464 |
| *Glaucis aeneus* | 97 | lq | 0.8 | 500 | 1109.013 | 0.000 | 0.776 | 1.406 | 0.000 | 0.105 | Moisture_index, Temp_max_Coldest | 0.571 |
| *Glyphorynchus spirurus* | 1197 | lqp | 1.2 | 500 | 22510.694 | 3.901 | 0.752 | 1.158 | 0.000 | 0.086 | Roughness_index, Moisture_index, Temp_max_Coldest | 0.463 |
| *Gnorimopsar chopi* | 2544 | lqp | 0.9 | 500 | 46319.853 | 0.115 | 0.799 | 1.261 | 0.000 | 0.063 | Aridity_index, Moisture_index, Temp_max_Warmest, Temp_min_Coldest, Elevation | 0.477 |
| *Grallaria guatimalensis* | 249 | lqp | 1.0 | 500 | 4325.490 | 2.435 | 0.873 | 1.354 | 0.000 | 0.065 | Moisture_index, Temp_max_Coldest | 0.348 |
| *Granatellus sallaei* | 64 | lq | 0.8 | 500 | 910.003 | 0.000 | 0.764 | 1.243 | 0.000 | 0.200 | Aridity_index, Temp_min_Warmest, Temp_min_Coldest | 0.633 |
| *Granatellus venustus* | 124 | lqp | 1.1 | 500 | 1430.342 | 1.472 | 0.775 | 1.223 | 0.000 | 0.208 | Aridity_index, Moisture_index, Temp_min_Warmest | 0.379 |
| *Gymnocichla nudiceps* | 133 | lq | 1.0 | 500 | 1863.996 | 0.067 | 0.843 | 1.483 | 0.000 | 0.000 | Moisture_index, Temp_min_Warmest | 0.417 |
| *Gymnomystax mexicanus* | 533 | qp | 0.8 | 500 | 9745.313 | 0.298 | 0.728 | 1.157 | 0.000 | 0.031 | Slope, Temp_max_Coldest, Temp_min_Warmest, Precipitation | 0.493 |
| *Gymnopithys leucaspis* | 332 | lqp | 1.2 | 500 | 5354.117 | 2.601 | 0.881 | 1.389 | 0.000 | 0.078 | Roughness_index, Moisture_index, Temp_max_Warmest, Temp_min_Coldest | 0.319 |
| *Gymnopithys rufigula* | 169 | lqp | 0.9 | 500 | 2970.299 | 0.603 | 0.781 | 1.243 | 0.000 | 0.048 | Roughness_index, Moisture_index, Temp_max_Coldest, Temp_min_Coldest | 0.433 |
| *Gymnorhinus cyanocephalus* | 1129 | lqp | 1.0 | 500 | 19700.836 | 14.731 | 0.872 | 1.469 | 0.000 | 0.067 | Slope, Temp_max_Coldest, Precipitation, Elevation | 0.299 |
| *Habia fuscicauda* | 267 | lqp | 0.8 | 500 | 4019.141 | 0.000 | 0.742 | 1.298 | 0.000 | 0.066 | Moisture_index, Temp_max_Warmest | 0.582 |
| *Habia rubica* | 1033 | lqp | 0.8 | 500 | 18249.819 | 0.000 | 0.815 | 1.120 | 0.000 | 0.083 | Roughness_index, Aridity_index, Temp_max_Coldest, Temp_min_Warmest, Precipitation | 0.440 |
| *Haplospiza rustica* | 154 | lq | 1.0 | 500 | 2559.607 | 0.145 | 0.924 | 1.447 | 0.000 | 0.053 | Aridity_index, Temp_min_Coldest | 0.161 |
| *Haplospiza unicolor* | 330 | qp | 0.8 | 500 | 4985.807 | 0.000 | 0.946 | 1.650 | 0.000 | 0.038 | Roughness_index, Aridity_index, Precipitation, Temp_max_Warmest, Temp_min_Coldest | 0.200 |
| *Heliomaster constantii* | 338 | lqp | 1.0 | 500 | 4885.714 | 1.927 | 0.758 | 1.213 | 0.000 | 0.068 | Slope, Moisture_index, Temp_min_Warmest | 0.439 |
| *Heliomaster furcifer* | 781 | lqp | 0.8 | 500 | 14355.999 | 0.000 | 0.821 | 1.311 | 0.000 | 0.067 | pp_coldest_q, temp_mean_coldest_q, temp_range_diurnal, temp_seasonality | 0.481 |
| *Heliomaster longirostris* | 806 | lqp | 0.8 | 500 | 14737.087 | 0.000 | 0.723 | 1.082 | 0.000 | 0.063 | Aridity_index, Temp_max_Warmest, Temp_min_Coldest | 0.604 |
| *Heliomaster squamosus* | 330 | qp | 0.9 | 500 | 5690.578 | 9.745 | 0.805 | 1.238 | 0.000 | 0.049 | Roughness_index, Moisture_index, Temp_max_Coldest, Temp_min_Warmest | 0.442 |
| *Heliothryx auritus* | 641 | lqp | 0.9 | 500 | 12140.002 | 2.062 | 0.761 | 1.213 | 0.000 | 0.025 | Roughness_index, Moisture_index, Temp_max_Warmest | 0.481 |
| *Heliothryx barroti* | 262 | lp | 1.0 | 500 | 3930.273 | 0.301 | 0.762 | 1.174 | 0.000 | 0.082 | Moisture_index, Temp_max_Coldest, Temp_max_Warmest | 0.582 |
| *Henicorhina leucophrys* | 540 | lqp | 0.8 | 500 | 8995.391 | 0.000 | 0.885 | 1.401 | 0.000 | 0.045 | Moisture_index, Temp_min_Warmest | 0.265 |
| *Henicorhina leucosticta* | 506 | lqp | 0.9 | 500 | 8692.408 | 1.913 | 0.862 | 1.283 | 0.000 | 0.058 | Roughness_index, Temp_max_Coldest, Temp_max_Warmest, Temp_min_Coldest | 0.307 |
| *Hylocharis eliciae* | 196 | lq | 1.2 | 500 | 2457.888 | 0.255 | 0.806 | 1.280 | 0.000 | 0.040 | Roughness_index, Moisture_index, Temp_max_Coldest | 0.458 |
| *Hylocharis leucotis* | 504 | qp | 0.9 | 500 | 8039.884 | 0.161 | 0.851 | 1.356 | 0.000 | 0.056 | Roughness_index, Moisture_index, Temp_min_Warmest | 0.354 |
| *Hylomanes momotula* | 103 | lqp | 0.8 | 500 | 1583.944 | 5.290 | 0.805 | 1.289 | 0.000 | 0.120 | Slope, Moisture_index, Temp_max_Coldest | 0.506 |
| *Hylopezus dives* | 73 | lq | 0.8 | 500 | 1036.969 | 0.000 | 0.848 | 1.509 | 0.000 | 0.000 | Aridity_index, Temp_max_Coldest, Precipitation | 0.425 |
| *Hylopezus perspicillatus* | 95 | lq | 0.9 | 500 | 1297.848 | 1.066 | 0.772 | 1.253 | 0.000 | 0.143 | Moisture_index, Temp_max_Coldest | 0.493 |
| *Hylophilus decurtatus* | 343 | qp | 0.8 | 500 | 5011.183 | 0.000 | 0.712 | 1.174 | 0.000 | 0.027 | Aridity_index, Elevation | 0.551 |
| *Hylophylax naevioides* | 137 | lq | 0.8 | 500 | 1876.947 | 0.000 | 0.823 | 1.402 | 0.000 | 0.067 | Moisture_index, Temp_max_Warmest | 0.356 |
| *Hylophylax naevius* | 465 | lqp | 0.8 | 500 | 8869.432 | 0.000 | 0.792 | 1.248 | 0.000 | 0.017 | Roughness_index, Aridity_index, Temp_max_Coldest, Temp_max_Warmest | 0.392 |
| *Hylorchilus navai* | 15 | lp | 1.1 | 500 | 211.064 | 0.157 | 0.509 | 1.273 | 0.000 | 0.250 | pp_coldest_q, temp_mean_coldest_q, temp_range_diurnal, temp_seasonality | 0.592 |
| *Hylorchilus sumichrasti* | 22 | lqp | 1.2 | 500 | 306.373 | 0.521 | 0.718 | 1.367 | 0.000 | 0.333 | pp_coldest_q, temp_mean_coldest_q, temp_range_diurnal, temp_seasonality | 0.587 |
| *Hypopyrrhus pyrohypogaster* | 71 | lqp | 0.9 | 500 | 1001.126 | 0.726 | 0.863 | 1.555 | 0.000 | 0.056 | Moisture_index, Elevation | 0.430 |
| *Icterus abeillei* | 163 | lqp | 0.9 | 500 | 2458.071 | 2.818 | 0.916 | 1.676 | 0.000 | 0.000 | Roughness_index, Aridity_index, Temp_max_Coldest, Temp_min_Warmest | 0.292 |
| *Icterus auricapillus* | 305 | lq | 1.2 | 500 | 4733.765 | 1.197 | 0.615 | 1.082 | 0.000 | 0.071 | Roughness_index, Aridity_index, Precipitation | 0.590 |
| *Icterus cayanensis* | 1822 | lqp | 0.8 | 500 | 34717.979 | 0.000 | 0.692 | 1.038 | 0.001 | 0.068 | Moisture_index, Temp_max_Coldest, Elevation | 0.574 |
| *Icterus chrysater* | 485 | lqp | 0.8 | 500 | 7846.411 | 0.000 | 0.818 | 1.154 | 0.056 | 0.017 | Moisture_index, Elevation | 0.380 |
| *Icterus cucullatus* | 1013 | lqp | 0.8 | 500 | 16223.523 | 0.000 | 0.908 | 1.557 | 0.000 | 0.065 | Aridity_index, Temp_max_Coldest, Elevation | 0.300 |
| *Icterus dominicensis* | 210 | lq | 0.8 | 500 | 1932.593 | 0.477 | 0.470 | 1.020 | 0.000 | 0.139 | Temp_max_Coldest, Temp_min_Warmest | 0.667 |
| *Icterus galbula* | 1549 | lqp | 0.9 | 500 | 27936.729 | 2.012 | 0.855 | 1.353 | 0.000 | 0.077 | Moisture_index, Temp_max_Coldest, Elevation | 0.312 |
| *Icterus graceannae* | 89 | lqp | 0.8 | 500 | 958.127 | 0.025 | 0.878 | 1.488 | 0.000 | 0.111 | Moisture_index, Temp_min_Coldest | 0.426 |
| *Icterus graduacauda* | 336 | lqp | 1.2 | 500 | 5354.715 | 8.161 | 0.910 | 1.562 | 0.000 | 0.013 | Roughness_index, Moisture_index, Temp_max_Coldest, Temp_min_Warmest | 0.388 |
| *Icterus gularis* | 346 | lqp | 1.0 | 500 | 4843.926 | 0.316 | 0.727 | 1.245 | 0.000 | 0.068 | Temp_min_Warmest, Temp_min_Coldest | 0.354 |
| *Icterus maculialatus* | 44 | lq | 1.2 | 500 | 620.742 | 0.000 | 0.949 | 1.813 | 0.000 | 0.000 | Moisture_index, Temp_max_Coldest, Temp_min_Warmest | 0.275 |
| *Icterus mesomelas* | 447 | lq | 0.8 | 500 | 6989.317 | 0.000 | 0.746 | 1.313 | 0.000 | 0.029 | Roughness_index, Aridity_index, Temp_min_Coldest | 0.497 |
| *Icterus parisorum* | 1203 | lqp | 1.0 | 500 | 21362.935 | 13.046 | 0.836 | 1.416 | 0.000 | 0.048 | Roughness_index, Moisture_index, Temp_max_Coldest | 0.379 |
| *Icterus pectoralis* | 162 | lqp | 0.8 | 500 | 1847.333 | 0.000 | 0.735 | 1.254 | 0.000 | 0.033 | Moisture_index, Temp_min_Warmest | 0.504 |
| *Icterus prosthemelas* | 193 | lq | 1.0 | 500 | 2634.439 | 0.610 | 0.758 | 1.213 | 0.000 | 0.095 | Aridity_index, Temp_max_Coldest, Temp_min_Warmest | 0.534 |
| *Icterus pustulatus* | 447 | lqp | 1.1 | 500 | 6206.408 | 6.539 | 0.828 | 1.359 | 0.000 | 0.053 | Aridity_index, Temp_min_Warmest, Temp_min_Coldest | 0.440 |
| *Icterus spurius* | 1924 | lqp | 0.8 | 500 | 35928.386 | 0.000 | 0.747 | 1.222 | 0.000 | 0.056 | Temp_min_Warmest, Precipitation | 0.506 |
| *Icterus wagleri* | 462 | lqp | 0.8 | 500 | 7386.215 | 0.030 | 0.722 | 1.271 | 0.000 | 0.055 | Roughness_index, Moisture_index, Temp_max_Warmest | 0.417 |
| *Iridophanes pulcherrimus* | 86 | lq | 1.0 | 500 | 1320.784 | 0.900 | 0.965 | 1.804 | 0.000 | 0.000 | Aridity_index, Temp_min_Warmest | 0.268 |
| *Junco hyemalis* | 2398 | lqp | 0.8 | 500 | 46172.787 | 0.000 | 0.805 | 1.193 | 0.000 | 0.061 | Temp_min_Warmest, Precipitation, Elevation | 0.590 |
| *Junco phaeonotus* | 410 | qp | 0.8 | 500 | 6422.062 | 0.000 | 0.852 | 1.330 | 0.000 | 0.059 | Roughness_index, Moisture_index, Temp_min_Warmest | 0.328 |
| *Klais guimeti* | 255 | lq | 0.9 | 500 | 4177.717 | 0.133 | 0.782 | 1.138 | 0.000 | 0.100 | Slope, Moisture_index | 0.307 |
| *Lagopus lagopus* | 1764 | lqp | 0.8 | 500 | 33262.601 | 0.000 | 0.950 | 1.610 | 0.000 | 0.047 | Aridity_index, Temp_max_Warmest, Temp_min_Coldest, Elevation | 0.250 |
| *Lagopus leucura* | 560 | lqp | 0.8 | 500 | 10066.779 | 0.000 | 0.897 | 1.520 | 0.000 | 0.029 | Temp_max_Warmest, Elevation | 0.208 |
| *Lagopus muta* | 2044 | lqp | 0.8 | 500 | 37029.882 | 0.000 | 0.955 | 1.635 | 0.000 | 0.040 | Moisture_index, Temp_min_Warmest, Temp_min_Coldest, Elevation | 0.223 |
| *Lampornis amethystinus* | 144 | lqp | 1.1 | 500 | 2107.154 | 3.290 | 0.897 | 1.355 | 0.000 | 0.111 | Aridity_index, Moisture_index, Temp_max_Coldest, Temp_min_Warmest | 0.459 |
| *Lampornis clemenciae* | 311 | lqp | 0.8 | 500 | 5184.389 | 0.000 | 0.874 | 1.316 | 0.000 | 0.065 | Slope, Precipitation, Elevation | 0.362 |
| *Lampornis hemileucus* | 37 | lq | 0.8 | 500 | 420.412 | 0.000 | 0.902 | 1.703 | 0.000 | 0.111 | Aridity_index, Temp_max_Warmest | 0.470 |
| *Lampornis sybillae* | 53 | lq | 1.0 | 500 | 733.323 | 6.249 | 0.920 | 1.736 | 0.000 | 0.000 | Roughness_index, Aridity_index, Temp_max_Coldest, Temp_max_Warmest | 0.402 |
| *Lampornis viridipallens* | 58 | lq | 1.1 | 500 | 805.009 | 1.287 | 0.934 | 1.698 | 0.000 | 0.071 | Moisture_index, Temp_max_Coldest, Temp_min_Warmest | 0.137 |
| *Lamprolaima rhami* | 70 | lq | 1.2 | 500 | 1004.809 | 2.166 | 0.967 | 1.790 | 0.000 | 0.000 | Moisture_index, Temp_min_Warmest | 0.476 |
| *Lampropsar tanagrinus* | 224 | lqp | 0.8 | 500 | 4153.615 | 0.000 | 0.766 | 1.248 | 0.000 | 0.054 | pp_coldest_q, temp_mean_coldest_q, temp_range_diurnal, temp_seasonality | 0.439 |
| *Lanio aurantius* | 70 | lq | 1.0 | 500 | 1087.201 | 0.437 | 0.810 | 1.348 | 0.000 | 0.000 | Aridity_index, Temp_max_Coldest, Elevation | 0.584 |
| *Lanio leucothorax* | 51 | qp | 0.8 | 500 | 633.719 | 1.410 | 0.835 | 1.500 | 0.000 | 0.000 | Moisture_index, Elevation | 0.468 |
| *Laniocera hypopyrra* | 391 | lqp | 1.1 | 500 | 7458.464 | 0.478 | 0.743 | 1.258 | 0.000 | 0.042 | Roughness_index, Aridity_index, Temp_max_Coldest, Precipitation | 0.390 |
| *Laniocera rufescens* | 69 | lq | 1.0 | 500 | 1066.515 | 1.171 | 0.763 | 1.474 | 0.000 | 0.000 | Precipitation, Elevation | 0.469 |
| *Lanius ludovicianus* | 2777 | lqp | 0.9 | 500 | 51622.012 | 6.356 | 0.826 | 1.343 | 0.000 | 0.044 | Moisture_index, Temp_min_Coldest, Elevation | 0.443 |
| *Legatus leucophaius* | 1269 | lqp | 0.9 | 500 | 23814.401 | 3.722 | 0.718 | 1.129 | 0.000 | 0.086 | Roughness_index, Temp_min_Warmest, Precipitation | 0.519 |
| *Lepidocolaptes affinis* | 189 | lqp | 1.0 | 500 | 2854.554 | 0.460 | 0.894 | 1.433 | 0.000 | 0.064 | Moisture_index, Temp_min_Warmest | 0.248 |
| *Lepidocolaptes lacrymiger* | 393 | lq | 0.9 | 500 | 6247.504 | 0.337 | 0.949 | 1.751 | 0.000 | 0.020 | Moisture_index, Elevation | 0.206 |
| *Lepidocolaptes leucogaster* | 223 | lqp | 0.9 | 500 | 3317.637 | 0.766 | 0.821 | 1.238 | 0.000 | 0.093 | Roughness_index, Aridity_index, Moisture_index, Temp_max_Coldest | 0.430 |
| *Lepidocolaptes souleyetii* | 568 | lqp | 0.9 | 500 | 9228.835 | 0.405 | 0.750 | 1.199 | 0.000 | 0.039 | Roughness_index, Moisture_index | 0.401 |
| *Leptopogon amaurocephalus* | 1607 | lqp | 0.9 | 500 | 30066.723 | 13.190 | 0.808 | 1.179 | 0.000 | 0.018 | Roughness_index, Aridity_index, Temp_max_Coldest, Precipitation | 0.546 |
| *Leucippus taczanowskii* | 69 | qp | 1.0 | 500 | 1005.982 | 3.121 | 0.913 | 1.608 | 0.000 | 0.038 | Roughness_index, Moisture_index, Temp_max_Coldest | 0.244 |
| *Lipaugus unirufus* | 194 | lq | 0.9 | 500 | 2791.768 | 0.740 | 0.802 | 1.374 | 0.000 | 0.045 | Moisture_index, Temp_min_Warmest | 0.607 |
| *Lophornis helenae* | 86 | lq | 0.8 | 500 | 1286.128 | 0.857 | 0.661 | 1.183 | 0.000 | 0.182 | Moisture_index, Temp_max_Coldest, Elevation | 0.443 |
| *Loxia curvirostra* | 3402 | lqp | 0.9 | 500 | 68997.446 | 14.588 | 0.851 | 1.450 | 0.000 | 0.060 | pp_coldest_q, temp_mean_coldest_q, temp_range_diurnal, temp_seasonality | 0.322 |
| *Loxia leucoptera* | 2223 | lqp | 0.8 | 500 | 44797.472 | 0.000 | 0.806 | 1.256 | 0.000 | 0.046 | Temp_max_Coldest, Temp_max_Warmest, Elevation | 0.618 |
| *Lurocalis rufiventris* | 127 | lqp | 0.9 | 500 | 2002.367 | 1.931 | 0.916 | 1.583 | 0.000 | 0.062 | Aridity_index, Temp_max_Warmest | 0.219 |
| *Lurocalis semitorquatus* | 809 | lqp | 0.8 | 500 | 15120.211 | 0.000 | 0.791 | 1.150 | 0.000 | 0.057 | Moisture_index, Temp_max_Coldest, Elevation | 0.475 |
| *Macroagelaius imthurni* | 14 | lq | 1.1 | 500 | 194.022 | 0.804 | 0.861 | 1.693 | 0.000 | 0.250 | pp_coldest_q, temp_mean_coldest_q, temp_range_diurnal, temp_seasonality | 0.458 |
| *Macroagelaius subalaris* | 20 | lq | 0.8 | 500 | 301.793 | 0.000 | 0.953 | 1.883 | 0.000 | 0.000 | pp_coldest_q, temp_mean_coldest_q, temp_range_diurnal, temp_seasonality | 0.224 |
| *Malacoptila panamensis* | 227 | lq | 0.9 | 500 | 3336.441 | 0.374 | 0.711 | 1.153 | 0.000 | 0.098 | Moisture_index, Temp_min_Warmest | 0.547 |
| *Manacus aurantiacus* | 79 | lq | 0.8 | 500 | 761.847 | 0.000 | 0.648 | 1.176 | 0.000 | 0.250 | Precipitation, Temp_min_Coldest | 0.706 |
| *Manacus candei* | 149 | lqp | 1.0 | 500 | 2067.301 | 0.039 | 0.799 | 1.232 | 0.000 | 0.059 | Aridity_index, Moisture_index, Temp_max_Coldest, Temp_min_Warmest | 0.454 |
| *Megaceryle alcyon* | 2450 | lq | 0.8 | 500 | 45277.204 | 0.000 | 0.847 | 1.407 | 0.000 | 0.046 | Moisture_index, Temp_max_Coldest, Elevation | 0.347 |
| *Megaceryle torquata* | 2013 | lqp | 0.9 | 500 | 36112.611 | 2.598 | 0.669 | 1.103 | 0.000 | 0.045 | Moisture_index, Temp_max_Coldest, Elevation | 0.588 |
| *Megarynchus pitangua* | 1626 | lqp | 0.8 | 500 | 29446.562 | 0.000 | 0.740 | 1.118 | 0.000 | 0.049 | Roughness_index, Moisture_index, Temp_max_Coldest | 0.535 |
| *Melanerpes aurifrons* | 738 | lqp | 1.1 | 500 | 12570.289 | 9.695 | 0.758 | 1.264 | 0.000 | 0.073 | Slope, Moisture_index, Temp_max_Coldest, Temp_max_Warmest | 0.443 |
| *Melanerpes chrysogenys* | 269 | lqp | 1.0 | 500 | 3065.211 | 3.678 | 0.739 | 1.312 | 0.000 | 0.038 | Roughness_index, Aridity_index, Precipitation, Temp_max_Warmest | 0.502 |
| *Melanerpes erythrocephalus* | 1635 | lqp | 0.8 | 500 | 30206.338 | 0.000 | 0.825 | 1.365 | 0.000 | 0.068 | Roughness_index, Precipitation, Temp_min_Coldest | 0.369 |
| *Melanerpes formicivorus* | 1314 | lqp | 1.1 | 500 | 22477.912 | 14.537 | 0.888 | 1.441 | 0.000 | 0.059 | Roughness_index, Moisture_index, Temp_max_Coldest, Elevation | 0.334 |
| *Melanerpes hoffmannii* | 165 | lqp | 1.0 | 500 | 2014.240 | 3.883 | 0.813 | 1.386 | 0.000 | 0.083 | Aridity_index, Temp_min_Warmest, Temp_min_Coldest | 0.397 |
| *Melanerpes hypopolius* | 142 | lq | 0.9 | 500 | 1910.040 | 2.460 | 0.904 | 1.433 | 0.000 | 0.083 | Aridity_index, Moisture_index, Temp_max_Coldest, Elevation | 0.361 |
| *Melanerpes lewis* | 1251 | lqp | 1.2 | 500 | 22760.271 | 95.291 | 0.871 | 1.464 | 0.000 | 0.042 | Temp_min_Warmest, Precipitation, Temp_min_Coldest, Elevation | 0.464 |
| *Melanerpes pucherani* | 338 | lqp | 0.8 | 500 | 4859.833 | 0.458 | 0.717 | 1.128 | 0.000 | 0.093 | Moisture_index, Temp_max_Coldest | 0.536 |
| *Melanerpes uropygialis* | 682 | lqp | 1.2 | 500 | 10290.878 | 35.559 | 0.864 | 1.421 | 0.000 | 0.077 | Slope, Temp_max_Coldest, Precipitation, Elevation | 0.372 |
| *Melanotis caerulescens* | 461 | lqp | 0.8 | 500 | 6991.886 | 0.000 | 0.805 | 1.289 | 0.000 | 0.092 | Slope, Temp_min_Warmest, Temp_min_Coldest | 0.423 |
| *Melanotis hypoleucus* | 122 | lqp | 0.8 | 500 | 1674.511 | 2.537 | 0.907 | 1.405 | 0.000 | 0.100 | Moisture_index, Temp_max_Coldest, Temp_max_Warmest | 0.311 |
| *Meleagris gallopavo* | 2285 | lq | 0.8 | 500 | 44509.655 | 9.819 | 0.751 | 1.212 | 0.000 | 0.051 | Roughness_index, Precipitation, Temp_max_Warmest | 0.622 |
| *Meleagris ocellata* | 217 | lq | 0.8 | 500 | 2703.074 | 0.000 | 0.802 | 1.421 | 0.000 | 0.042 | Roughness_index, Aridity_index, Moisture_index, Temp_min_Warmest, Temp_max_Warmest, Temp_min_Coldest | 0.294 |
| *Melospiza georgiana* | 1723 | lqp | 1.0 | 500 | 30119.008 | 9.887 | 0.917 | 1.567 | 0.000 | 0.037 | Moisture_index, Temp_max_Coldest, Elevation | 0.274 |
| *Melospiza lincolnii* | 2368 | lqp | 1.0 | 500 | 46371.494 | 3.208 | 0.745 | 1.179 | 0.000 | 0.053 | Moisture_index, Temp_max_Coldest, Elevation | 0.579 |
| *Melospiza melodia* | 2445 | lqp | 1.2 | 500 | 45738.864 | 241.578 | 0.827 | 1.303 | 0.000 | 0.033 | Precipitation, Temp_max_Warmest, Elevation | 0.603 |
| *Melozone biarcuata* | 92 | lqp | 0.8 | 500 | 1223.466 | 2.437 | 0.929 | 1.641 | 0.000 | 0.174 | Moisture_index, Temp_max_Coldest, Temp_min_Warmest | 0.278 |
| *Melozone kieneri* | 214 | lqp | 1.0 | 500 | 3164.242 | 1.179 | 0.886 | 1.526 | 0.000 | 0.040 | Slope, Moisture_index, Elevation | 0.415 |
| *Melozone leucotis* | 60 | lq | 0.8 | 500 | 842.998 | 0.000 | 0.968 | 1.867 | 0.000 | 0.000 | pp_coldest_q, temp_mean_coldest_q, temp_range_diurnal, temp_seasonality | 0.351 |
| *Microcerculus philomela* | 77 | lq | 1.2 | 500 | 1086.722 | 1.495 | 0.805 | 1.309 | 0.000 | 0.158 | Moisture_index, Temp_max_Coldest, Elevation | 0.310 |
| *Microchera albocoronata* | 45 | lqp | 1.2 | 500 | 593.963 | 0.206 | 0.828 | 1.484 | 0.000 | 0.000 | pp_coldest_q, temp_mean_coldest_q, temp_range_diurnal, temp_seasonality | 0.611 |
| *Microrhopias quixensis* | 539 | lqp | 1.1 | 500 | 9724.477 | 0.986 | 0.768 | 1.210 | 0.000 | 0.039 | Roughness_index, Moisture_index, Temp_max_Coldest | 0.337 |
| *Mimus gilvus* | 816 | lqp | 0.9 | 500 | 13481.591 | 0.845 | 0.647 | 1.080 | 0.000 | 0.073 | Aridity_index, Elevation | 0.711 |
| *Mimus polyglottos* | 2685 | lqp | 1.0 | 500 | 47408.325 | 12.912 | 0.852 | 1.452 | 0.000 | 0.044 | Moisture_index, Temp_max_Coldest, Elevation | 0.319 |
| *Mionectes oleagineus* | 870 | lq | 0.9 | 500 | 15764.388 | 7.508 | 0.808 | 1.194 | 0.000 | 0.049 | Roughness_index, Moisture_index, Temp_max_Coldest | 0.463 |
| *Mitrephanes olivaceus* | 34 | lq | 1.2 | 500 | 543.174 | 2.505 | 0.785 | 1.471 | 0.000 | 0.375 | Slope, Moisture_index | 0.174 |
| *Mitrephanes phaeocercus* | 431 | lqp | 0.8 | 500 | 7110.156 | 1.010 | 0.782 | 1.182 | 0.000 | 0.048 | Roughness_index, Moisture_index, Temp_min_Warmest | 0.416 |
| *Molothrus aeneus* | 1082 | lqp | 0.8 | 500 | 17953.579 | 0.000 | 0.797 | 1.417 | 0.000 | 0.024 | Roughness_index, Moisture_index, Temp_max_Coldest, Temp_max_Warmest | 0.357 |
| *Molothrus ater* | 3064 | lqp | 0.8 | 500 | 59433.119 | 0.000 | 0.784 | 1.301 | 0.000 | 0.060 | Moisture_index, Temp_min_Coldest, Elevation | 0.410 |
| *Molothrus bonariensis* | 2022 | lqp | 0.8 | 500 | 37030.354 | 0.000 | 0.741 | 1.089 | 0.000 | 0.069 | Moisture_index, Temp_max_Coldest, Elevation | 0.553 |
| *Molothrus oryzivorus* | 1943 | qp | 0.8 | 500 | 36584.560 | 10.653 | 0.714 | 1.158 | 0.000 | 0.041 | Roughness_index, Aridity_index, Temp_max_Warmest, Temp_min_Coldest | 0.504 |
| *Molothrus rufoaxillaris* | 1911 | lqp | 1.1 | 500 | 34712.566 | 27.684 | 0.834 | 1.303 | 0.000 | 0.056 | Moisture_index, Temp_min_Coldest, Elevation | 0.397 |
| *Momotus mexicanus* | 321 | lqp | 1.0 | 500 | 4553.361 | 3.198 | 0.848 | 1.429 | 0.000 | 0.042 | Roughness_index, Aridity_index, Moisture_index, Temp_max_Coldest, Temp_max_Warmest | 0.356 |
| *Momotus momota* | 1229 | lqp | 1.1 | 500 | 24287.567 | 5.056 | 0.625 | 1.114 | 0.000 | 0.072 | Roughness_index, Moisture_index, Temp_max_Warmest | 0.440 |
| *Monasa morphoeus* | 747 | lqp | 0.8 | 500 | 14153.002 | 0.000 | 0.721 | 1.200 | 0.000 | 0.049 | Slope, Aridity_index, Temp_max_Warmest, Temp_min_Coldest | 0.491 |
| *Morococcyx erythropygus* | 264 | lqp | 1.2 | 500 | 3344.089 | 6.441 | 0.815 | 1.374 | 0.000 | 0.037 | Slope, Moisture_index, Temp_max_Coldest, Temp_max_Warmest | 0.409 |
| *Myadestes occidentalis* | 432 | lq | 1.2 | 500 | 6879.457 | 1.099 | 0.824 | 1.199 | 0.000 | 0.093 | Roughness_index, Moisture_index, Temp_min_Warmest | 0.276 |
| *Myadestes townsendi* | 1844 | lqp | 0.9 | 500 | 34440.082 | 2.579 | 0.872 | 1.381 | 0.000 | 0.046 | Roughness_index, Moisture_index, Temp_max_Coldest | 0.433 |
| *Myadestes unicolor* | 99 | lq | 1.2 | 500 | 1518.962 | 3.902 | 0.858 | 1.482 | 0.000 | 0.000 | Roughness_index, Temp_max_Coldest | 0.546 |
| *Myiarchus cinerascens* | 2147 | lq | 0.8 | 500 | 38633.829 | 47.864 | 0.802 | 1.325 | 0.000 | 0.060 | Roughness_index, Moisture_index, Temp_max_Coldest | 0.409 |
| *Myiarchus crinitus* | 1774 | lq | 0.8 | 500 | 32150.414 | 0.000 | 0.829 | 1.309 | 0.000 | 0.057 | Temp_max_Warmest, Elevation | 0.378 |
| *Myiarchus nuttingi* | 438 | lqp | 0.8 | 500 | 6360.696 | 0.714 | 0.777 | 1.277 | 0.000 | 0.074 | Roughness_index, Temp_min_Warmest, Precipitation | 0.383 |
| *Myiarchus tuberculifer* | 1257 | lqp | 0.9 | 500 | 23290.484 | 2.139 | 0.739 | 1.084 | 0.000 | 0.078 | Roughness_index, Moisture_index, Temp_max_Warmest | 0.427 |
| *Myiarchus tyrannulus* | 1703 | lqp | 1.0 | 500 | 32344.612 | 0.737 | 0.715 | 1.113 | 0.000 | 0.053 | Moisture_index, Temp_min_Warmest | 0.437 |
| *Myiarchus yucatanensis* | 331 | lq | 0.9 | 500 | 3940.612 | 1.401 | 0.807 | 1.362 | 0.000 | 0.070 | Aridity_index, Temp_max_Coldest, Precipitation, Elevation | 0.341 |
| *Myiobius atricaudus* | 379 | lqp | 0.8 | 500 | 6703.616 | 0.000 | 0.805 | 1.163 | 0.000 | 0.034 | Slope, Moisture_index, Temp_max_Warmest | 0.459 |
| *Myiobius villosus* | 103 | lq | 1.2 | 500 | 1656.536 | 2.245 | 0.877 | 1.327 | 0.000 | 0.154 | Temp_min_Warmest, Precipitation | 0.229 |
| *Myiodynastes luteiventris* | 536 | lq | 1.1 | 500 | 8553.874 | 0.997 | 0.730 | 1.224 | 0.000 | 0.065 | Moisture_index, Elevation | 0.435 |
| *Myiodynastes maculatus* | 1721 | lqp | 0.8 | 500 | 32407.205 | 0.000 | 0.697 | 1.078 | 0.000 | 0.052 | Moisture_index, Temp_max_Coldest, Elevation | 0.592 |
| *Myiopagis viridicata* | 1367 | lqp | 0.9 | 500 | 25774.722 | 7.246 | 0.771 | 1.208 | 0.000 | 0.046 | Roughness_index, Moisture_index, Temp_max_Coldest | 0.516 |
| *Myiopsitta monachus* | 1450 | lq | 0.8 | 500 | 26608.354 | 39.164 | 0.756 | 1.156 | 0.000 | 0.060 | pp_coldest_q, temp_mean_coldest_q, temp_range_diurnal, temp_seasonality | 0.577 |
| *Myiozetetes granadensis* | 533 | lqp | 0.8 | 500 | 9307.429 | 0.000 | 0.806 | 1.281 | 0.000 | 0.032 | Roughness_index, Moisture_index, Temp_max_Coldest | 0.532 |
| *Myiozetetes similis* | 1373 | lq | 0.8 | 500 | 24126.529 | 0.000 | 0.724 | 1.108 | 0.000 | 0.040 | Roughness_index, Moisture_index, Temp_min_Warmest | 0.569 |
| *Myrmeciza exsul* | 64 | lq | 1.2 | 500 | 926.374 | 0.042 | 0.591 | 1.176 | 0.000 | 0.200 | Aridity_index, Temp_max_Coldest | 0.603 |
| *Myrmotherula axillaris* | 966 | lqp | 0.9 | 500 | 17909.889 | 1.398 | 0.721 | 1.158 | 0.000 | 0.030 | Roughness_index, Aridity_index, Temp_max_Coldest, Temp_min_Coldest | 0.623 |
| *Myrmotherula schisticolor* | 289 | lqp | 1.1 | 500 | 4806.867 | 5.116 | 0.871 | 1.293 | 0.000 | 0.043 | Moisture_index, Temp_max_Warmest | 0.553 |
| *Neomorphus geoffroyi* | 73 | lq | 1.0 | 500 | 1306.423 | 4.254 | 0.803 | 1.277 | 0.000 | 0.111 | Roughness_index, Moisture_index, Temp_max_Coldest | 0.366 |
| *Neomorphus pucheranii* | 11 | qp | 1.1 | 500 | 192.341 | 3.132 | 0.840 | 1.667 | 0.000 | 0.333 | Roughness_index, Aridity_index, Temp_max_Coldest, Temp_min_Warmest | 0.709 |
| *Neomorphus radiolosus* | 17 | lq | 1.1 | 500 | 250.402 | 0.251 | 0.710 | 1.502 | 0.000 | 0.000 | pp_coldest_q, temp_mean_coldest_q, temp_range_diurnal, temp_seasonality | 0.561 |
| *Neomorphus rufipennis* | 28 | lq | 0.8 | 500 | 494.277 | 0.000 | 0.617 | 1.271 | 0.000 | 0.286 | pp_coldest_q, temp_mean_coldest_q, temp_range_diurnal, temp_seasonality | 0.548 |
| *Notharchus hyperrhynchus* | 509 | lp | 1.2 | 500 | 9001.867 | 1.284 | 0.650 | 1.085 | 0.000 | 0.044 | Aridity_index, Temp_max_Warmest, Elevation | 0.629 |
| *Nucifraga caryocatactes* | 1904 | lq | 0.8 | 500 | 34741.809 | 28.969 | 0.954 | 1.649 | 0.000 | 0.066 | Temp_max_Warmest, Temp_min_Coldest, Elevation | 0.412 |
| *Nucifraga columbiana* | 1431 | lqp | 0.9 | 500 | 25960.608 | 4.577 | 0.877 | 1.481 | 0.000 | 0.047 | Roughness_index, Temp_max_Coldest, Precipitation | 0.353 |
| *Nyctibius grandis* | 755 | qp | 0.8 | 500 | 14437.392 | 2.218 | 0.685 | 1.137 | 0.000 | 0.076 | Slope, Aridity_index, Temp_max_Warmest, Temp_min_Coldest | 0.455 |
| *Nyctibius griseus* | 1903 | lqp | 0.8 | 500 | 36496.972 | 0.000 | 0.688 | 1.095 | 0.000 | 0.046 | Roughness_index, Aridity_index, Temp_max_Coldest | 0.582 |
| *Nyctibius jamaicensis* | 229 | lqp | 0.9 | 500 | 3019.012 | 0.365 | 0.753 | 1.330 | 0.000 | 0.022 | Slope, Aridity_index, Temp_max_Warmest, Temp_min_Coldest | 0.437 |
| *Nyctidromus albicollis* | 1699 | lqp | 0.8 | 500 | 31572.770 | 0.000 | 0.686 | 1.080 | 0.000 | 0.054 | Moisture_index, Temp_max_Coldest, Elevation | 0.591 |
| *Nyctiphrynus mcleodii* | 52 | lq | 1.2 | 500 | 751.845 | 2.524 | 0.823 | 1.542 | 0.000 | 0.000 | pp_coldest_q, temp_mean_coldest_q, temp_range_diurnal, temp_seasonality | 0.282 |
| *Nyctiphrynus yucatanicus* | 68 | lq | 0.8 | 500 | 824.283 | 1.237 | 0.786 | 1.375 | 0.000 | 0.200 | Aridity_index, Temp_max_Coldest, Precipitation, Elevation | 0.432 |
| *Odontophorus guttatus* | 106 | lq | 0.8 | 500 | 1639.910 | 0.000 | 0.775 | 1.183 | 0.000 | 0.154 | Roughness_index, Aridity_index, Temp_min_Coldest | 0.352 |
| *Odontophorus melanotis* | 43 | lqp | 0.8 | 500 | 621.086 | 2.037 | 0.849 | 1.514 | 0.000 | 0.000 | pp_coldest_q, temp_mean_coldest_q, temp_range_diurnal, temp_seasonality | 0.443 |
| *Oncostoma cinereigulare* | 233 | lqp | 1.0 | 500 | 3437.013 | 1.158 | 0.707 | 1.208 | 0.000 | 0.111 | Aridity_index, Moisture_index, Temp_max_Coldest, Temp_min_Warmest | 0.460 |
| *Oncostoma olivaceum* | 115 | lq | 1.1 | 500 | 1566.428 | 0.066 | 0.789 | 1.253 | 0.000 | 0.077 | Moisture_index, Temp_min_Coldest | 0.410 |
| *Onychorhynchus mexicanus* | 53 | lqp | 1.1 | 500 | 905.334 | 1.148 | 0.544 | 1.106 | 0.000 | 0.077 | pp_coldest_q, temp_mean_coldest_q, temp_range_diurnal, temp_seasonality | 0.700 |
| *Oreophasis derbianus* | 33 | qp | 0.8 | 500 | 453.862 | 0.000 | 0.823 | 1.423 | 0.000 | 0.250 | pp_coldest_q, temp_mean_coldest_q, temp_range_diurnal, temp_seasonality | 0.246 |
| *Oreopsar bolivianus* | 52 | lq | 1.2 | 500 | 797.314 | 6.121 | 0.755 | 1.237 | 0.000 | 0.385 | pp_coldest_q, temp_mean_coldest_q, temp_range_diurnal, temp_seasonality | 0.569 |
| *Oriturus superciliosus* | 157 | qp | 1.1 | 500 | 2328.396 | 0.284 | 0.874 | 1.395 | 0.000 | 0.051 | Roughness_index, Aridity_index, Moisture_index, Temp_min_Warmest | 0.263 |
| *Ornithion semiflavum* | 117 | lqp | 0.8 | 500 | 1672.650 | 0.000 | 0.788 | 1.241 | 0.000 | 0.074 | Moisture_index, Temp_max_Coldest, Elevation | 0.427 |
| *Ortalis cinereiceps* | 163 | lq | 1.1 | 500 | 2003.683 | 0.478 | 0.758 | 1.247 | 0.000 | 0.061 | Slope, Aridity_index, Temp_min_Coldest | 0.515 |
| *Ortalis leucogastra* | 59 | lq | 0.9 | 500 | 521.649 | 0.188 | 0.868 | 1.561 | 0.000 | 0.100 | pp_coldest_q, temp_mean_coldest_q, temp_range_diurnal, temp_seasonality | 0.475 |
| *Ortalis poliocephala* | 209 | lqp | 1.2 | 500 | 2557.126 | 6.625 | 0.839 | 1.522 | 0.000 | 0.048 | Roughness_index, Aridity_index, Temp_max_Coldest, Temp_max_Warmest | 0.272 |
| *Ortalis vetula* | 276 | lqp | 0.9 | 500 | 4153.583 | 0.549 | 0.734 | 1.269 | 0.000 | 0.048 | Temp_min_Warmest, Temp_min_Coldest | 0.390 |
| *Ortalis wagleri* | 123 | lqp | 0.8 | 500 | 1484.693 | 0.355 | 0.849 | 1.390 | 0.000 | 0.154 | Roughness_index, Moisture_index, Temp_max_Coldest, Temp_max_Warmest | 0.469 |
| *Pachyramphus aglaiae* | 549 | lqp | 0.8 | 500 | 8576.479 | 0.000 | 0.740 | 1.290 | 0.000 | 0.073 | Moisture_index, Elevation | 0.314 |
| *Pachyramphus albogriseus* | 224 | lqp | 1.0 | 500 | 3759.674 | 1.836 | 0.874 | 1.446 | 0.000 | 0.054 | Moisture_index, Temp_max_Warmest | 0.292 |
| *Pachyramphus cinnamomeus* | 465 | lq | 0.9 | 500 | 7668.747 | 0.564 | 0.697 | 1.063 | 0.000 | 0.037 | Roughness_index, Precipitation | 0.553 |
| *Pachyramphus homochrous* | 149 | lq | 0.8 | 500 | 2195.856 | 0.000 | 0.720 | 1.142 | 0.000 | 0.086 | Aridity_index, Temp_max_Warmest | 0.632 |
| *Pachyramphus major* | 139 | qp | 1.2 | 500 | 2173.345 | 0.239 | 0.757 | 1.313 | 0.000 | 0.059 | Slope, Aridity_index, Temp_max_Coldest | 0.420 |
| *Pachyramphus polychopterus* | 1486 | lqp | 0.9 | 500 | 28284.046 | 4.866 | 0.708 | 1.054 | 0.000 | 0.059 | Roughness_index, Moisture_index, Temp_max_Coldest | 0.516 |
| *Panterpe insignis* | 44 | lq | 1.2 | 500 | 446.955 | 1.203 | 0.931 | 1.760 | 0.000 | 0.091 | Moisture_index, Elevation | 0.165 |
| *Panyptila cayennensis* | 732 | lqp | 0.8 | 500 | 12923.964 | 0.000 | 0.751 | 1.112 | 0.015 | 0.084 | Roughness_index, Moisture_index, Temp_max_Coldest | 0.486 |
| *Panyptila sanctihieronymi* | 101 | lq | 0.9 | 500 | 1490.973 | 0.226 | 0.896 | 1.432 | 0.000 | 0.083 | Roughness_index, Aridity_index, Temp_max_Warmest, Temp_min_Coldest | 0.467 |
| *Parus atricapillus* | 1979 | lqp | 0.8 | 500 | 36353.598 | 0.000 | 0.865 | 1.382 | 0.000 | 0.062 | Moisture_index, Temp_max_Coldest, Elevation | 0.434 |
| *Parus carolinensis* | 1144 | lqp | 1.1 | 500 | 19761.394 | 11.717 | 0.791 | 1.366 | 0.000 | 0.083 | Slope, Precipitation, Temp_min_Coldest | 0.358 |
| *Parus gambeli* | 1671 | lqp | 0.8 | 500 | 30967.815 | 0.000 | 0.831 | 1.189 | 0.000 | 0.060 | Precipitation, Temp_max_Warmest, Elevation | 0.363 |
| *Parus sclateri* | 234 | lqp | 1.1 | 500 | 3642.534 | 0.613 | 0.869 | 1.459 | 0.000 | 0.017 | Roughness_index, Moisture_index, Elevation | 0.471 |
| *Passer domesticus* | 3438 | lqp | 0.9 | 500 | 63295.550 | 13.439 | 0.928 | 1.535 | 0.000 | 0.056 | Moisture_index, Temp_max_Coldest, Elevation | 0.256 |
| *Passerculus sandwichensis* | 2808 | lqp | 0.9 | 500 | 53832.773 | 2.523 | 0.772 | 1.185 | 0.000 | 0.068 | Moisture_index, Temp_max_Coldest, Elevation | 0.529 |
| *Passerina amoena* | 1950 | lq | 0.8 | 500 | 35688.586 | 0.465 | 0.829 | 1.252 | 0.000 | 0.054 | Roughness_index, Temp_max_Warmest | 0.392 |
| *Passerina caerulea* | 2318 | lqp | 1.0 | 500 | 43655.435 | 1.626 | 0.752 | 1.253 | 0.000 | 0.037 | Moisture_index, Temp_max_Coldest, Elevation | 0.479 |
| *Passerina ciris* | 824 | lqp | 0.8 | 500 | 13771.770 | 0.000 | 0.851 | 1.350 | 0.000 | 0.096 | Roughness_index, Moisture_index, Temp_max_Coldest, Temp_min_Warmest | 0.382 |
| *Passerina cyanea* | 1775 | lqp | 0.8 | 500 | 32744.592 | 0.000 | 0.855 | 1.471 | 0.000 | 0.049 | Roughness_index, Moisture_index, Temp_min_Warmest | 0.358 |
| *Passerina leclancherii* | 178 | lqp | 0.8 | 500 | 1942.668 | 6.235 | 0.785 | 1.206 | 0.000 | 0.061 | Roughness_index, Aridity_index, Precipitation, Temp_max_Warmest | 0.620 |
| *Passerina rositae* | 26 | lqp | 0.9 | 500 | 395.070 | 0.000 | 0.816 | 1.594 | 0.000 | 0.000 | pp_coldest_q, temp_mean_coldest_q, temp_range_diurnal, temp_seasonality | 0.490 |
| *Passerina versicolor* | 805 | lqp | 0.8 | 500 | 13402.778 | 0.000 | 0.801 | 1.255 | 0.000 | 0.052 | Roughness_index, Moisture_index, Temp_min_Warmest, Temp_min_Coldest | 0.564 |
| *Penelope purpurascens* | 428 | lqp | 1.0 | 500 | 6585.207 | 2.372 | 0.779 | 1.242 | 0.000 | 0.021 | Roughness_index, Moisture_index, Temp_max_Coldest | 0.540 |
| *Penelopina nigra* | 71 | lqp | 1.0 | 500 | 1004.634 | 1.273 | 0.843 | 1.315 | 0.000 | 0.111 | Roughness_index, Moisture_index, Temp_max_Coldest, Temp_max_Warmest | 0.292 |
| *Periporphyrus erythromelas* | 39 | lp | 0.9 | 500 | 671.764 | 0.898 | 0.765 | 1.332 | 0.000 | 0.100 | pp_coldest_q, temp_mean_coldest_q, temp_range_diurnal, temp_seasonality | 0.537 |
| *Peucedramus taeniatus* | 468 | qp | 0.8 | 500 | 7653.390 | 2.170 | 0.918 | 1.537 | 0.000 | 0.034 | Slope, Moisture_index, Temp_max_Coldest, Temp_max_Warmest | 0.258 |
| *Phaenostictus mcleannani* | 88 | lq | 0.9 | 500 | 1180.623 | 1.638 | 0.777 | 1.353 | 0.000 | 0.200 | Moisture_index, Temp_max_Warmest | 0.365 |
| *Phaeochroa cuvierii* | 205 | lqp | 0.8 | 500 | 2673.608 | 0.000 | 0.786 | 1.303 | 0.000 | 0.095 | Moisture_index, Temp_max_Coldest | 0.634 |
| *Phaethornis longirostris* | 343 | lqp | 0.8 | 500 | 4849.261 | 0.004 | 0.763 | 1.209 | 0.000 | 0.040 | Moisture_index, Temp_min_Warmest | 0.631 |
| *Phaethornis striigularis* | 475 | lqp | 0.8 | 500 | 7232.323 | 0.000 | 0.738 | 1.155 | 0.000 | 0.075 | Moisture_index, Elevation | 0.469 |
| *Phainopepla nitens* | 1247 | lqp | 0.8 | 500 | 20688.234 | 0.000 | 0.885 | 1.567 | 0.000 | 0.054 | Roughness_index, Moisture_index, Temp_max_Coldest, Elevation | 0.305 |
| *Phalaenoptilus nuttallii* | 1846 | lqp | 0.8 | 500 | 33927.590 | 0.000 | 0.803 | 1.313 | 0.000 | 0.033 | Roughness_index, Temp_max_Coldest, Precipitation, Elevation | 0.447 |
| *Pharomachrus antisianus* | 133 | lq | 0.8 | 500 | 2090.252 | 0.000 | 0.947 | 1.757 | 0.000 | 0.091 | Precipitation, Elevation | 0.108 |
| *Pharomachrus auriceps* | 254 | lqp | 0.9 | 500 | 3993.569 | 1.183 | 0.943 | 1.660 | 0.000 | 0.078 | Precipitation, Temp_max_Warmest | 0.205 |
| *Pharomachrus fulgidus* | 28 | lq | 0.8 | 500 | 370.702 | 0.000 | 0.958 | 1.859 | 0.000 | 0.000 | Roughness_index, Moisture_index, Temp_max_Coldest | 0.119 |
| *Pharomachrus mocinno* | 89 | lq | 1.0 | 500 | 1242.139 | 1.692 | 0.888 | 1.385 | 0.000 | 0.136 | Temp_max_Coldest, Temp_min_Warmest, Precipitation | 0.369 |
| *Pharomachrus pavoninus* | 167 | qp | 1.2 | 500 | 3088.975 | 0.091 | 0.752 | 1.258 | 0.000 | 0.024 | Aridity_index, Temp_max_Coldest, Temp_min_Warmest | 0.521 |
| *Phasianus colchicus* | 1454 | lqp | 1.2 | 500 | 27013.260 | 9.056 | 0.791 | 1.216 | 0.000 | 0.062 | pp_coldest_q, temp_mean_coldest_q, temp_range_diurnal, temp_seasonality | 0.340 |
| *Pheucticus chrysopeplus* | 193 | lqp | 0.9 | 500 | 2777.593 | 0.659 | 0.747 | 1.233 | 0.000 | 0.136 | Moisture_index, Temp_min_Warmest | 0.390 |
| *Pheucticus melanocephalus* | 2288 | lqp | 0.8 | 500 | 42457.615 | 0.000 | 0.791 | 1.223 | 0.000 | 0.059 | Roughness_index, Moisture_index, Temp_max_Coldest | 0.528 |
| *Philortyx fasciatus* | 92 | qp | 0.8 | 500 | 1255.357 | 0.428 | 0.795 | 1.283 | 0.000 | 0.091 | Roughness_index, Aridity_index, Moisture_index, Temp_max_Coldest | 0.574 |
| *Piaya cayana* | 2055 | lqp | 0.8 | 500 | 38463.473 | 0.000 | 0.724 | 1.056 | 0.000 | 0.054 | Roughness_index, Aridity_index, Temp_max_Warmest, Temp_min_Coldest | 0.501 |
| *Pica hudsonia* | 1983 | lqp | 0.8 | 500 | 37725.071 | 0.000 | 0.860 | 1.458 | 0.000 | 0.049 | Roughness_index, Moisture_index, Temp_max_Coldest | 0.345 |
| *Pica nuttalli* | 286 | lqp | 0.8 | 500 | 3767.121 | 0.000 | 0.968 | 1.855 | 0.000 | 0.086 | Roughness_index, Moisture_index, Temp_min_Coldest | 0.171 |
| *Picoides albolarvatus* | 181 | lq | 0.8 | 500 | 9215.913 | 25.485 | 0.869 | 1.421 | 0.000 | 0.126 | Roughness_index, Aridity_index, Elevation | 0.361 |
| *Picoides arizonae* | 45 | lqp | 0.8 | 500 | 2244.586 | 0.000 | 0.867 | 1.460 | 0.000 | 0.390 | Roughness_index, Aridity_index, Moisture_index, Temp_max_Warmest | 0.479 |
| *Picoides fumigatus* | 648 | lqp | 0.8 | 500 | 11274.980 | 0.000 | 0.824 | 1.168 | 0.000 | 0.076 | Moisture_index, Temp_min_Warmest | 0.465 |
| *Picoides pubescens* | 3100 | lqp | 0.9 | 500 | 58024.141 | 44.838 | 0.853 | 1.369 | 0.000 | 0.064 | pp_coldest_q, temp_mean_coldest_q, temp_range_diurnal, temp_seasonality | 0.477 |
| *Picoides scalaris* | 841 | lqp | 0.8 | 500 | 33646.781 | 0.000 | 0.795 | 1.416 | 0.000 | 0.076 | Roughness_index, Moisture_index, Temp_max_Coldest | 0.435 |
| *Picoides tridactylus* | 2162 | lqp | 0.8 | 500 | 40785.425 | 0.000 | 0.965 | 1.835 | 0.000 | 0.038 | Aridity_index, Temp_max_Coldest, Temp_max_Warmest, Elevation | 0.436 |
| *Picoides villosus* | 5474 | lqp | 1.2 | 500 | 45867.460 | 28.369 | 0.818 | 1.276 | 0.000 | 0.061 | Roughness_index, Moisture_index, Temp_max_Coldest | 0.400 |
| *Piculus simplex* | 90 | lq | 1.2 | 500 | 1146.410 | 0.611 | 0.758 | 1.239 | 0.000 | 0.000 | Moisture_index, Elevation | 0.623 |
| *Picumnus olivaceus* | 354 | lqp | 0.9 | 500 | 5498.452 | 0.141 | 0.722 | 1.136 | 0.000 | 0.085 | Moisture_index, Elevation | 0.657 |
| *Pinicola enucleator* | 1974 | lqp | 0.8 | 500 | 38815.014 | 0.000 | 0.884 | 1.375 | 0.000 | 0.045 | Temp_max_Coldest, Temp_max_Warmest, Elevation | 0.446 |
| *Pinicola subhimachala* | 54 | lqp | 1.0 | 500 | 888.824 | 0.125 | 0.817 | 1.295 | 0.000 | 0.214 | Roughness_index, Aridity_index, Moisture_index | 0.292 |
| *Pionus chalcopterus* | 228 | lqp | 0.8 | 500 | 3508.870 | 0.000 | 0.894 | 1.521 | 0.000 | 0.054 | Moisture_index, Temp_max_Warmest | 0.239 |
| *Pionus senilis* | 241 | lq | 0.8 | 500 | 3511.104 | 1.924 | 0.762 | 1.269 | 0.000 | 0.037 | Roughness_index, Aridity_index, Moisture_index, Temp_max_Coldest | 0.375 |
| *Pipilo aberti* | 267 | lqp | 1.0 | 500 | 4224.283 | 5.954 | 0.936 | 1.711 | 0.000 | 0.045 | Roughness_index, Temp_max_Coldest, Elevation | 0.293 |
| *Pipilo albicollis* | 85 | lq | 1.1 | 500 | 1103.828 | 3.393 | 0.964 | 1.831 | 0.000 | 0.000 | Aridity_index, Temp_min_Warmest, Temp_min_Coldest | 0.190 |
| *Pipilo chlorurus* | 1399 | lqp | 1.0 | 500 | 24837.827 | 24.743 | 0.844 | 1.358 | 0.000 | 0.043 | Slope, Temp_max_Coldest, Precipitation, Elevation | 0.438 |
| *Pipilo fuscus* | 1164 | lqp | 0.8 | 500 | 20679.118 | 0.000 | 0.843 | 1.450 | 0.000 | 0.052 | Roughness_index, Temp_max_Coldest, Precipitation, Elevation | 0.437 |
| *Pipilo maculatus* | 1912 | lqp | 1.1 | 500 | 34542.346 | 25.275 | 0.825 | 1.271 | 0.000 | 0.052 | Roughness_index, Moisture_index, Temp_max_Coldest | 0.468 |
| *Pipilo ocai* | 87 | lqp | 0.8 | 500 | 1279.164 | 0.000 | 0.877 | 1.372 | 0.000 | 0.091 | Roughness_index, Aridity_index, Moisture_index, Temp_max_Coldest | 0.484 |
| *Piprites griseiceps* | 19 | lq | 0.8 | 500 | 244.261 | 1.490 | 0.915 | 1.799 | 0.000 | 0.000 | Slope, Moisture_index, Temp_max_Coldest | 0.686 |
| *Piranga bidentata* | 324 | lqp | 0.9 | 500 | 5135.313 | 1.726 | 0.882 | 1.453 | 0.000 | 0.038 | Slope, Moisture_index, Elevation | 0.347 |
| *Piranga erythrocephala* | 139 | lq | 0.8 | 500 | 1986.415 | 0.000 | 0.812 | 1.319 | 0.000 | 0.062 | Roughness_index, Aridity_index, Precipitation, Temp_max_Warmest | 0.398 |
| *Piranga hepatica* | 48 | lq | 0.8 | 500 | 867.443 | 0.000 | 0.843 | 1.420 | 0.000 | 0.083 | Moisture_index, Temp_min_Warmest | 0.425 |
| *Piranga leucoptera* | 349 | lqp | 1.0 | 500 | 6117.527 | 1.194 | 0.838 | 1.386 | 0.000 | 0.034 | Aridity_index, Temp_min_Coldest | 0.463 |
| *Piranga ludoviciana* | 2108 | lqp | 1.2 | 500 | 38650.057 | 14.775 | 0.891 | 1.386 | 0.000 | 0.053 | Roughness_index, Moisture_index, Temp_max_Coldest | 0.444 |
| *Piranga rubra* | 1414 | lqp | 1.2 | 500 | 25595.627 | 8.830 | 0.822 | 1.387 | 0.000 | 0.041 | Aridity_index, Temp_max_Coldest | 0.387 |
| *Pitangus lictor* | 1268 | lqp | 0.8 | 500 | 23861.030 | 0.000 | 0.661 | 1.083 | 0.000 | 0.046 | Moisture_index, Temp_max_Coldest, Temp_min_Warmest | 0.615 |
| *Pitangus sulphuratus* | 2132 | lqp | 0.8 | 500 | 38755.030 | 0.000 | 0.704 | 1.101 | 0.000 | 0.036 | Moisture_index, Temp_max_Coldest, Elevation | 0.586 |
| *Pithys albifrons* | 240 | lqp | 1.0 | 500 | 4311.512 | 6.248 | 0.836 | 1.386 | 0.000 | 0.050 | Roughness_index, Aridity_index, Temp_max_Coldest, Temp_min_Coldest | 0.451 |
| *Platyrinchus cancrominus* | 133 | qp | 0.8 | 500 | 1930.790 | 1.808 | 0.718 | 1.176 | 0.000 | 0.133 | Slope, Aridity_index, Moisture_index, Temp_max_Coldest | 0.323 |
| *Platyrinchus coronatus* | 299 | lp | 0.8 | 500 | 5303.182 | 0.000 | 0.763 | 1.183 | 0.000 | 0.014 | Roughness_index, Moisture_index, Temp_max_Coldest, Temp_min_Coldest | 0.579 |
| *Poecilotriccus sylvia* | 523 | lqp | 1.0 | 500 | 9229.683 | 0.000 | 0.765 | 1.243 | 0.000 | 0.056 | Slope, Moisture_index, Temp_max_Coldest, Temp_min_Coldest | 0.468 |
| *Polioptila albiloris* | 192 | lqp | 0.9 | 500 | 2485.606 | 0.897 | 0.784 | 1.196 | 0.000 | 0.077 | Slope, Aridity_index, Moisture_index, Temp_max_Warmest | 0.502 |
| *Polioptila caerulea* | 2506 | lqp | 1.2 | 500 | 47022.168 | 19.848 | 0.732 | 1.238 | 0.000 | 0.035 | Moisture_index, Temp_max_Coldest, Elevation | 0.463 |
| *Polioptila melanura* | 869 | lq | 1.0 | 500 | 14629.150 | 1.258 | 0.894 | 1.586 | 0.000 | 0.061 | Slope, Moisture_index, Temp_min_Coldest | 0.289 |
| *Polioptila nigriceps* | 210 | lqp | 0.9 | 500 | 2699.830 | 1.314 | 0.840 | 1.432 | 0.000 | 0.159 | Roughness_index, Precipitation, Elevation | 0.314 |
| *Polioptila plumbea* | 996 | lqp | 1.1 | 500 | 17356.558 | 4.511 | 0.740 | 1.099 | 0.000 | 0.054 | Aridity_index, Temp_max_Warmest, Temp_min_Coldest | 0.555 |
| *Pooecetes gramineus* | 2448 | lqp | 0.8 | 500 | 47686.215 | 0.000 | 0.802 | 1.336 | 0.000 | 0.053 | Moisture_index, Temp_min_Coldest, Elevation | 0.451 |
| *Procnias tricarunculatus* | 64 | qp | 1.0 | 500 | 792.937 | 4.426 | 0.826 | 1.333 | 0.000 | 0.143 | Temp_max_Warmest, Elevation | 0.475 |
| *Psaltriparus minimus* | 1540 | lqp | 0.8 | 500 | 27040.447 | 0.000 | 0.816 | 1.300 | 0.000 | 0.033 | Roughness_index, Moisture_index, Temp_max_Coldest, Elevation | 0.497 |
| *Psarocolius montezuma* | 269 | qp | 1.0 | 500 | 3795.027 | 0.476 | 0.875 | 1.518 | 0.000 | 0.017 | Roughness_index, Moisture_index, Temp_max_Warmest, Elevation | 0.392 |
| *Psarocolius wagleri* | 329 | lqp | 0.9 | 500 | 4706.654 | 0.495 | 0.743 | 1.283 | 0.000 | 0.014 | Moisture_index, Temp_min_Warmest | 0.409 |
| *Pseudoleistes guirahuro* | 1080 | lqp | 0.9 | 500 | 18709.694 | 10.428 | 0.874 | 1.530 | 0.000 | 0.038 | Aridity_index, Moisture_index, Temp_max_Coldest, Temp_max_Warmest, Elevation | 0.348 |
| *Pseudoleistes virescens* | 779 | lqp | 0.9 | 500 | 12023.989 | 2.844 | 0.899 | 1.572 | 0.000 | 0.038 | Moisture_index, Temp_max_Coldest, Elevation | 0.222 |
| *Pteroglossus frantzii* | 82 | qp | 1.1 | 500 | 760.853 | 0.562 | 0.653 | 1.234 | 0.000 | 0.250 | pp_coldest_q, temp_mean_coldest_q, temp_range_diurnal, temp_seasonality | 0.631 |
| *Pteroglossus torquatus* | 366 | lqp | 0.8 | 500 | 5599.686 | 0.000 | 0.761 | 1.247 | 0.000 | 0.024 | Aridity_index, Moisture_index, Temp_min_Warmest | 0.595 |
| *Ptilogonys caudatus* | 47 | qp | 0.8 | 500 | 504.239 | 9.250 | 0.882 | 1.508 | 0.000 | 0.250 | Precipitation, Temp_max_Warmest | 0.190 |
| *Ptilogonys cinereus* | 414 | lqp | 0.9 | 500 | 6631.678 | 0.361 | 0.839 | 1.359 | 0.000 | 0.069 | Slope, Moisture_index, Elevation | 0.326 |
| *Pygiptila stellaris* | 361 | lqp | 0.8 | 500 | 6922.106 | 0.000 | 0.720 | 1.233 | 0.000 | 0.044 | Moisture_index, Temp_max_Coldest, Elevation | 0.525 |
| *Pyrilia haematotis* | 211 | lqp | 0.8 | 500 | 3006.053 | 1.583 | 0.813 | 1.412 | 0.000 | 0.000 | Moisture_index, Temp_max_Coldest | 0.634 |
| *Pyrocephalus rubinus* | 2071 | lqp | 0.8 | 500 | 38731.946 | 0.000 | 0.813 | 1.317 | 0.000 | 0.048 | Moisture_index, Temp_max_Coldest, Temp_max_Warmest, Elevation | 0.459 |
| *Quiscalus lugubris* | 751 | lqp | 0.9 | 500 | 11934.000 | 0.432 | 0.752 | 1.160 | 0.000 | 0.053 | Moisture_index, Elevation | 0.607 |
| *Quiscalus major* | 325 | lq | 0.9 | 500 | 3398.504 | 1.101 | 0.920 | 1.657 | 0.000 | 0.172 | Aridity_index, Moisture_index, Precipitation, Elevation | 0.246 |
| *Quiscalus mexicanus* | 1748 | lqp | 1.1 | 500 | 30456.506 | 9.027 | 0.797 | 1.304 | 0.000 | 0.058 | Slope, Moisture_index, Temp_max_Coldest | 0.412 |
| *Quiscalus nicaraguensis* | 22 | lp | 0.8 | 500 | 299.922 | 0.000 | 0.867 | 1.640 | 0.000 | 0.167 | pp_coldest_q, temp_mean_coldest_q, temp_range_diurnal, temp_seasonality | 0.449 |
| *Quiscalus niger* | 298 | lp | 1.2 | 500 | 2624.091 | 0.709 | 0.464 | 1.067 | 0.000 | 0.021 | Aridity_index, Temp_max_Coldest, Temp_min_Warmest, Precipitation | 0.628 |
| *Quiscalus quiscula* | 2162 | lqp | 0.9 | 500 | 40174.308 | 42.394 | 0.844 | 1.372 | 0.000 | 0.045 | Aridity_index, Temp_max_Warmest, Elevation | 0.404 |
| *Ramphastos sulfuratus* | 301 | lqp | 0.8 | 500 | 4339.773 | 0.000 | 0.785 | 1.267 | 0.000 | 0.045 | Moisture_index, Elevation | 0.374 |
| *Ramphocaenus melanurus* | 1086 | lqp | 0.9 | 500 | 19361.044 | 4.734 | 0.802 | 1.247 | 0.000 | 0.024 | Roughness_index, Aridity_index, Temp_max_Warmest, Temp_min_Coldest | 0.482 |
| *Ramphocelus bresilius* | 377 | lqp | 0.9 | 500 | 4731.104 | 0.179 | 0.750 | 1.079 | 0.122 | 0.028 | Roughness_index, Elevation | 0.512 |
| *Ramphocelus carbo* | 1226 | lq | 0.8 | 500 | 24003.150 | 9.058 | 0.639 | 1.110 | 0.000 | 0.060 | Moisture_index, Temp_max_Coldest, Temp_min_Warmest | 0.666 |
| *Ramphocelus costaricensis* | 13 | lqph | 1.0 | 500 | NA | NA | 0.612 | 1.545 | 0.000 | 0.500 | Slope, Roughness_index, Aridity_index, Moisture_index, Temp_max_Coldest, Temp_min_Warmest, Precipitation, Temp_max_Warmest, Temp_min_Coldest, Elevation | 0.576 |
| *Ramphocelus dimidiatus* | 369 | lq | 0.8 | 500 | 5410.421 | 0.000 | 0.688 | 1.153 | 0.000 | 0.046 | Moisture_index, Elevation | 0.559 |
| *Ramphocelus flammigerus* | 293 | lqp | 1.1 | 500 | 3955.414 | 2.290 | 0.812 | 1.333 | 0.000 | 0.083 | Aridity_index, Temp_min_Warmest | 0.391 |
| *Ramphocelus icteronotus* | 71 | lq | 1.1 | 500 | 992.388 | 2.562 | 0.633 | 1.161 | 0.000 | 0.125 | Aridity_index, Temp_min_Warmest | 0.586 |
| *Ramphocelus melanogaster* | 48 | lq | 1.2 | 500 | 755.642 | 0.939 | 0.884 | 1.517 | 0.000 | 0.083 | pp_coldest_q, temp_mean_coldest_q, temp_range_diurnal, temp_seasonality | 0.379 |
| *Ramphocelus nigrogularis* | 317 | lqp | 0.9 | 500 | 5703.728 | 0.643 | 0.781 | 1.199 | 0.000 | 0.101 | Aridity_index, Temp_max_Coldest, Elevation | 0.317 |
| *Ramphocelus passerinii* | 147 | lq | 1.0 | 500 | 2044.731 | 0.309 | 0.821 | 1.326 | 0.000 | 0.088 | Moisture_index, Temp_max_Coldest, Elevation | 0.467 |
| *Ramphocelus sanguinolentus* | 150 | lq | 0.8 | 500 | 2163.281 | 0.604 | 0.800 | 1.160 | 0.000 | 0.086 | Roughness_index, Aridity_index, Temp_max_Coldest | 0.411 |
| *Regulus calendula* | 2461 | lq | 0.9 | 500 | 48131.444 | 13.051 | 0.762 | 1.185 | 0.000 | 0.054 | Roughness_index, Precipitation, Temp_max_Warmest | 0.548 |
| *Regulus regulus* | 3152 | lqp | 0.8 | 500 | 56128.180 | 0.000 | 0.953 | 1.715 | 0.000 | 0.049 | Temp_max_Coldest, Temp_max_Warmest, Elevation | 0.328 |
| *Regulus satrapa* | 2391 | lqp | 0.8 | 500 | 44132.012 | 0.000 | 0.891 | 1.474 | 0.000 | 0.027 | Roughness_index, Precipitation, Temp_max_Warmest | 0.468 |
| *Rhodinocichla rosea* | 146 | lqp | 1.0 | 500 | 2132.494 | 2.923 | 0.712 | 1.063 | 0.000 | 0.062 | Slope, Aridity_index, Moisture_index | 0.518 |
| *Rhodothraupis celaeno* | 80 | lq | 1.2 | 500 | 1171.572 | 10.313 | 0.823 | 1.371 | 0.000 | 0.105 | Temp_max_Coldest, Elevation | 0.673 |
| *Rhynchocyclus brevirostris* | 188 | lqp | 1.0 | 500 | 2720.884 | 3.344 | 0.734 | 1.208 | 0.000 | 0.048 | Moisture_index, Temp_max_Coldest, Temp_min_Warmest | 0.554 |
| *Rhynchopsitta pachyrhyncha* | 25 | lp | 1.1 | 500 | 343.366 | 0.777 | 0.613 | 1.279 | 0.000 | 0.167 | pp_coldest_q, temp_mean_coldest_q, temp_range_diurnal, temp_seasonality | 0.641 |
| *Rhynchopsitta terrisi* | 22 | lq | 0.8 | 500 | 227.507 | 0.000 | 0.837 | 1.557 | 0.000 | 0.200 | pp_coldest_q, temp_mean_coldest_q, temp_range_diurnal, temp_seasonality | 0.411 |
| *Rhynchortyx cinctus* | 55 | qp | 0.9 | 500 | 770.820 | 1.215 | 0.645 | 1.300 | 0.000 | 0.000 | pp_coldest_q, temp_mean_coldest_q, temp_range_diurnal, temp_seasonality | 0.672 |
| *Rhytipterna holerythra* | 210 | lqp | 0.9 | 500 | 3180.399 | 0.920 | 0.740 | 1.217 | 0.000 | 0.146 | Aridity_index, Temp_max_Warmest | 0.417 |
| *Salpinctes obsoletus* | 2640 | lqp | 0.8 | 500 | 48911.982 | 0.000 | 0.816 | 1.361 | 0.000 | 0.053 | Roughness_index, Aridity_index, Temp_max_Coldest | 0.413 |
| *Saltator atriceps* | 7634 | qp | 0.8 | 500 | 117974.571 | 166.390 | 0.827 | 1.373 | 0.000 | 0.048 | Temp_max_Warmest, Temp_min_Coldest | 0.570 |
| *Saltator coerulescens* | 1167 | lqp | 0.8 | 500 | 21791.632 | 0.000 | 0.683 | 1.067 | 0.000 | 0.073 | Moisture_index, Temp_max_Coldest, Elevation | 0.559 |
| *Saltator grossus* | 613 | lq | 0.8 | 500 | 11321.004 | 0.000 | 0.754 | 1.191 | 0.000 | 0.047 | Roughness_index, Temp_max_Warmest, Temp_min_Coldest | 0.558 |
| *Saltator maximus* | 907 | lq | 0.8 | 500 | 16541.928 | 19.991 | 0.766 | 1.099 | 0.000 | 0.056 | Roughness_index, Moisture_index, Temp_max_Coldest | 0.474 |
| *Sayornis nigricans* | 1639 | lqp | 0.8 | 500 | 29438.951 | 0.000 | 0.831 | 1.388 | 0.000 | 0.052 | Roughness_index, Aridity_index, Temp_max_Coldest | 0.455 |
| *Sayornis phoebe* | 1848 | lqp | 0.8 | 500 | 34180.768 | 0.000 | 0.870 | 1.506 | 0.000 | 0.049 | Moisture_index, Temp_min_Coldest, Elevation | 0.304 |
| *Sayornis saya* | 2302 | lqp | 1.2 | 500 | 43040.760 | 12.067 | 0.875 | 1.503 | 0.000 | 0.052 | Moisture_index, Temp_min_Coldest, Elevation | 0.369 |
| *Schiffornis turdina* | 730 | lqp | 1.2 | 500 | 13873.736 | 2.092 | 0.750 | 1.208 | 0.000 | 0.034 | Roughness_index, Moisture_index, Temp_max_Coldest | 0.510 |
| *Schiffornis virescens* | 559 | lq | 0.8 | 500 | 8490.446 | 8.036 | 0.943 | 1.708 | 0.000 | 0.031 | Roughness_index, Moisture_index, Temp_max_Coldest, Temp_max_Warmest | 0.230 |
| *Sclerurus guatemalensis* | 127 | lqp | 0.8 | 500 | 1882.104 | 0.000 | 0.797 | 1.313 | 0.000 | 0.034 | Roughness_index, Moisture_index, Temp_max_Coldest | 0.498 |
| *Sclerurus mexicanus* | 328 | lq | 0.8 | 500 | 6036.736 | 0.000 | 0.843 | 1.287 | 0.000 | 0.062 | Roughness_index, Moisture_index, Temp_max_Warmest | 0.329 |
| *Selasphorus platycercus* | 1096 | lqp | 0.9 | 500 | 19542.033 | 7.438 | 0.854 | 1.373 | 0.000 | 0.048 | Moisture_index, Temp_max_Coldest, Elevation | 0.388 |
| *Selasphorus rufus* | 1390 | lqp | 0.8 | 500 | 23350.217 | 0.000 | 0.892 | 1.451 | 0.000 | 0.039 | Roughness_index, Aridity_index, Temp_max_Coldest, Elevation | 0.385 |
| *Selasphorus sasin* | 209 | lp | 0.8 | 500 | 2115.349 | 0.983 | 0.981 | 1.901 | 0.000 | 0.000 | Roughness_index, Moisture_index, Temp_max_Coldest | 0.131 |
| *Selenidera spectabilis* | 65 | lqp | 0.8 | 500 | 909.534 | 0.000 | 0.820 | 1.389 | 0.000 | 0.067 | pp_coldest_q, temp_mean_coldest_q, temp_range_diurnal, temp_seasonality | 0.558 |
| *Sialia currucoides* | 2038 | lqp | 0.8 | 500 | 38099.301 | 0.000 | 0.893 | 1.549 | 0.000 | 0.033 | Temp_max_Coldest, Temp_min_Warmest, Precipitation, Elevation | 0.279 |
| *Sialia mexicana* | 1585 | lqp | 0.8 | 500 | 28435.157 | 0.000 | 0.833 | 1.297 | 0.000 | 0.065 | Roughness_index, Moisture_index, Temp_max_Coldest, Elevation | 0.419 |
| *Sialia sialis* | 2140 | lqp | 1.2 | 500 | 39810.761 | 6.650 | 0.824 | 1.377 | 0.000 | 0.069 | Moisture_index, Temp_max_Coldest, Elevation | 0.331 |
| *Sicalis luteola* | 2063 | lqp | 1.1 | 500 | 36247.492 | 12.252 | 0.883 | 1.298 | 0.000 | 0.047 | Aridity_index, Temp_max_Coldest, Elevation | 0.429 |
| *Sitta canadensis* | 2322 | lq | 0.9 | 500 | 43614.019 | 105.606 | 0.868 | 1.330 | 0.000 | 0.040 | Roughness_index, Precipitation, Temp_max_Warmest | 0.544 |
| *Sitta carolinensis* | 2168 | lqp | 1.0 | 500 | 41518.505 | 7.620 | 0.820 | 1.252 | 0.000 | 0.051 | Moisture_index, Temp_max_Coldest, Elevation | 0.452 |
| *Sitta pygmaea* | 992 | lqp | 0.8 | 500 | 17446.926 | 0.000 | 0.878 | 1.406 | 0.000 | 0.033 | Roughness_index, Temp_max_Coldest, Precipitation, Elevation | 0.430 |
| *Sittasomus griseicapillus* | 2091 | lqp | 0.8 | 500 | 40017.196 | 0.000 | 0.750 | 1.084 | 0.000 | 0.045 | Aridity_index, Temp_min_Coldest, Elevation | 0.591 |
| *Sphyrapicus nuchalis* | 1506 | lqp | 0.8 | 500 | 27769.723 | 0.000 | 0.836 | 1.299 | 0.000 | 0.061 | Roughness_index, Temp_max_Coldest, Precipitation | 0.410 |
| *Sphyrapicus ruber* | 1123 | lqp | 1.0 | 500 | 17849.590 | 11.333 | 0.902 | 1.359 | 0.000 | 0.056 | Slope, Temp_max_Coldest, Precipitation, Elevation | 0.246 |
| *Sphyrapicus thyroideus* | 1244 | lqp | 1.2 | 500 | 21939.084 | 26.229 | 0.836 | 1.331 | 0.000 | 0.055 | Roughness_index, Temp_max_Coldest, Precipitation, Elevation | 0.389 |
| *Sphyrapicus varius* | 1614 | lqp | 0.8 | 500 | 28742.835 | 0.000 | 0.912 | 1.495 | 0.000 | 0.059 | Roughness_index, Moisture_index, Temp_min_Warmest, Temp_min_Coldest | 0.295 |
| *Spiza americana* | 1203 | lqp | 0.8 | 500 | 22543.031 | 0.093 | 0.781 | 1.299 | 0.000 | 0.047 | Moisture_index, Temp_min_Warmest | 0.436 |
| *Spizella atrogularis* | 838 | lqp | 0.8 | 500 | 14557.668 | 0.000 | 0.886 | 1.515 | 0.000 | 0.063 | Roughness_index, Temp_max_Coldest, Precipitation, Elevation | 0.324 |
| *Spizella breweri* | 1900 | lqp | 1.2 | 500 | 34783.484 | 27.701 | 0.871 | 1.502 | 0.000 | 0.034 | Roughness_index, Aridity_index, Temp_max_Coldest, Elevation | 0.396 |
| *Spizella pallida* | 1476 | lqp | 0.8 | 500 | 27070.079 | 0.000 | 0.899 | 1.527 | 0.000 | 0.058 | Moisture_index, Temp_max_Coldest, Temp_min_Warmest, Elevation | 0.309 |
| *Spizella passerina* | 3002 | lqp | 1.0 | 500 | 59533.452 | 88.756 | 0.813 | 1.292 | 0.000 | 0.057 | Precipitation, Temp_max_Warmest, Elevation | 0.547 |
| *Spizella pusilla* | 1617 | lqp | 0.9 | 500 | 29604.946 | 15.447 | 0.843 | 1.420 | 0.000 | 0.043 | Temp_min_Warmest, Precipitation | 0.462 |
| *Spizella wortheni* | 19 | qp | 0.9 | 500 | 304.045 | 0.029 | 0.487 | 1.255 | 0.000 | 0.000 | pp_coldest_q, temp_mean_coldest_q, temp_range_diurnal, temp_seasonality | 0.635 |
| *Sporophila corvina* | 320 | lp | 0.8 | 500 | 4326.229 | 2.471 | 0.723 | 1.163 | 0.000 | 0.091 | Moisture_index, Temp_max_Coldest, Temp_max_Warmest | 0.506 |
| *Sporophila minuta* | 924 | lqp | 1.1 | 500 | 15341.633 | 1.567 | 0.702 | 1.126 | 0.000 | 0.040 | Aridity_index, Temp_min_Warmest | 0.610 |
| *Sporophila schistacea* | 184 | qp | 1.2 | 500 | 3232.752 | 2.495 | 0.783 | 1.187 | 0.000 | 0.070 | Slope, Aridity_index, Temp_min_Warmest, Temp_max_Warmest | 0.548 |
| *Sporophila torqueola* | 599 | lq | 0.9 | 500 | 8717.063 | 0.130 | 0.744 | 1.314 | 0.000 | 0.064 | Moisture_index, Elevation | 0.351 |
| *Streptoprocne rutila* | 750 | lqp | 0.9 | 500 | 12366.181 | 0.294 | 0.802 | 1.173 | 0.000 | 0.090 | Roughness_index, Moisture_index | 0.262 |
| *Streptoprocne semicollaris* | 95 | lq | 0.9 | 500 | 1385.110 | 1.075 | 0.774 | 1.265 | 0.000 | 0.143 | Slope, Aridity_index, Temp_min_Warmest, Precipitation | 0.490 |
| *Streptoprocne zonaris* | 1316 | lqp | 0.8 | 500 | 23865.211 | 0.000 | 0.836 | 1.231 | 0.000 | 0.029 | Roughness_index, Moisture_index, Temp_max_Coldest | 0.363 |
| *Sturnella magna* | 2258 | lqp | 0.9 | 500 | 42971.883 | 0.844 | 0.788 | 1.225 | 0.000 | 0.066 | Moisture_index, Temp_max_Coldest, Elevation | 0.449 |
| *Sturnella neglecta* | 2877 | lqp | 0.9 | 500 | 55001.917 | 6.755 | 0.784 | 1.261 | 0.000 | 0.064 | Moisture_index, Temp_min_Coldest, Elevation | 0.422 |
| *Sturnus unicolor* | 1720 | lqp | 0.8 | 500 | 26432.336 | 0.000 | 0.833 | 1.520 | 0.000 | 0.038 | Moisture_index, Elevation | 0.294 |
| *Sturnus vulgaris* | 3248 | lq | 0.8 | 500 | 56684.577 | 41.133 | 0.943 | 1.658 | 0.000 | 0.042 | Temp_max_Coldest, Temp_max_Warmest, Elevation | 0.335 |
| *Synallaxis brachyura* | 266 | lqp | 0.9 | 500 | 3935.061 | 0.833 | 0.775 | 1.298 | 0.000 | 0.032 | Moisture_index, Temp_max_Warmest, Temp_min_Coldest | 0.375 |
| *Synallaxis erythrothorax* | 110 | qp | 1.2 | 500 | 1583.621 | 0.229 | 0.740 | 1.219 | 0.000 | 0.115 | Aridity_index, Temp_max_Coldest, Precipitation, Elevation | 0.533 |
| *Tangara cabanisi* | 12 | lq | 0.8 | 500 | 167.502 | 0.925 | 0.991 | 1.978 | 0.000 | 0.000 | pp_coldest_q, temp_mean_coldest_q, temp_range_diurnal, temp_seasonality | 0.329 |
| *Tangara larvata* | 269 | lqp | 0.9 | 500 | 3757.012 | 1.633 | 0.821 | 1.435 | 0.000 | 0.017 | Roughness_index, Moisture_index, Temp_max_Coldest | 0.422 |
| *Tangara lavinia* | 100 | lq | 0.8 | 500 | 1411.332 | 0.000 | 0.790 | 1.342 | 0.000 | 0.087 | Moisture_index, Temp_max_Warmest | 0.534 |
| *Tapera naevia* | 1644 | lqp | 1.1 | 500 | 31194.073 | 4.025 | 0.696 | 1.106 | 0.000 | 0.028 | Roughness_index, Aridity_index, Temp_min_Coldest | 0.583 |
| *Taraba major* | 1536 | lqp | 0.8 | 500 | 29929.015 | 0.000 | 0.699 | 1.092 | 0.000 | 0.043 | Moisture_index, Temp_max_Coldest, Elevation | 0.609 |
| *Terenotriccus erythrurus* | 687 | lqp | 1.1 | 500 | 12887.634 | 4.124 | 0.777 | 1.251 | 0.000 | 0.048 | Roughness_index, Moisture_index, Temp_max_Coldest, Temp_max_Warmest | 0.448 |
| *Thalurania colombica* | 409 | lqp | 0.8 | 500 | 6095.464 | 0.000 | 0.813 | 1.317 | 0.000 | 0.085 | Aridity_index, Precipitation, Temp_max_Warmest, Temp_min_Coldest | 0.310 |
| *Thalurania ridgwayi* | 27 | lqph | 1.0 | 500 | NA | NA | 0.887 | 1.423 | 0.000 | 0.556 | Slope, Roughness_index, Aridity_index, Moisture_index, Temp_max_Coldest, Temp_min_Warmest, Precipitation, Temp_max_Warmest, Temp_min_Coldest, Elevation | 0.449 |
| *Thamnistes anabatinus* | 137 | lq | 0.8 | 500 | 1991.571 | 0.000 | 0.871 | 1.366 | 0.000 | 0.031 | Slope, Moisture_index, Temp_max_Coldest | 0.361 |
| *Thamnophilus atrinucha* | 335 | lq | 0.9 | 500 | 4809.557 | 0.856 | 0.679 | 1.115 | 0.000 | 0.056 | Aridity_index, Temp_max_Coldest | 0.574 |
| *Thamnophilus doliatus* | 1270 | lqp | 0.8 | 500 | 24210.432 | 0.000 | 0.675 | 1.067 | 0.000 | 0.056 | Roughness_index, Moisture_index, Temp_max_Coldest | 0.579 |
| *Thraupis abbas* | 262 | lqp | 0.8 | 500 | 3979.010 | 0.845 | 0.829 | 1.409 | 0.000 | 0.017 | Moisture_index, Temp_min_Warmest | 0.349 |
| *Thraupis episcopus* | 1150 | lqp | 0.9 | 500 | 20671.941 | 3.489 | 0.717 | 1.052 | 0.000 | 0.053 | Roughness_index, Moisture_index, Temp_max_Warmest | 0.530 |
| *Threnetes ruckeri* | 219 | lqp | 0.9 | 500 | 3269.608 | 0.788 | 0.830 | 1.387 | 0.000 | 0.140 | Aridity_index, Temp_max_Warmest, Temp_min_Coldest | 0.386 |
| *Thryomanes bewickii* | 1854 | lqp | 1.1 | 500 | 33124.676 | 25.746 | 0.838 | 1.383 | 0.000 | 0.061 | Aridity_index, Temp_max_Coldest, Elevation | 0.382 |
| *Thryothorus felix* | 308 | qp | 0.9 | 500 | 4011.155 | 5.446 | 0.712 | 1.238 | 0.000 | 0.094 | Roughness_index, Aridity_index, Moisture_index, Temp_max_Warmest | 0.365 |
| *Thryothorus ludovicianus* | 1551 | lqp | 0.9 | 500 | 27089.731 | 2.870 | 0.765 | 1.327 | 0.000 | 0.041 | Moisture_index, Temp_max_Coldest, Elevation | 0.468 |
| *Thryothorus maculipectus* | 556 | lqp | 1.0 | 500 | 8312.672 | 2.989 | 0.734 | 1.228 | 0.000 | 0.055 | Roughness_index, Aridity_index, Temp_min_Coldest | 0.485 |
| *Thryothorus modestus* | 456 | lqp | 0.9 | 500 | 6307.547 | 0.064 | 0.756 | 1.257 | 0.000 | 0.049 | Temp_min_Warmest, Temp_min_Coldest | 0.384 |
| *Thryothorus nigricapillus* | 256 | lq | 0.8 | 500 | 3522.607 | 2.860 | 0.680 | 1.074 | 0.119 | 0.107 | Aridity_index, Precipitation, Temp_max_Warmest | 0.510 |
| *Thryothorus pleurostictus* | 178 | lqp | 0.8 | 500 | 2407.626 | 0.000 | 0.698 | 1.179 | 0.000 | 0.053 | Moisture_index, Temp_min_Warmest | 0.467 |
| *Thryothorus rufalbus* | 388 | lq | 0.9 | 500 | 6165.806 | 1.574 | 0.792 | 1.291 | 0.000 | 0.056 | Aridity_index, Temp_max_Warmest | 0.441 |
| *Thryothorus sinaloa* | 273 | lq | 0.9 | 500 | 3541.609 | 3.207 | 0.870 | 1.480 | 0.000 | 0.052 | Roughness_index, Moisture_index, Temp_min_Warmest, Temp_min_Coldest | 0.283 |
| *Thryothorus thoracicus* | 56 | lq | 1.0 | 500 | 652.762 | 0.558 | 0.842 | 1.552 | 0.000 | 0.167 | Slope, Moisture_index, Temp_max_Coldest | 0.279 |
| *Tiaris olivaceus* | 624 | lqp | 0.8 | 500 | 9410.649 | 7.032 | 0.799 | 1.257 | 0.000 | 0.074 | Aridity_index, Temp_max_Warmest, Temp_min_Coldest | 0.447 |
| *Tilmatura dupontii* | 135 | lqp | 1.2 | 500 | 1882.989 | 0.936 | 0.843 | 1.222 | 0.000 | 0.065 | Aridity_index, Moisture_index, Elevation | 0.372 |
| *Tinamus major* | 928 | lqp | 0.9 | 500 | 17147.204 | 3.223 | 0.762 | 1.196 | 0.000 | 0.062 | Roughness_index, Moisture_index, Temp_max_Coldest, Temp_max_Warmest | 0.529 |
| *Tityra cayana* | 1302 | lqp | 0.8 | 500 | 24912.234 | 0.000 | 0.717 | 1.166 | 0.000 | 0.047 | Moisture_index, Temp_max_Coldest, Elevation | 0.528 |
| *Tityra inquisitor* | 1379 | lqp | 1.2 | 500 | 26091.688 | 17.753 | 0.703 | 1.152 | 0.000 | 0.046 | Roughness_index, Moisture_index, Temp_min_Warmest | 0.439 |
| *Tityra semifasciata* | 824 | lqp | 0.8 | 500 | 14701.506 | 0.000 | 0.780 | 1.127 | 0.000 | 0.057 | Roughness_index, Moisture_index, Temp_max_Coldest, Temp_max_Warmest | 0.482 |
| *Todirostrum cinereum* | 1299 | lqp | 0.8 | 500 | 23678.623 | 0.000 | 0.698 | 1.054 | 0.000 | 0.054 | Moisture_index, Temp_max_Coldest | 0.584 |
| *Tolmomyias sulphurescens* | 1248 | lqp | 0.8 | 500 | 22922.310 | 0.000 | 0.788 | 1.159 | 0.000 | 0.034 | Roughness_index, Moisture_index, Temp_max_Coldest | 0.587 |
| *Toxostoma bendirei* | 380 | lqp | 0.8 | 500 | 6484.745 | 0.000 | 0.828 | 1.277 | 0.000 | 0.118 | Slope, Temp_max_Warmest | 0.321 |
| *Toxostoma cinereum* | 134 | lq | 0.8 | 500 | 1381.481 | 0.000 | 0.906 | 1.620 | 0.000 | 0.080 | Roughness_index, Aridity_index, Temp_max_Coldest, Temp_max_Warmest | 0.140 |
| *Toxostoma crissale* | 784 | lqp | 1.2 | 500 | 13426.927 | 39.581 | 0.898 | 1.560 | 0.000 | 0.071 | Slope, Moisture_index, Temp_max_Coldest, Elevation | 0.349 |
| *Toxostoma curvirostre* | 1242 | lqp | 0.8 | 500 | 21881.488 | 0.000 | 0.853 | 1.510 | 0.000 | 0.033 | Moisture_index, Temp_max_Coldest, Elevation | 0.242 |
| *Toxostoma longirostre* | 262 | lqp | 0.8 | 500 | 3934.000 | 0.819 | 0.938 | 1.648 | 0.000 | 0.109 | Roughness_index, Aridity_index, Temp_max_Coldest, Temp_max_Warmest | 0.274 |
| *Toxostoma ocellatum* | 74 | qp | 1.2 | 500 | 1098.197 | 0.315 | 0.869 | 1.516 | 0.000 | 0.000 | Roughness_index, Aridity_index, Temp_min_Warmest | 0.462 |
| *Toxostoma rufum* | 1819 | lqp | 0.9 | 500 | 33414.163 | 3.207 | 0.813 | 1.302 | 0.000 | 0.051 | Roughness_index, Moisture_index, Temp_min_Warmest | 0.421 |
| *Troglodytes aedon* | 3075 | lqp | 1.0 | 500 | 61975.824 | 5.590 | 0.739 | 1.118 | 0.000 | 0.048 | Moisture_index, Temp_max_Coldest, Elevation | 0.575 |
| *Troglodytes rufociliatus* | 118 | lq | 1.0 | 500 | 1528.673 | 0.173 | 0.895 | 1.304 | 0.000 | 0.133 | Aridity_index, Temp_max_Warmest, Temp_min_Coldest | 0.227 |
| *Trogon citreolus* | 197 | lqp | 0.8 | 500 | 2288.087 | 0.268 | 0.835 | 1.270 | 0.000 | 0.027 | Aridity_index, Temp_min_Warmest | 0.516 |
| *Trogon collaris* | 853 | lqp | 0.9 | 500 | 15893.650 | 9.469 | 0.779 | 1.146 | 0.000 | 0.052 | Roughness_index, Moisture_index, Temp_max_Coldest | 0.445 |
| *Trogon elegans* | 125 | lq | 0.8 | 500 | 1585.884 | 0.000 | 0.808 | 1.322 | 0.000 | 0.037 | Moisture_index, Temp_min_Warmest | 0.443 |
| *Trogon massena* | 241 | qp | 0.9 | 500 | 3252.836 | 0.433 | 0.770 | 1.336 | 0.000 | 0.039 | Moisture_index, Temp_max_Coldest, Temp_min_Warmest | 0.329 |
| *Trogon melanocephalus* | 197 | qp | 1.0 | 500 | 2688.977 | 0.010 | 0.674 | 1.204 | 0.000 | 0.024 | Aridity_index, Moisture_index | 0.720 |
| *Trogon mexicanus* | 296 | lqp | 0.8 | 500 | 4610.043 | 0.000 | 0.843 | 1.236 | 0.000 | 0.041 | Aridity_index, Moisture_index, Temp_min_Warmest | 0.399 |
| *Trogon personatus* | 378 | lqp | 1.2 | 500 | 6191.445 | 2.456 | 0.952 | 1.754 | 0.000 | 0.021 | Roughness_index, Aridity_index, Temp_min_Coldest | 0.134 |
| *Trogon rufus* | 769 | lq | 1.0 | 500 | 14015.246 | 5.172 | 0.812 | 1.272 | 0.000 | 0.065 | Roughness_index, Moisture_index, Temp_max_Coldest | 0.425 |
| *Trogon violaceus* | 578 | lq | 0.8 | 500 | 10610.353 | 0.000 | 0.723 | 1.109 | 0.059 | 0.066 | Roughness_index, Moisture_index, Temp_max_Coldest | 0.495 |
| *Turdus assimilis* | 432 | lqp | 1.1 | 500 | 6640.419 | 0.000 | 0.825 | 1.313 | 0.000 | 0.039 | Roughness_index, Moisture_index, Temp_min_Warmest | 0.378 |
| *Turdus grayi* | 490 | lqp | 0.8 | 500 | 7607.869 | 0.000 | 0.637 | 1.113 | 0.000 | 0.073 | Temp_max_Coldest, Temp_min_Warmest | 0.524 |
| *Turdus infuscatus* | 111 | lq | 0.9 | 500 | 1577.660 | 0.233 | 0.882 | 1.397 | 0.000 | 0.071 | Aridity_index, Moisture_index, Temp_max_Coldest, Temp_min_Warmest | 0.342 |
| *Turdus migratorius* | 2925 | lqp | 0.8 | 500 | 56430.075 | 0.000 | 0.850 | 1.407 | 0.000 | 0.046 | Moisture_index, Temp_max_Coldest, Elevation | 0.393 |
| *Turdus plebejus* | 90 | lq | 0.8 | 500 | 1262.616 | 0.000 | 0.911 | 1.504 | 0.000 | 0.091 | Temp_max_Coldest, Temp_min_Warmest, Precipitation | 0.191 |
| *Turdus rufitorques* | 110 | lq | 0.9 | 500 | 1461.555 | 1.558 | 0.891 | 1.282 | 0.000 | 0.143 | Temp_max_Coldest, Temp_min_Warmest, Precipitation | 0.467 |
| *Turdus rufopalliatus* | 353 | lq | 0.8 | 500 | 5113.945 | 1.295 | 0.791 | 1.298 | 0.000 | 0.064 | Slope, Temp_max_Coldest, Temp_min_Warmest | 0.376 |
| *Tympanuchus cupido* | 447 | lqp | 0.8 | 500 | 7913.729 | 0.000 | 0.920 | 1.496 | 0.000 | 0.063 | Roughness_index, Temp_max_Coldest, Precipitation | 0.330 |
| *Tympanuchus pallidicinctus* | 34 | lq | 0.8 | 500 | 553.700 | 0.000 | 0.848 | 1.537 | 0.000 | 0.125 | pp_coldest_q, temp_mean_coldest_q, temp_range_diurnal, temp_seasonality | 0.584 |
| *Tyrannus couchii* | 340 | lqp | 1.2 | 500 | 5312.491 | 7.021 | 0.885 | 1.407 | 0.000 | 0.088 | Temp_max_Coldest, Precipitation, Temp_max_Warmest | 0.467 |
| *Tyrannus crassirostris* | 420 | lqp | 1.2 | 500 | 5806.478 | 6.267 | 0.813 | 1.332 | 0.045 | 0.056 | Roughness_index, Moisture_index, Elevation | 0.439 |
| *Tyrannus forficatus* | 926 | lq | 1.0 | 500 | 15933.181 | 16.099 | 0.851 | 1.396 | 0.000 | 0.048 | Slope, Moisture_index, Temp_min_Warmest | 0.285 |
| *Tyrannus melancholicus* | 2093 | lqp | 0.9 | 500 | 38095.669 | 3.547 | 0.672 | 1.081 | 0.000 | 0.083 | Moisture_index, Temp_max_Coldest, Elevation | 0.570 |
| *Tyrannus savana* | 1979 | lqp | 0.8 | 500 | 37425.775 | 0.000 | 0.771 | 1.102 | 0.000 | 0.047 | Moisture_index, Temp_max_Coldest, Elevation | 0.524 |
| *Tyrannus tyrannus* | 2311 | lqp | 0.9 | 500 | 44298.171 | 42.754 | 0.822 | 1.358 | 0.000 | 0.068 | Precipitation, Temp_max_Warmest, Elevation | 0.463 |
| *Tyrannus verticalis* | 2570 | lqp | 0.8 | 500 | 48602.759 | 0.000 | 0.805 | 1.321 | 0.000 | 0.055 | Moisture_index, Temp_max_Coldest, Elevation | 0.402 |
| *Tyrannus vociferans* | 1190 | lqp | 0.9 | 500 | 21082.633 | 12.109 | 0.840 | 1.445 | 0.000 | 0.031 | Roughness_index, Moisture_index, Temp_max_Coldest, Elevation | 0.470 |
| *Uropsila leucogastra* | 168 | lqp | 0.9 | 500 | 2176.828 | 2.179 | 0.740 | 1.305 | 0.000 | 0.000 | Aridity_index, Temp_max_Coldest, Temp_min_Warmest | 0.539 |
| *Vireo atricapilla* | 172 | lqp | 1.0 | 500 | 2689.630 | 1.541 | 0.956 | 1.755 | 0.000 | 0.070 | Roughness_index, Moisture_index, Temp_min_Coldest | 0.150 |
| *Vireo bellii* | 1254 | lq | 0.8 | 500 | 23178.214 | 0.000 | 0.841 | 1.408 | 0.000 | 0.055 | Roughness_index, Temp_min_Warmest, Precipitation | 0.370 |
| *Vireo brevipennis* | 74 | lq | 0.9 | 500 | 1114.055 | 0.313 | 0.725 | 1.292 | 0.000 | 0.167 | Aridity_index, Temp_min_Coldest | 0.465 |
| *Vireo cassinii* | 1507 | lqp | 0.8 | 500 | 25727.094 | 0.000 | 0.844 | 1.273 | 0.000 | 0.055 | Roughness_index, Temp_max_Warmest, Elevation | 0.473 |
| *Vireo flavoviridis* | 453 | lqp | 0.9 | 500 | 6621.750 | 1.311 | 0.713 | 1.196 | 0.000 | 0.010 | Aridity_index, Moisture_index, Elevation | 0.520 |
| *Vireo gilvus* | 2679 | lqp | 0.8 | 500 | 52522.562 | 0.000 | 0.765 | 1.292 | 0.000 | 0.032 | Aridity_index, Temp_max_Coldest, Elevation | 0.433 |
| *Vireo griseus* | 1486 | qp | 0.8 | 500 | 26073.003 | 11.482 | 0.781 | 1.351 | 0.000 | 0.053 | Aridity_index, Temp_min_Coldest, Elevation | 0.377 |
| *Vireo huttoni* | 1241 | lqp | 1.1 | 500 | 19652.867 | 9.229 | 0.907 | 1.528 | 0.000 | 0.038 | Roughness_index, Moisture_index, Temp_max_Coldest, Elevation | 0.298 |
| *Vireo hypochryseus* | 234 | lqp | 1.2 | 500 | 3025.546 | 9.466 | 0.828 | 1.399 | 0.000 | 0.060 | Roughness_index, Aridity_index, Moisture_index, Temp_max_Coldest, Temp_max_Warmest | 0.417 |
| *Vireo leucophrys* | 574 | lqp | 0.9 | 500 | 9558.705 | 0.615 | 0.893 | 1.351 | 0.000 | 0.056 | Moisture_index, Temp_min_Warmest | 0.336 |
| *Vireo nelsoni* | 66 | lq | 1.0 | 500 | 1001.493 | 3.796 | 0.820 | 1.350 | 0.000 | 0.062 | Slope, Aridity_index, Moisture_index, Elevation | 0.588 |
| *Vireo olivaceus* | 2265 | lqp | 0.8 | 500 | 43132.635 | 0.000 | 0.883 | 1.363 | 0.000 | 0.061 | Moisture_index, Temp_max_Coldest, Elevation | 0.467 |
| *Vireo pallens* | 135 | qp | 0.8 | 500 | 1235.044 | 0.000 | 0.806 | 1.399 | 0.000 | 0.100 | Temp_max_Warmest, Temp_min_Coldest | 0.369 |
| *Vireo philadelphicus* | 1871 | lqp | 0.8 | 500 | 33079.556 | 0.000 | 0.899 | 1.543 | 0.000 | 0.062 | Roughness_index, Moisture_index, Temp_max_Coldest, Temp_min_Warmest | 0.293 |
| *Vireo plumbeus* | 1121 | lqp | 1.0 | 500 | 20082.222 | 19.010 | 0.839 | 1.356 | 0.000 | 0.040 | Roughness_index, Moisture_index, Temp_max_Coldest, Elevation | 0.434 |
| *Vireo solitarius* | 1593 | lqp | 0.8 | 500 | 27801.953 | 0.000 | 0.911 | 1.521 | 0.000 | 0.024 | Roughness_index, Moisture_index, Temp_min_Warmest, Elevation | 0.432 |
| *Vireo vicinior* | 522 | lqp | 1.1 | 500 | 9268.341 | 17.297 | 0.865 | 1.442 | 0.000 | 0.015 | Roughness_index, Temp_max_Coldest, Precipitation | 0.530 |
| *Vireolanius melitophrys* | 115 | lq | 1.1 | 500 | 1667.884 | 0.415 | 0.857 | 1.285 | 0.000 | 0.107 | Aridity_index, Moisture_index, Elevation | 0.422 |
| *Vireolanius pulchellus* | 166 | lq | 0.9 | 500 | 2397.888 | 0.812 | 0.797 | 1.225 | 0.000 | 0.051 | Roughness_index, Moisture_index, Temp_max_Coldest | 0.544 |
| *Volatinia jacarina* | 1923 | lqp | 1.2 | 500 | 35393.435 | 37.773 | 0.727 | 1.133 | 0.000 | 0.059 | Roughness_index, Aridity_index, Temp_max_Coldest | 0.554 |
| *Xanthocephalus xanthocephalus* | 1938 | lqp | 0.8 | 500 | 37088.010 | 0.000 | 0.851 | 1.452 | 0.000 | 0.064 | Moisture_index, Temp_max_Coldest, Elevation | 0.343 |
| *Xanthopsar flavus* | 75 | lqp | 1.1 | 500 | 1173.030 | 3.027 | 0.921 | 1.637 | 0.000 | 0.056 | Moisture_index, Temp_max_Coldest, Elevation | 0.263 |
| *Xenops minutus* | 239 | lqp | 0.8 | 500 | 3192.062 | 0.000 | 0.927 | 1.616 | 0.000 | 0.039 | Slope, Moisture_index, Temp_max_Coldest, Elevation | 0.163 |
| *Xenospiza baileyi* | 13 | qp | 0.8 | 500 | 184.375 | 0.000 | 0.975 | 1.924 | 0.000 | 0.000 | pp_coldest_q, temp_mean_coldest_q, temp_range_diurnal, temp_seasonality | 0.645 |
| *Xenotriccus callizonus* | 25 | qp | 0.8 | 500 | 381.215 | 4.355 | 0.903 | 1.692 | 0.000 | 0.167 | Roughness_index, Aridity_index, Temp_max_Warmest, Temp_min_Coldest | 0.327 |
| *Xenotriccus mexicanus* | 45 | lp | 1.0 | 500 | 609.164 | 0.519 | 0.893 | 1.629 | 0.000 | 0.091 | Aridity_index, Moisture_index, Temp_max_Coldest, Elevation | 0.441 |
| *Xiphocolaptes promeropirhynchus* | 642 | qp | 0.8 | 500 | 11911.453 | 0.000 | 0.824 | 1.111 | 0.000 | 0.081 | Roughness_index, Aridity_index, Temp_max_Warmest, Temp_min_Coldest | 0.329 |
| *Xiphorhynchus erythropygius* | 265 | lqp | 0.8 | 500 | 4063.405 | 0.000 | 0.796 | 1.214 | 0.000 | 0.063 | Moisture_index, Temp_max_Warmest | 0.469 |
| *Xiphorhynchus flavigaster* | 372 | lqp | 0.9 | 500 | 5531.637 | 0.918 | 0.711 | 1.272 | 0.000 | 0.084 | Moisture_index, Temp_min_Warmest | 0.494 |
| *Xiphorhynchus guttatus* | 1156 | lqp | 0.8 | 500 | 22491.535 | 1.331 | 0.678 | 1.165 | 0.000 | 0.046 | Roughness_index, Temp_max_Coldest, Precipitation | 0.476 |
| *Xiphorhynchus lachrymosus* | 142 | lqp | 0.8 | 500 | 1957.838 | 1.724 | 0.773 | 1.354 | 0.000 | 0.031 | Moisture_index, Temp_max_Coldest | 0.540 |
| *Xiphorhynchus susurrans* | 431 | lqp | 0.8 | 500 | 6358.296 | 0.000 | 0.657 | 1.133 | 0.000 | 0.032 | Roughness_index, Precipitation | 0.636 |
| *Xiphorhynchus triangularis* | 247 | lqp | 0.9 | 500 | 3891.619 | 0.221 | 0.929 | 1.599 | 0.000 | 0.049 | Moisture_index, Temp_min_Warmest | 0.267 |
| *Zimmerius vilissimus* | 247 | lqp | 1.0 | 500 | 3546.392 | 3.847 | 0.829 | 1.388 | 0.000 | 0.055 | Aridity_index, Precipitation, Temp_max_Warmest, Temp_min_Coldest | 0.363 |
| *Zonotrichia albicollis* | 1789 | lqp | 0.8 | 500 | 31886.293 | 0.000 | 0.888 | 1.408 | 0.000 | 0.031 | Precipitation, Temp_max_Warmest, Elevation | 0.532 |
| *Zonotrichia atricapilla* | 1026 | lqp | 0.8 | 500 | 16364.546 | 0.000 | 0.856 | 1.276 | 0.000 | 0.080 | Temp_max_Coldest, Precipitation, Temp_max_Warmest, Elevation | 0.284 |
| *Zonotrichia capensis* | 1816 | lqp | 0.8 | 500 | 33109.663 | 0.000 | 0.846 | 1.396 | 0.000 | 0.056 | Moisture_index, Temp_max_Coldest, Elevation | 0.329 |
| *Zonotrichia leucophrys* | 1709 | lqp | 0.9 | 500 | 31320.881 | 2.409 | 0.858 | 1.255 | 0.000 | 0.060 | Aridity_index, Temp_max_Coldest, Elevation | 0.430 |
| *Zonotrichia querula* | 187 | lqp | 0.8 | 500 | 3135.823 | 0.000 | 0.884 | 1.501 | 0.000 | 0.140 | Roughness_index, Moisture_index, Temp_min_Warmest, Elevation | 0.378 |
| *Zoothera pinicola* | 105 | qp | 1.2 | 500 | 1570.993 | 1.945 | 0.824 | 1.203 | 0.000 | 0.115 | Roughness_index, Aridity_index, Moisture_index, Elevation | 0.240 |
