# Supplementary figures and images for: Niche conservatism and convergence in birds of three cenocrons in the Mexican Transition Zone
Source: PeerJ. 2024 Jan 2;12:e16664. doi: 10.7717/peerj.16664 (PMC10768671; doi:10.7717/peerj.16664)

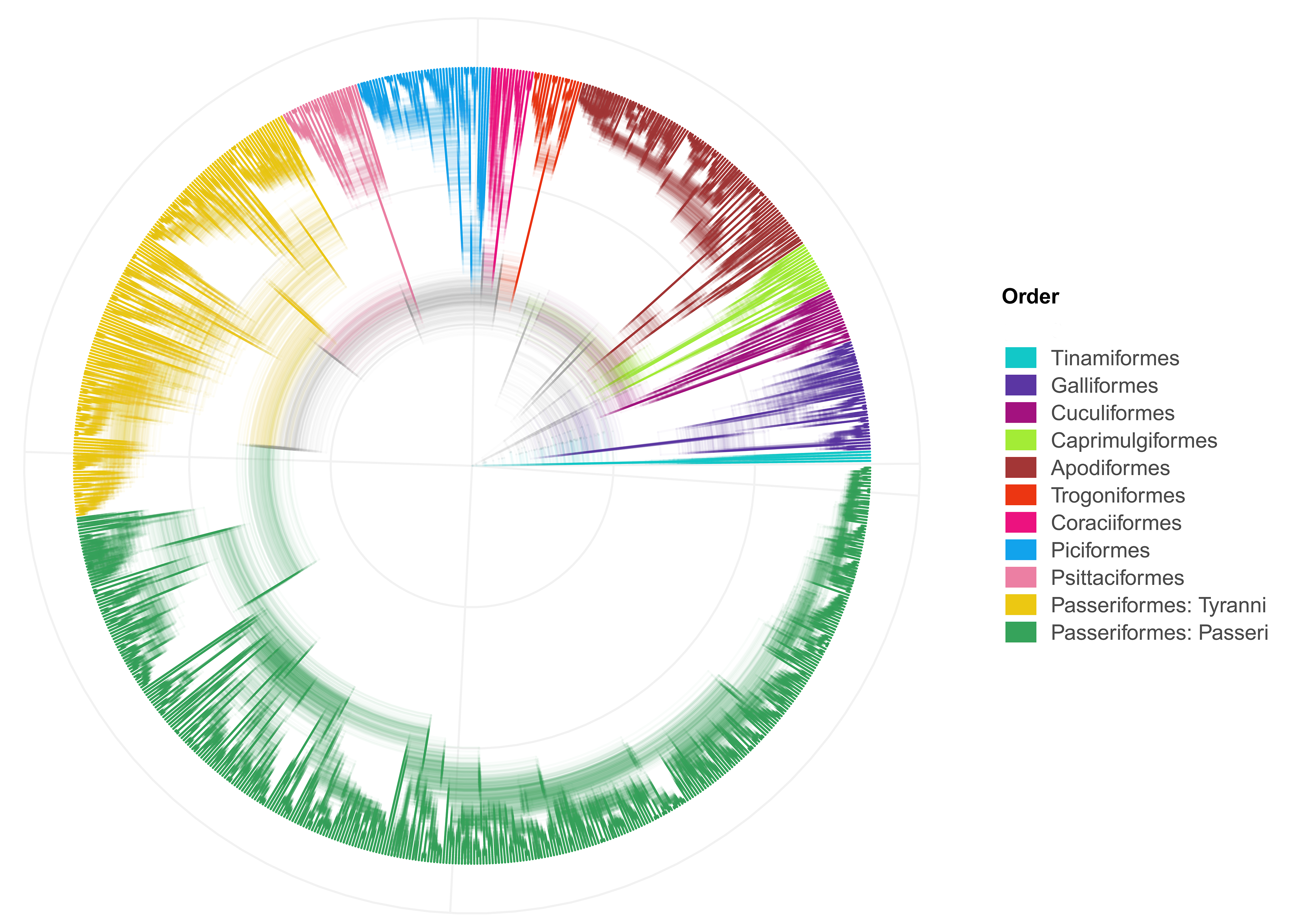

Supplement: Supplemental Information 4 — It includes 1000 trees with the selected species. [file peerj-12-16664-s004.png]
